# Expanding the Reticular Chemistry Building Block Library toward Highly Connected Nets: Ultraporous MOFs Based on 18-Connected Ternary, Trigonal Prismatic Superpolyhedra

**DOI:** 10.1021/jacs.3c12679

**Published:** 2024-03-01

**Authors:** Konstantinos G. Froudas, Maria Vassaki, Konstantinos Papadopoulos, Constantinos Tsangarakis, Xu Chen, William Shepard, David Fairen-Jimenez, Christos Tampaxis, Georgia Charalambopoulou, Theodore A. Steriotis, Pantelis N. Trikalitis

**Affiliations:** § Department of Chemistry, University of Crete, Heraklion 71003, Greece; ⊥ Department of Chemical Engineering & Biotechnology, University of Cambridge, Philippa Fawcett Drive, Cambridge CB3 0AS, U.K.; † Synchrotron SOLEIL-UR1, L’Orme des Merisiers, Saint-Aubin, BP 48, Gif-Sur-Yvette 91192, France; ‡ National Center for Scientific Research “Demokritos”, Athens 15341, Greece

## Abstract

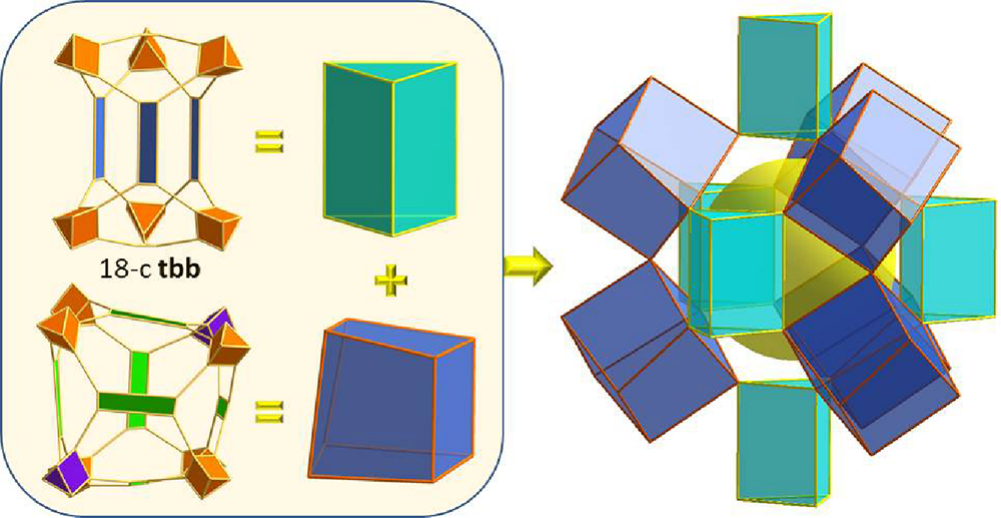

The chemistry of metal–organic frameworks (MOFs) continues to expand rapidly, providing materials with diverse structures and properties. The reticular chemistry approach, where well-defined structural building blocks are combined together to form crystalline open framework solids, has greatly accelerated the discovery of new and important materials. However, its full potential toward the rational design of MOFs relies on the availability of highly connected building blocks because these greatly reduce the number of possible structures. Toward this, building blocks with connectivity greater than 12 are highly desirable but extremely rare. We report here the discovery of novel 18-connected, trigonal prismatic, ternary building blocks (**tbb**'s) and their assembly into unique MOFs, denoted as Fe-**tbb**-MOF-*x* (*x*: 1, 2, 3), with hierarchical micro- and mesoporosity. The remarkable **tbb** is an 18-c supertrigonal prism, with three points of extension at each corner, consisting of triangular (3-c) and rectangular (4-c) carboxylate-based organic linkers and trigonal prismatic [Fe_3_(μ_3_-Ο)(−COO)_6_]^+^ clusters. The **tbb**’s are linked together by an 18-c cluster made of 4-c ligands and a crystallographically distinct Fe_3_(μ_3_-Ο) trimer, forming overall a 3-D (3,4,4,6,6)-c five nodal net. The hierarchical, highly porous nature of Fe-**tbb**-MOF-*x* (*x*: 1, 2, 3) was confirmed by recording detailed sorption isotherms of Ar, CH_4_, and CO_2_ at 87, 112, and 195 K, respectively, revealing an ultrahigh BET area (4263–4847 m^2^ g^–1^) and pore volume (1.95–2.29 cm^3^ g^–1^). Because of the observed ultrahigh porosities, the H_2_ and CH_4_ storage properties of Fe-**tbb**-MOF-*x* were investigated, revealing well-balanced high gravimetric and volumetric deliverable capacities for cryoadsorptive H_2_ storage (11.6 wt %/41.4 g L^–1^, 77 K/100 bar–160 K/5 bar), as well as CH_4_ storage at near ambient temperatures (367 mg g^–1^/160 cm^3^ STP cm^–3^, 5–100 bar at 298 K), placing these materials among the top performing MOFs. The present work opens new directions to apply reticular chemistry for the construction of novel MOFs with tunable porosities based on contracted or expanded **tbb** analogues.

## Introduction

Metal–organic frameworks (MOFs) represent an important class of functional porous materials made of the combination of inorganic and organic building blocks, linked together by coordination bonds.^1,2^ The use of diverse inorganic and organic components has led to the discovery of crystalline MOFs with exceptional porosity in terms of surface area and pore volume, as well as framework functionalities mainly originating from ligand modifications. For these reasons, MOFs are highly suitable for important applications including gas storage/separation and catalysis, among others.^3−7^

A key element in this field toward the rational design of advanced MOFs is the reticular chemistry approach, based on which both inorganic and organic building blocks are considered as well-defined geometric shapes with a particular connectivity (nodes).^8−11^ Accordingly, the MOF framework is represented by a net composed of one or more nodes. The particular geometric characteristics of these nodes and their connectivity, defined as the number of connections and their relative arrangement, determine the so-called net topology. The reticular chemistry structure resource (RCSR) contains a large collection of existing and hypothetical nets based on particular building blocks.^12^

In RCSR, the edge-transitive (one kind of edge) uninodal and binodal nets with different topologies represent an important selection because many of these can be targeted experimentally following a high level design.^8,10,13^ Coordination chemistry offers well-defined metal-based clusters in terms of geometry and connectivity that could be combined with organic ligands of suitable geometric shape and linking topology to readily afford a desired structure. Of prime importance are highly connected metal clusters because their increased connectivity greatly reduces the number of compatible structures associated with different nets.^14,15^ Therefore, with an appropriate choice of organic linkers, these highly connected clusters are suitable for the design and construction of novel MOFs based on a particular net topology. Representative examples include the hexanuclear Zr_6_(μ_3_-O)_4_(μ_3_-OH)_4_(−COO)_12_ and RE_6_(μ_3_-OH)_8_(−COO)_12_ as well as nonanuclear [RE_9_(μ_3_-OH)_12_(μ_3_-O)_2_(−COO)_12_] clusters (RE: rare earth) that have been successfully used for the targeted synthesis of highly connected MOFs based on **fcu**,^16^**ftw**,^17,18^**shp**,^19,20^ and **alb**(20,21) nets.

Importantly, highly connected clusters provide unique opportunities that expand the reticular chemistry toolbox by introducing advanced concepts such as the derived and related nets from parent nets,^22^ merged nets,^23^ and minimal edge-transitive nets.^24,25^ However, getting access into metal clusters with connectivity higher than 12 is extremely difficult. In fact, there is only one example of an 18-connected (18-c) cluster discovered through the exploratory reticular synthesis of **gea**-MOF-1 in which [Y_9_(μ_3_-OH)_8_(μ_2_-OH)_3_(−COO)_18_] clusters are linked with a triangular organic linker forming a (3,18)-connected net with **gea** topology.^26^ The novel topology of **gea**-MOF-1 was then used as a blueprint for the designed synthesis of **gea**-MOF-2 where an 18-c metal organic polyhedron (MOP) was used as a supermolecular building block. MOPs provide an alternative approach in MOF synthesis toward highly connected nets, where a representative example is the **rht** net based on a 24-c MOP.^27,28^

We report herein the discovery of novel isoreticular MOFs based on a mixed linker strategy using a one-pot synthesis that features a unique ternary, 18-c trigonal prismatic metal organic polyhedron constructed from six Fe_3_(μ_3_-Ο)(−COO)_6_ clusters, two 3-c trigonal, and three 4-c rectangular carboxylate-based organic linkers. This novel trigonal prismatic, ternary building block (**tbb**), in contrast to most of the known MOPs,^29^ does not require bend linkers to form and, as we explain below, represents an important addition to the reticular chemistry toolbox toward novel structures with unique topologies. The new five-nodal net MOFs, (3,4,4,6,6)-c, denoted as Fe-**tbb**-MOF-*x* (*x*: 1, 2, 3), display an unprecedented hierarchical pore system, including mesoporous cages with 30 Å in diameter, resulting in an ultrahigh accessible porosity with a total pore volume up to 2.29 cm^3^ g^–1^. Because of their unique structural features coupled with the remarkable porosity, these materials are considered highly promising for gas storage applications. Accordingly, the cryogenic hydrogen and, at near ambient conditions, methane storage properties were investigated in detail, revealing an excellent performance in terms of balanced gravimetric and volumetric working capacities.

## Results and Discussion

The trigonal prismatic 6-c trimeric oxo-centered carboxylate-based metal cluster with the general formula M_3_(μ_3_-Ο)(−COO)_6_ (M: Sc^3+^, V^3+^, Cr^3+^, Fe^3+^, Al^3+^, In^3+^) is an important and highly versatile secondary building unit (SBU) that has captured the attention in MOFs and related materials since the early stages of the field and continues to play an important role in the designed synthesis of advanced MOFs.^13,30^ Representative examples include the IRMOP series,^31^ the iconic MOFs MIL-100^32^ (**mtn-e-a** net) and MIL-101^33^ (**moo-a** net) structures with zelolite-like topologies, **pacs**-MOFs,^34^**soc**-MOFs,^35−37^ and **acs**-MOFs.^38−40^ Although this kind of trimeric SBUs has been combined, for example, with linear, triangular (3-c) or rectangular (4-c) carboxylate-based ligands or a combination of linear with 3-c triangular linkers, to the best of our knowledge, there is no report where M_3_(μ_3_-Ο)(−COO)_6_ units are combined with triangular 3-c and rectangular 4-c ligands to form a crystalline MOF. Furthermore, looking carefully in RCSR and the topology related literature, we were unable to identify a minimal edge transitive net (transivity [32]) made of 6-c trigonal prismatic, triangular 3-c, and rectangular 4-c building blocks.^22,25,41^ Therefore, this particular combination provides great opportunities through exploratory synthesis to enrich the reticular chemistry repertoire with novel MOFs displaying unprecedented topologies.

**Figure 1:**
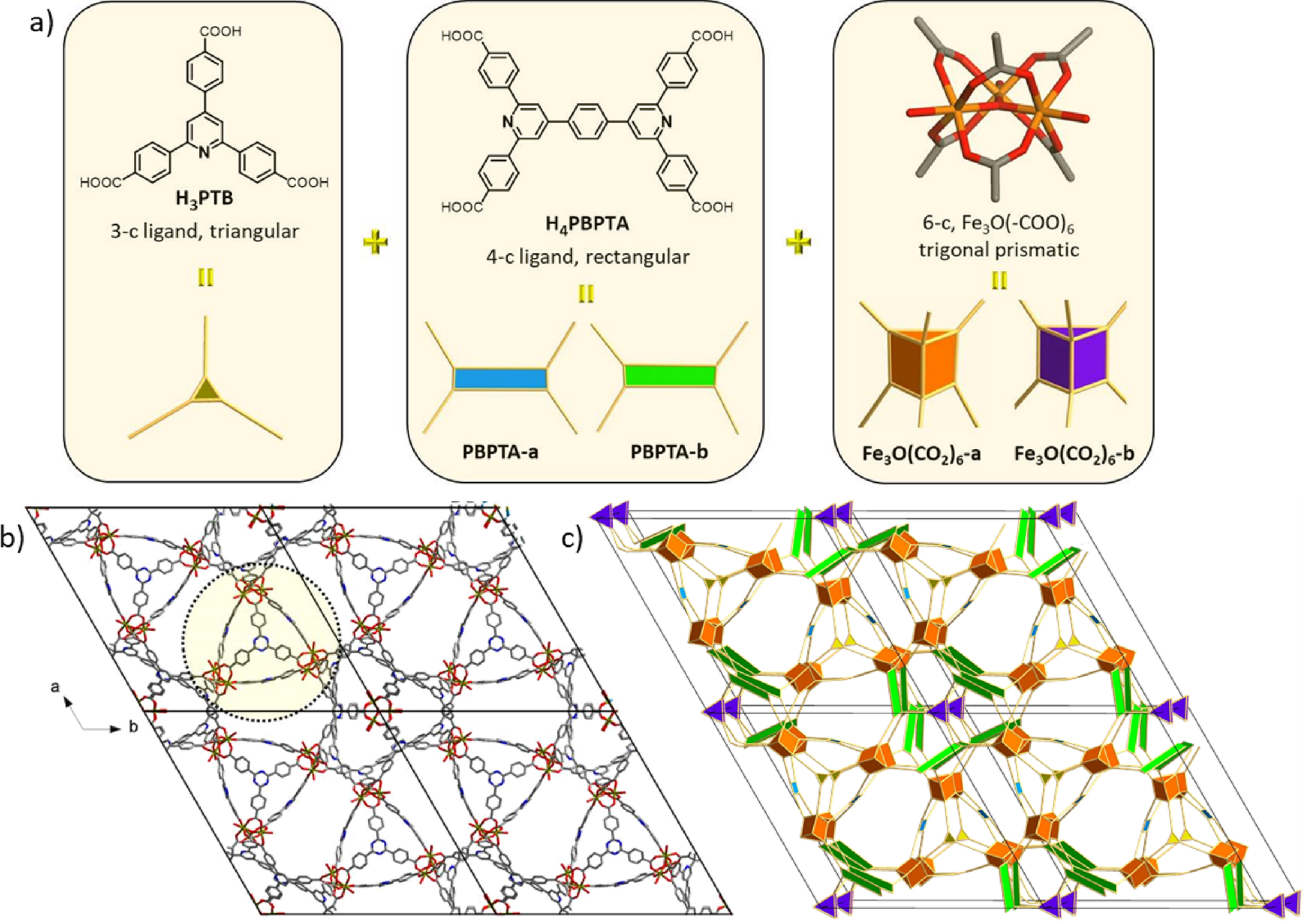
(a) The combination of 3-c triangular H_3_PTB and 4-c rectangular H_4_PBPTA organic linkers with 6-c trigonal prismatic Fe_3_(μ_3_-Ο)(−COO)_6_ clusters resulted in the formation of Fe-**tbb**-MOF-1. Their geometric representation used for the topological analysis is shown, where crystallographically distinct components have different colors. (b) The structure of Fe-**tbb**-MOF-1 looking down the *c* axis. The circle highlights the novel trigonal prismatic tertiary building block, seen from the top, made of six Fe_3_(μ_3_-Ο)(−COO)_6_ clusters, two PTB^3–^, and three PBTA^4–^ ligands (see the text for details). (c) The unique five nodal, (3,4,4,6,6)-c, augmented **tbb**-a net derived from the topological analysis of Fe-**tbb**-MOF-1.

The first member of the isoreticular series, denoted as Fe-**tbb**-MOF-1, was constructed by combining the 3-c triangular ligand 4,4′,4″-(pyridine-2,4,6-triyl)tribenzoic acid (H_3_PTB) and the 4-c rectangular ligand 4,4′,4″,4”’-(1,4-phenylenebis(pyridine-4,2–6-triyl))-tetrabenzoic acid (H_4_PBPTA) with the 6-c Fe_3_(μ_3_-O)(−COO)_6_ clusters and is shown in Figure 1. Accordingly, the solvothermal reaction in DMF of Fe(NO_3_)_3_·9H_2_O, H_3_PTB, and H_4_PBPTA in the presence of acetic acid, under controlled reaction conditions, afforded Fe-**tbb**-MOF-1 in its pure form. Initially, Fe-**tbb**-MOF-1 was obtained as large yellow hexagonal rod-like crystals, in a mixture with orange single crystalline cubes of Fe-**soc**-MOF, reported as Fe-pbpta, based only on PBPTA^4–^ ligand.^36^ By adjustment of the H_3_PTB/H_4_PBPTA molar ratio, a pure phase was obtained as confirmed by powder X-ray diffraction (PXRD) and scanning electron microscopy imaging (SEM), discussed below. The initial screening of the large hexagonal yellow crystals using an in-house single-crystal X-ray diffraction (SCXRD) instrument equipped with a Cu Kα microfocus source (Bruker D8 Venture) resulted in low-resolution data from which, however, a large hexagonal unit cell was obtained (Figure S14a). High-quality SCXRD data were collected by using synchrotron radiation from which the crystal structure was determined (Table S1).

Accordingly, Fe-**tbb**-MOF-1 crystallizes in the hexagonal system, space group *P-*62*c*, with large unit cell parameters *a* = *b* = 42.0981 Å, *c* = 61.5509 Å, α = β = 90^ο^, and γ = 120^ο^. The structure features two crystallographically distinct Fe_3_(μ_3_-O)(−COO)_6_, clusters, denoted as Fe_3_O(CO_2_)_6_-a and Fe_3_O(CO_2_)_6_-b; two deprotonated PBPTA^4–^ ligands, denoted as PBPTA-a and PBPTA-b; and a crystallographically unique deprotonated PTB^3–^ ligand. Each metal cluster is charge balanced by one NO_3_^–^ anion, which was found to be crystallographically disordered. Within an Fe_3_O(−COO)_6_ cluster, each Fe^3+^ has an octahedral geometry with an apical position occupied by one solvent molecule (e.g., H_2_O or DMF).

**Figure 2:**
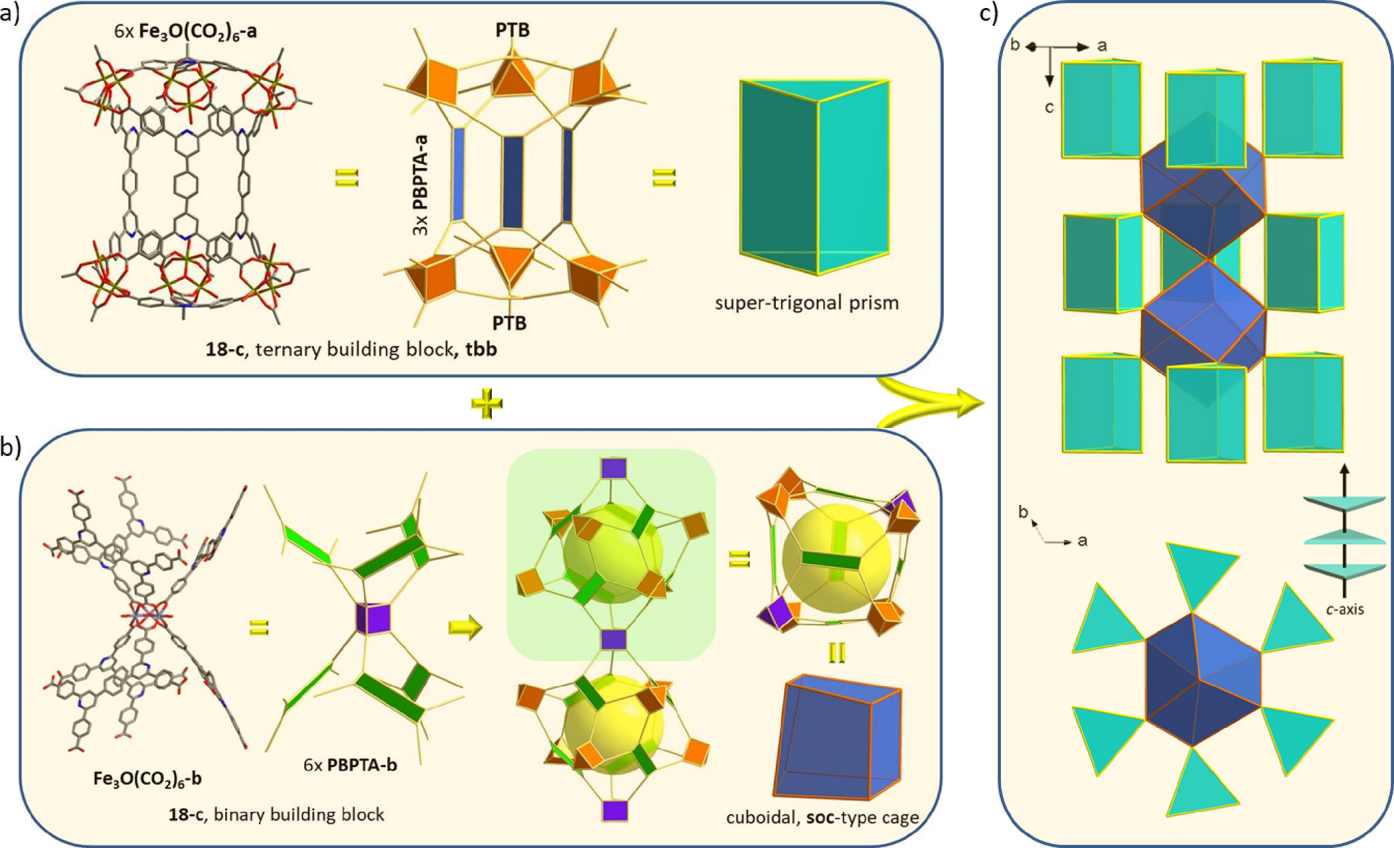
(a) The novel, supertrigonal prismatic (STP), ternary building block, **tbb**, observed in Fe-**tbb**-MOF-1 made of six Fe_3_O(CO_2_)_6_-a, two 3-c PTB, and three 4-c PBPTA-a crystallographically distinct components. (b) The distinct 18-c binary building block made of one Fe_3_O(CO_2_)_6_-b and six 4-c PBPTA-b crystallographically unique components. This 18-c unit connects nine **tbb** units, forming along the *c* axis corner-shared distorted, **soc**-type cuboidal cages. (c) Packing of the two distinct superpolyhedra observed in Fe-**tbb**-MOF-1, looking perpendicular to the *ac* plane (top) and down the *c* axis (bottom).

A novel ternary (made of three distinct components), trigonal prismatic building block is formed by the combination of six Fe_3_O(CO_2_)-a clusters located at the corners of the prism and linked together by three PBPTA-a and two PTB ligands, as shown in Figure 2a. This ternary building block, denoted as **tbb**, displays 18 points of extension, three in each corner, forming a unique 18-connected (18-c) **tbb**. Because of the size of the organic linkers constructing the **tbb,** this is in fact an elongated cage with an internal size of 8 Å × 16 Å (Figures 2a and 4d). The 18-c **tbb**’s are connected together by a different 18-c binary (made of two distinct components) building block formed by one Fe_3_O(CO_2_)-b cluster and six PBPTA-b ligands (Figure 2b). The latter 18-c building block connects nine 18-c **tbb**'s arranged in sets of three, where each set forms a triangle, and these triangles are placed in a staggered fashion along the *c* axis, as shown in Figure 2c. This particular connectivity results in the formation of corner-shared distorted cuboidal cages, with an internal size of 16 Å, made of six Fe_3_O(CO_2_)-a, two Fe_3_O(CO_2_)-b, and six PBPTA-b ligands (Figures 2b,c and 4d). Notably, these cages are related to those found in the **soc**-type MOF, denoted as Fe-pbpta, formed exclusively by PBPTA ligands, where regular edge shared cubes are observed (Figure S16).^36^ The origin of the distortion in Fe-**tbb**-MOF-1 can be found in the relative arrangement of the PBPTA ligands occupying the neighboring cuboidal faces. Specifically, in contrast to the **soc**-MOF where neighboring PBPTA ligands are rotated by 90°, in Fe-**tbb**-MOF-1, the pairs of neighboring faces have the same ligand orientation (Figure S16b). The overall charge balanced chemical formula of Fe-**tbb**-MOF-1 is (Fe_3_O)_7_(PBPTA)_9_(PTB)_2_(NO_3_)_7_. For the topological analysis using the software ToposPro,^42^ the geometric representation of crystallographically distinct components (metal clusters and linkers), highlighted with different colors, is shown in Figure 1a. These geometric building blocks were simplified to the corresponding nodes and, in particular, the 6-c Fe_3_O(CO_2_)_6_-**a** and Fe_3_O(CO_2_)_6_-**b** clusters to 6-c trigonal prismatic nodes, the 4-c PBPTA-**a** and 4-c PBPTA-**b** linkers to 4-c rectangular nodes, and the 3-c PTB ligand to a 3-c trigonal node, resulting to the unique five nodal (3,4,4,6,6)-c **tbb** net (Figure S20). Alternatively, if the 4-c linkers are considered as interconnected 3-c trigonal building units, a derived six-nodal (3,3,3,3,6,6)-c **tft** net is obtained (Figure S21).

**Figure 3:**
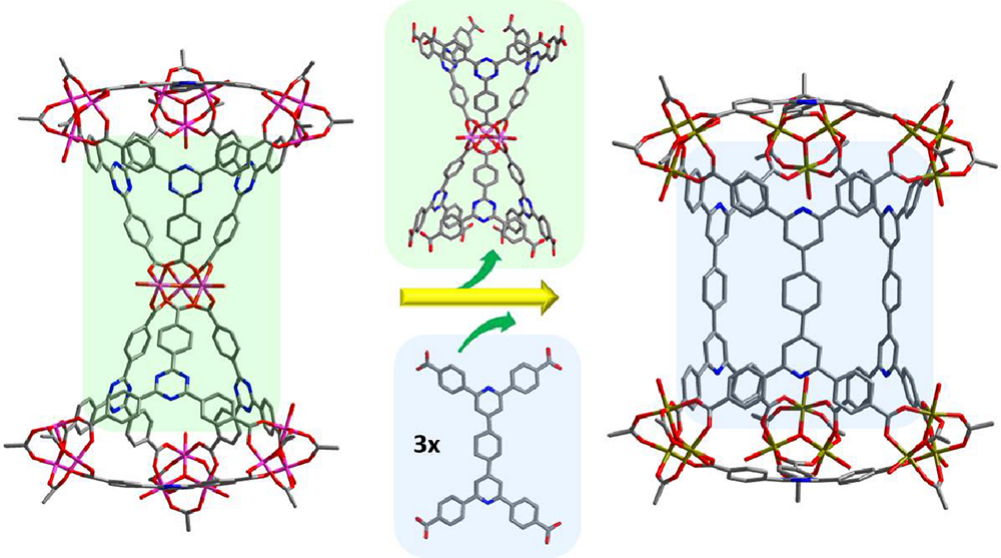
Relation between two corner-shared supertetrahedra (ST) found in extended MIL-100 type structures (left) and the novel **tbb** unit (right) observed in Fe-**tbb**-MOF-1: **tbb** can be viewed as the product of the topotactic replacement of the corner shared M_3_O(TATB)_6_ unit with three 4-c PBPTA ligands in two corner-shared ST found in PCN-333.

Looking carefully at the structure of the novel **tbb**, it is important to make a comparison with the corner-shared supertetrahedra (ST) observed in the extended versions of MIL-100 type structures based on the triangular linkers 4,4′,4″-benzene-1,3,5-triyl-tribenzoate (BTB)^43^ and 4,4′,4″-*s*-triazine-2,4,6-triyl-tribenzoate (TATB)^44^ that have the same size as PTB. Figure 3 shows two merged, corner-shared ST found in PCN-333^44^ based on TATB along with the novel **tbb** observed in Fe-**tbb**-MOF-1. It is evident that these two superpolyhedra are related by a topotactic replacement of the corner-shared unit M_3_(μ_3_-Ο)(TATB)_6_ with three 4-c PBPTA ligands, each occupying a rectangular face of the resulting trigonal prism. In other words, **tbb** can be viewed as a fusion product of two corner-shared ST atoms found in the extended versions of MIL-100. Considering the 4-c PBPTA as two merged triangles (Figure S21), the size match between these triangles and PTB apparently facilitates the formation of the novel **tbb** cage. It is important to note that the combination of the linear 1,4-benzenedicarboxylate (bdc) and the triangular btb linkers in MIL-142A results in the formation of a superoctahedron and not a supertrigonal prism as observed in **tbb** (Figure S17).^45,46^

The novel 18-c trigonal prismatic **tbb** and its relation to corner-shared ST based on M_3_(μ_3_-Ο)(−COO)_6_ clusters described above provide great opportunities to apply reticular chemistry for the construction of contracted or expanded **tbb** analogues and their targeted assembly toward novel MOFs with a particular net topology. For example, the smaller triangular linkers benzene-1,3,5-tricarboxylate (BTC) and benzo-tris-thiophene carboxylate (BTTC) form corner-shared ST as observed in **mtn** MOFs MIL-100^32^ and PCN-332,^44^ respectively. These smaller 3-c linkers could be combined with suitable 4-c linkers consisting of two bridged 1,3-benzene dicarboxylate units, as for example in 3,3′,5,5′-azobenzenetetracarboxylate (ABTC), to form a contracted **tbb** analogue. It is noted that ABTC forms **soc** type MOFs.^47^ On the other hand, diverse and expanded corner-shared ST are reported in **moo** MOFs such as MOF-919,^48^ MOF-929,^49^ and MOF-939^49^ made with the triangular copper-based metallo-ligand using pyrazole and its extended derivatives. Therefore, this kind of triangular linkers could be combined with suitable 4-c linkers for the assembly of large **tbb** analogues. Notably, the pyrazole assembled copper-based triangular metallo-ligands form very similar corner-shared ST based on 6-c Zr_6_-clusters with trigonal antiprismatic connectivity, as observed in MOF-818 with **spn** topology.^48^ Therefore, it is entirely possible that Zr-based **tbb** could also be constructed as shown in Figure S18, further expanding the library of these unique building blocks. In terms of the designed synthesis of MOFs using different **tbb** units, as an example, we envisioned the construction of **acs** type MOFs where the formation of corner bridged **tbb**’s could be facilitated by three suitable bridging linkers at each corner (each **tbb** corner has three points of extension) displaying the required positioning of their carboxylate groups, as 4,4′-dicarboxydiphenyl sulfone (DCDPS) and benzene-1,3-dimesitylenic acid (BDM) used to construct PCN-133^50^ and Zr-**sod**-ZMOFsb^51^ respectively (Figure S19). The above reticular chemistry-guided synthetic approaches toward expanding the library of different **tbb**’s and their assembly into novel MOFs are currently explored in our group.

**Figure 4:**
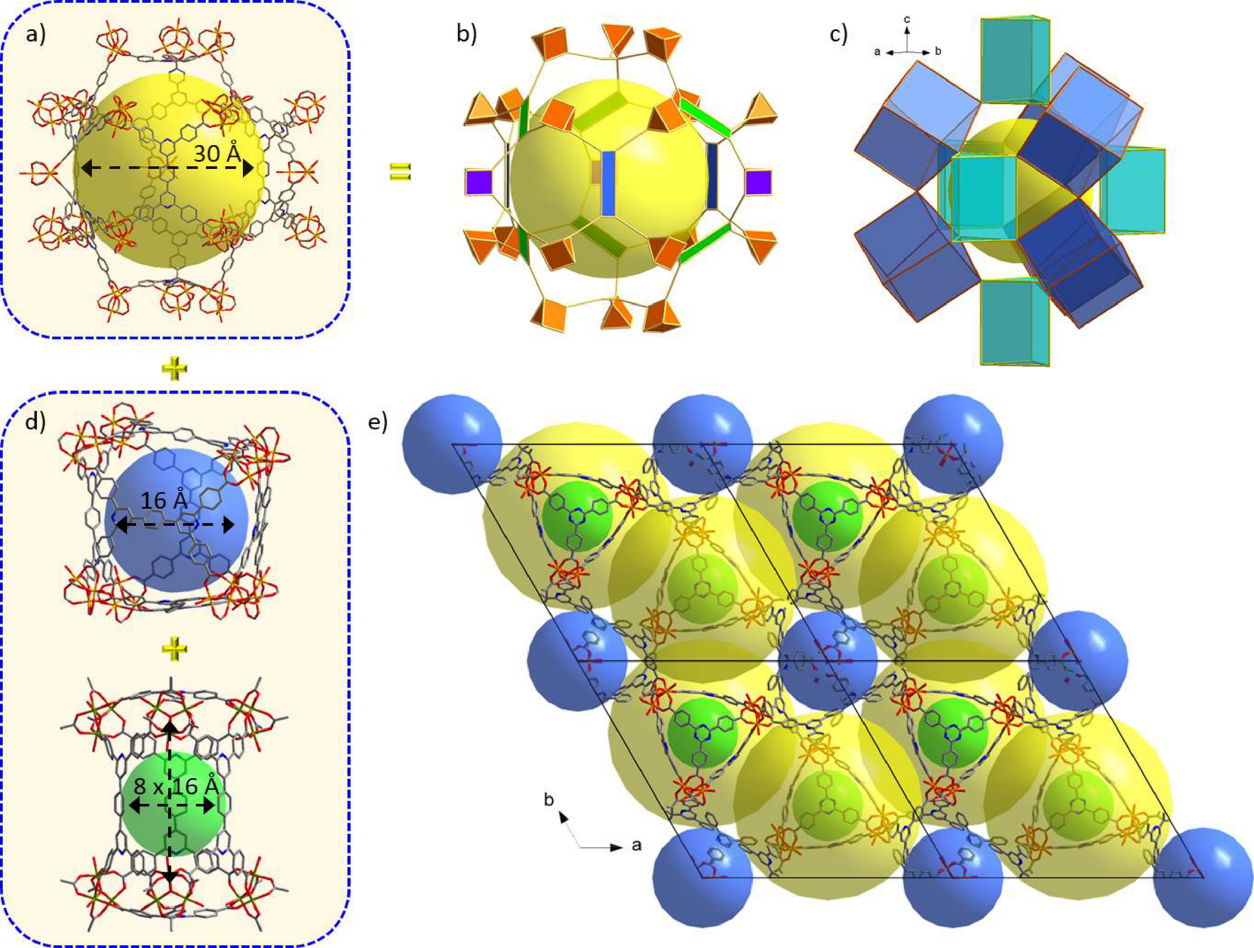
(a) The large mesoporous cage in Fe-**tbb**-MOF-1 formed by 21 Fe_3_O(−COO)_6_ clusters, 9 PBPTA^4–^, and 2 PTB^3–^ ligands. (b) Geometric representation of the crystallographically distinct building units that form the mesoporous cage. (c) Packing arrangement of the cuboidal **soc**-type and trigonal prismatic **tbb** cages around the mesopore cage. (d) The two distinct microporous polyhedral cages, **soc**-type (top) and **tbb** (bottom), are combined with the mesoporous cages in Fe-**tbb**-MOF-1, forming a unique network of interconnected cages with hierarchical porosity, as shown in panel e.

The particular connectivity of **tbb** and the cuboidal cages in Fe-**tbb**-MOF-1 results in the formation of mesoporous cages of approximately 30 Å in diameter. As shown in Figure 4a, a large mesoporous polyhedral cage is formed consisting in total of 21 Fe_3_O(−COO)_6_ clusters, 9 PBPTA^4–^, and 2 PTB^3–^ ligands. Looking at the crystallographically unique components, the mesoporous cage is made of 18 Fe_3_O(CO_2_)_6_-a, 3 Fe_3_O(CO_2_)_6_-b, 6 PBPTA-a, 3 PBPTA-b, and 2 PTB (Figure 4b). Considering the formation of **soc**-type cuboidal cages as well as the trigonal prismatic **tbb** cages, their particular arrangement around the mesopore cage is shown in Figure 4c. Accordingly, the cage is located above and below each **tbb** cage along the *c* axis, surrounded by three more **tbbs** forming a triangle in an *ab* plane and six **soc**-type cuboids in a trigonal prismatic arrangement (Figure 4c,e). Therefore, the resulting three-dimensional open framework of Fe-**tbb**-MOF-1 is made of interconnected hierarchical cages in the micro- (**tbb**), supermicro- (**soc**-type), and mesopore range. This unique pore system was found fully accessible by different gases, as we describe in detail below. The phase purity of Fe-**tbb**-MOF-1 is confirmed by comparing the corresponding experimental powder X-ray diffraction pattern (PXRD) with that calculated from the single-crystal structure (Figure S22). Furthermore, the ^1^H NMR spectrum of an acid digested sample revealed the presence of PBPTA and PTB ligand at a 9:2 molar ratio, in full agreement with the crystallographic data and the chemical formula (Figure S25). In addition, SEM images reveal the formation of hexagonal single crystals and the absence of cubes, which are characteristic of the **soc**-type structure (Figure S10).

The discovery of Fe-**tbb**-MOF-1 provides a novel platform for the isoreticular synthesis of new compounds. To demonstrate this, instead of PTB, we used the triangular linkers H_3_TATB and H_3_BTB in one-pot syntheses with H_4_PBPTA and isolated the isostructural MOFs, Fe-**tbb**-MOF-2 and Fe-**tbb**-MOF-3, respectively. Also, in these cases, phase purity was confirmed by PXRD and ^1^H NMR measurements (Figures S22, S26, and 27). Furthermore, SEM images show the formation of hexagonal single crystals (Figures S11 and S12). For Fe-**tbb**-MOF-3, large single crystals were isolated from which the unit cell was determined using an in-house SCXRD instrument, which confirmed the formation of an isostructural phase (Figure S14b). In addition, the isostructural Al-based analogue Al-**tbb**-MOF-1 was also isolated, however, as a mixed phase with the corresponding Al-**soc**-MOF^37^ as confirmed by SEM (Figure S13) and PXRD (Figure S23). Attempts to modify the synthetic conditions to obtain a phase pure material were unsuccessful; however, pure Al-**ttb**-MOF-1 was obtained by performing a postsynthetic metal exchange reaction, as confirmed by PXRD and SEM/EDS analysis (Figure S24). These results will be published in detail elsewhere.

**Figure 5:**
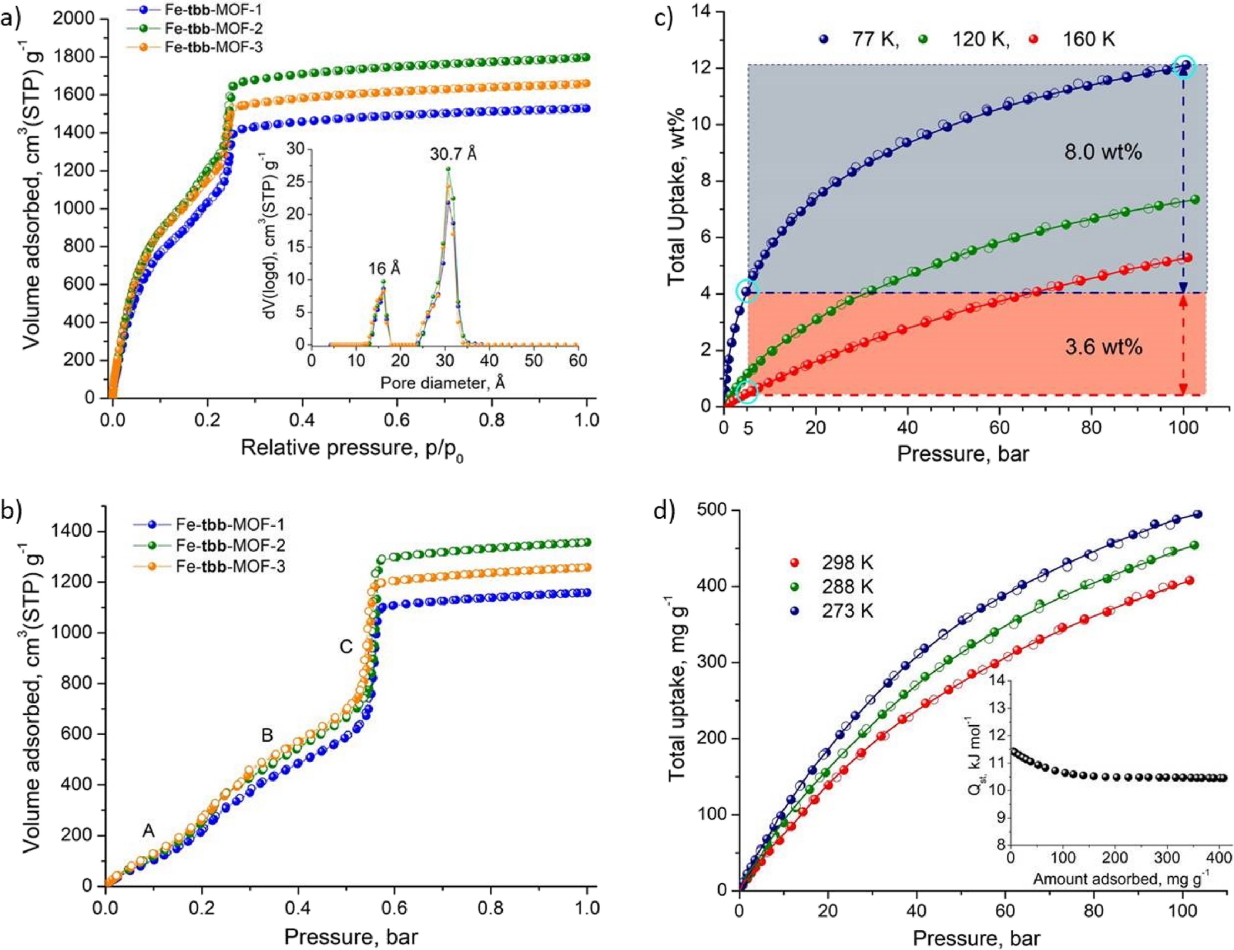
(a) Argon sorption isotherms of Fe-**tbb**-MOF-*x* (*x*: 1, 2, 3) recorded at 87 K. The inset shows the corresponding NLDFT calculated PSD curves. (b) Carbon dioxide isotherms recorded at 195 K showing distinct adsorption regions, A, B and C, associated with different pore sizes. (c) Hydrogen sorption isotherms of Fe-**tbb**-MOF-2 at cryogenic conditions indicating the corresponding gravimetric working capacities under isothermal pressure swing (gray area) and pressure–temperature swing (gray and orange area). (d) Methane sorption isotherms of Fe-**tbb**-MOF-2 recorded at near ambient temperatures up to 100 bar. The inset shows the corresponding isosteric heat of the adsorption curve as a function of the amount adsorbed.

The hierarchical porosity in the Fe-**tbb**-MOF-*x* (*x*: 1, 2, 3) series was confirmed by accurate gas sorption measurements, recording isotherms of Ar, CH_4_, and CO_2_ at 87, 112, and 195 K, respectively. All MOFs were successfully activated by replacing DMF solvent molecules in as-made samples with acetonitrile followed by overnight evacuation under ultrahigh vacuum at 80 °C. The corresponding Ar and CO_2_ sorption isotherms of the Fe-**tbb**-MOF-*x* (*x*: 1, 2, 3) series recorded at 87 and 195 K, respectively, are shown in Figure 5. The Ar isotherms show progressive adsorption up to 0.1 *p*/*p*_0_ where a rounded knee is observed, associated with micropore filling, followed by an almost linear increase in uptake up to 0.24 *p*/*p*0 where a sharp capillary condensation step is observed, indicating the presence of mesopores. Pore size distribution (PSD) curves calculated using a suitable nonlocal DFT kernel (NLDFT) reveal two distinct peaks centered at 16 and 30.7 Å, demonstrating the presence of hierarchical porosity in the Fe-**tbb**-MOF-*x* (*x*: 1, 2, 3) series, in full agreement with single-crystal data (inset in Figure 5a). The hierarchical porosity is also nicely captured in the corresponding CO_2_ isotherms recorded at 195 K, where before the sharp capillary condensation step (marked as C in Figure 5b), two well-defined adsorption regions (A and B), associated with microporosity, are observed (Figure 5b). It is noted that the pore space in Fe-**tbb**-MOF-*x* (*x*: 1, 2, 3) is highly complex because of the interconnected cages with different sizes and shapes, where the large mesoporous cage is surrounded by the microporous **tbb** and the supermicroporous **soc**-type cages (Figure 4c), as has been described in the single-crystal structure analysis section.

BET area calculations were performed using the recently reported BET surface identification algorithm (BETSI) that extends the Rouquerol consistency criteria.^52^ The use of this algorithm is critical for an unambiguous BET assignment in materials with hierarchical porosity such as Fe-**tbb**-MOF-*x* (*x*: 1, 2, 3), where microporosity is coupled with mesoporosity, resulting in isotherms with rounded knees and capillary condensation steps. Accordingly, the BET area is 4263, 4777, and 4847 m^2^ g^–1^ for Fe-**tbb**-MOF-1, Fe-**tbb**-MOF-2, and Fe-**tbb**-MOF-3, respectively (see Figures S28–S30). The corresponding total pore volume is 1.95, 2.29, and 2.11 cm^3^ g^–1^ at 0.99 *p*/*p*_0_, which is close to the calculated value from the single-crystal structure (2.7 cm^3^ g^–1^), indicating successful pore activation. It is important to note that the crystallographically calculated pore volume is overestimated because the framework counterions have not been located in the single-crystal structure because of their disordered nature. The relatively small variation in the observed total pore volumes between the three isostructural solids is not considered to be associated with the different 3-c organic linkers (PTB, TATB, and BTB in Fe-tbb-MOF-1, -2, and -3, respectively) but may rather reflect small differences in the degree of framework robustness between the three MOFs that could slightly affect pore activation in such ultraporous solids. Interestingly, an increased pore volume in the Fe-**tbb**-MOF-*x* series is associated with an increased temperature where the thermal decomposition is completed in the TGA curve (plateau), as we described below. The corresponding total pore volumes from CO_2_ as well as from CH_4_ isotherms, the latter recorded at 112 K (Table S2 and Figure S31), are very close to the values obtained from the Ar isotherm, indicating that the pore space of Fe-**tbb**-MOF-*x* is fully accessible to these molecules.

Thermogravimetric (TGA) analysis performed on solvent-free materials under a nitrogen atmosphere revealed a good thermal stability with a major weight loss starting at 300 °C for Fe-**tbb**-MOF-1 and 345 °C for Fe-**tbb**-MOF-*x* (*x*: 2, 3) (Figure S32). Interestingly, the thermal decomposition profile is shifted toward higher temperatures in the order Fe-**tbb**-MOF-2 > Fe-**tbb**-MOF-3 > Fe-**tbb**-MOF-1, indicating differences in the relative thermal stability of the materials, presumably due to the different chemical compositions, associated with the 3-c ligand. Notably, the TGA trace of Fe-**tbb**-MOF-2 (3-c linker is TATB) reaches a plateau at 700 °C, where for Fe-**tbb**-MOF-3 (3-c linker is BTB) and Fe-**tbb**-MOF-1 (3-c linker is PTB) the plateau is observed at 600 and 527 °C, respectively. The observed differences suggest an enhanced framework robustness in the order Fe-**tbb**-MOF-2 > Fe-**tbb**-MOF-3 > Fe-**tbb**-MOF-1. Interestingly, this is the order of the increased pore volume of the corresponding activated materials. The calculated weight losses for Fe-**tbb**-MOF-2 and Fe-**tbb**-MOF-3 are 80.25 and 80.85% respectively, which are very close to the calculated value from their chemical formula (81.3%), assuming the formation of Fe_2_O_3_. However, for Fe-**tbb**-MOF-1, the corresponding value is relatively lower (70.44%) than the expected one. In this case, a possible missing linker defect cannot be ruled out.

The ultrahigh porosity of Fe-**tbb**-MOF-*x* is considered very important for gas storage applications.^53^ Accordingly, we investigated in detail the corresponding H_2_ and CH_4_ storage properties of Fe-**tbb**-MOF-*x* at cryogenic (H_2_) and near ambient (CH_4_) conditions and high pressures, which are relevant for real applications, with highly promising results, as we describe below.

High-pressure adsorption experiments were conducted volumetrically at 77, 120, and 160 K for H_2_ as well as at 273, 288, and 298 K for CH_4_ after proper outgassing (353 K, under high vacuum overnight) the acetonitrile exchanged as-made samples. For measurements at cryogenic temperatures, special attention has been given to volume calibrations, which were carried out in a way to completely avoid errors related to helium adsorption. The corresponding adsorption isotherms for the highest pore volume analogue, Fe-**tbb**-MOF-2, are presented in Figure 5c (pertinent data for Fe-**tbb**-MOF-1 are presented in Figure S34). It is noted that although the actual experimentally measured quantity is always the Gibbsian surface excess, the results are presented as total amounts adsorbed because this approach is more relevant for gas storage applications. Notably, the total H_2_ uptake at 77 K upon charging with 100 bar exceeds 12 wt % (137.5 mg of H_2_ per g of MOF), whereas the working capacities for isothermal pressure swing (100 to 5 bar at 77K) and pressure–temperature swing (77 K/100 bar to 160 K/5 bar) are calculated as 8.0 and 11.6 wt %, respectively (Figure 5b). Taking into account the bulk density of Fe-**tbb**-MOF-2, the latter corresponds to a working volumetric capacity of 41.4 g L^–1^. These well-balanced high gravimetric and volumetric deliverable capacities place Fe-**tbb**-MOF-2 in the list of top performing materials for cryoadsorptive hydrogen storage, including NU-1500-Al (8.2 wt %/44.6 g L^–1^), SNU-70 (10.6 wt %/47.9 g L^–1^), and NOTT-112 (9.1 wt %/41 g L^–1^) (see Table S3 and Figure S49).^53^ The H_2_ isosteric heat of adsorption (*Q*_st_) was calculated as a function of coverage (Figure S45) by simultaneous fitting of the adsorption isotherms by the virial equation, and Clausius–Clapeyron calculations were also performed. Both methods revealed a relatively weak H_2_-MOF interaction (3–4 kJ mol^–1^), which is moreover decreasing upon loading, as expected. It must be noted that such low heats of adsorption are considered ideal for cryoadsorptive gas storage for two reasons: (a) the amount of hydrogen “trapped” at the release pressure (5 bar) is minimized, and (b) temperature variations due to adsorption/desorption are minimized, and thus, heat management at the storage tank level is more facile.

The experimental total CH_4_ gravimetric working capacity of Fe-**tbb**-MOF-2 (Figure 5d) for a pressure swing between 5 and 100 bar at 298 K is 367 mg g^–1^, which is very close (75%) to the pertinent DOE target (0.5 g g^–1^); the working volumetric capacity is 160 cm^3^ STP cm^–3^. Notably, the current room temperature CH_4_ working (5–100 bar) volumetric and gravimetric capacity records are 251 cm^3^ STP cm^–3^ for MOF-519^54^ and 500 mg g^–1^ for NU-1501-Al,^39^ respectively. In this respect, Fe-**tbb**-MOF-2 reveals a well-balanced gravimetric–volumetric uptake, placing this material in the list of top performing MOFs, such as NU-1500-Al (0.29 g g^–1^/202 cm^3^ cm^–3^), MFU-4l-Li (0.33 g g^–1^/220 cm^3^ cm^–3^), and NPF-200 (0.38 g g^–1^/207 cm^3^ cm^–3^) measured under similar conditions (see Table S4 and Figure S50), whereas its CH_4_ storage potential is enhanced at lower temperatures, i.e., 402 and 434 mg g^–1^ (175 and 189 cm^3^ STP cm^–3^) for 288 and 273 K, respectively. Virial fitting of the adsorption isotherms led to rather modest gas–solid interactions (Figure 5d, inset) of 11.5 kJ mol^–1^, a value that is rapidly decreasing with increasing coverage to a plateau of around 10.5 kJ mol^–1^.

## Conclusions

In conclusion, we demonstrated that 3-c triangular and 4-c rectangular carboxylate-based linkers can be combined with 6-c M_3_(μ_3_-Ο)(−COO)_6_ (M: Fe^3+^, Al^3+^) clusters to form novel MOFs with ultrahigh hierarchical micro- and mesoporosity. In particular, the 3-c/4-c combination including PTB/PBPTA, TATB/PBPTA, and BTB/PBPTA with 6-c Fe_3_(μ_3_-Ο)(−COO)_6_ clusters led to the discovery of isostructural, highly connected (3,4,4,6,6)-c 5-nodal Fe-**tbb**-MOF-*x* (*x*: 1, 2, 3) solids in pure form following one-pot synthesis. A novel component in these structures is an 18-c ternary, cagelike, supermolecular trigonal prismatic building block (**tbb**)that is connected through an 18-c cluster, resulting in a unique hierarchical porous network made of discrete interconnected cages. This particular highly porous network in Fe-**tbb**-MOF-*x* is very well suited for important gas storage applications. Accordingly, the well-balanced high gravimetric and volumetric deliverable capacities for cryoadsorptive hydrogen storage, as well as methane storage at near ambient temperatures, are demonstrated for Fe-**tbb**-MOF-2, placing this material among the top performing MOFs.

Considering the formation of the unique 18-c supermolecular **tbb**, the present work opens new directions to apply reticular chemistry for the construction of contracted or expanded **tbb** analogues and their targeted assembly toward novel MOFs with a particular net topology, such as the **acs**. Importantly, new **tbb**’s could be assembled not only by M_3_(μ_3_-Ο)(−COO)_6_ units but also by trigonal antiprismatic 6-c hexanuclear Zr/Hf and lanthanide-based carboxylate clusters, greatly expanding and diversifying the corresponding chemical composition and associated framework properties of targeted new MOFs.

## Supporting Information Available

Experimental details, single-crystal and powder XRD data, SEM images, additional gas sorption isotherms, NMR, and TGA (PDF)
