# Supplementary material for: Expanding the Reticular Chemistry Building Block Library toward Highly Connected Nets: Ultraporous MOFs Based on 18-Connected Ternary, Trigonal Prismatic Superpolyhedra
Source: J Am Chem Soc. 2024 Mar 1;146(13):8961–70. doi: 10.1021/jacs.3c12679 (PMC10996011; doi:10.1021/jacs.3c12679)
Supplement: Supplementary file 1 — ja3c12679_si_001.pdf [file ja3c12679_si_001.pdf]

Expanding the reticular chemistry building block library towards highly connected nets: ultraporous MOFs based on 18-connected ternary, trigonal prismatic superpolyhedra

Konstantinos G. Froudas[§], Maria Vassaki[§], Konstantinos Papadopoulos[§], Constantinos Tsangarakis[§], Xu Chen[⊥], William Shepard[≠], David Fairen-Jimenez[⊥], Christos Tampaxis[‡], Georgia Charalambopoulou[‡], Theodore A. Steriotis[‡] and Pantelis N. Trikalitis[§,*]

[§]Department of Chemistry, University of Crete, 71003 Heraklion, Greece; [⊥]Department of Chemical Engineering & Biotechnology, University of Cambridge, Philippa Fawcett Drive, Cambridge CB3 0AS, UK; [≠]Synchrotron SOLEIL-UR1, L'Orme des Merisiers, Saint-Aubin, BP 48, 91192 Gif-Sur-Yvette, France; [‡]National Center for Scientific Research "Demokritos", 15341 Athens, Greece

SUPPORTING INFORMATION

Table of Contents

Methods and instrumentation S2

Organic synthesis and ^{1}H NMR spectra S4

MOFs syntheses S13

Scanning electron microscopy images (SEM) S14

Single crystal X-ray crystallography and additional structural figures S17

Topological analysis S21

Powder X-ray diffraction measurements S24

^{1}H NMR measurements of acid digested samples S26

BET area calculations using BETSI algorithm S28

Additional sorption isotherms S29

Thermal gravimetric analyses (TGA) S30

High pressure H_2 and CH_4 sorption isotherms and determination of isosteric heat of adsorption S31

References S43

Methods and Instrumentation

Starting Materials All chemicals were purchased commercially and were used without further purification. 4-Methylacetophenone 95%, Sodium Hydroxide 98% pellets (NaOH), Ethanol absolute (EtOH), Toluene ≥ 99.7%, Toluene anhydrous 99.8%, Nitric acid 65% (HNO_3), Sulphuric acid 95-98% (H_2SO_4), Hydrochloric acid ≥ 37% (HCl), Sodium bicarbonate ≥ 99.7% ($NaHCO_3$) were purchased from Aldrich. Cyanuric Chloride 98%, Acetic Anhydride 99+% (Ac_2O), 1,3,5-Triphenylbenzene 99+%, Magnesium Sulfate Anhydrous ≥ 99.5% ($MgSO_4$), Bromine 99.8% (Br_2) were purchased from Alfa Aesar. Terephthalaldehyde 98%, Chromium (VI) oxide 99% (CrO_3), p-tolualdehyde 98% were purchased from Thermo Fisher Scientific. Ammonium Acetate (NH_4Ac) was purchased from Scharlau. Aluminum chloride anhydrous 98% was purchased from Riedel-de Haën. Chloroform ≥ 99.8% ($CHCl_3$) was purchased from Fischer Chemicals. Acetic acid ≥ 99.8% (AcOH), Acetyl chloride ≥ 99%, Sodium Thiosulfate Pentahydrate ≥99.0% ($Na_2S_2O_3 \cdot 5H_2O$), 1,4-dioxane ≥ 99.8% were purchased from Honeywell Fluka. Petroleum ether ≥ 90%, Ethyl acetate ≥ 99.5% (EtOAc), dichloromethane ≥ 99.9% (CH_2Cl_2) were purchased from Honeywell Riedel-de Haën.

Powder X-Ray Diffraction Patterns were collected using a Panalytical X'pert Pro MPD System Cu Kα (λ=1.5418 Å) radiation operated at 45 kV and 40 mA. A typical scan rate was 3 sec/step with a step size of 0.02 deg.

Single Crystal X-Ray Diffraction
In house single crystal X-ray diffraction data were collected on a Bruker D8 Venture diffractometer equipped with a Cu Incoatec microfocus IμS 3.0 source, a Photon II detector operating in shutterless mode and a cryostrem 800 system (Oxford Cryosystems) for temperature regulation.

Synchrotron Single Crystal X-ray Diffraction was performed on the PROXIMA 2A micro-focused beamline in SOLEIL synchrotron (λ = 0.729319 Å) using an EIGER X9M 2D hybrid photon counting detector. Data were collected at 200 K. Subsequent data integration and reduction were undertaken with Xia2 (Winter, G., xia2: an expert system for macromolecular crystallography data reduction. J. Appl. Crystallogr. 2009, 43, 186-190). No corrections for solvent were applied. The structure was solved using the direct method and refined by full-matrix least-squares on F2 by the SHELXTL-2014 software package. The disorder was modeled using standard crystallographic methods, including constraints, restraints and rigid bodies where necessary. All carbon-bound hydrogen atoms were added in idealized positions and refined using a riding model. Several restraints were used to obtain reasonable parameters. H-atoms were refined isotropically, while the other atoms were refined anisotropically. All the phenyl rings are constrained to the ideal six-membered ring. The solvent molecules were highly disordered, and attempts to locate and refine the solvent peaks were unsuccessful. Contributions from the solvent molecules were removed using the SQUEEZE routine of PLATON, structures were then refined again using the data generated under Olex2-1.5 (Dolomanov, O. V.; Bourhis, L. J.; Gildea, R. J.; Howard, J. A. K.; Puschmann, H. J. Appl.Crystallogr. 2009, 42, 339-341). The contents of the solvent region were not represented in the unit cell contents in the crystal data. Crystal data and details of the data collection are given in Table S1.

Scanning electron microscopy (SEM) images were collected on a field emission JSM-IT700HR instrument.

^{1}H NMR spectra were recorded on a 500MHz Bruker spectrometer. MOF samples were prepared by digesting a small portion (~2mg) of the acetonitrile exchanged solids with a drop of concentrated HCl acid (37%) in a DMSO-d6 solution.

Gas sorption measurements at low pressures. Low Pressure nitrogen, argon, carbon dioxide and methane gas sorption measurements were carried at different temperatures up to 1 bar using Autosorb-

iQ2 instrument from Quantachrome equipped with a cryocooler system capable of temperature control from 20 to 320 K. Prior to analysis the as made samples were washed with warm N,N-dimethylformamide five times per day for 2 days to remove any unreacted starting materials from the pores. Then the samples were soaked in warm acetonitrile (40 °C) over a period of 5 days, replenishing the acetonitrile 4 times per day. Finally, the wet samples were transferred to 6 mm sample cells using a pipette and activated under dynamic vacuum at 80° C for 12 hours until the outgas rate was less than 2 mTorr/min. After evacuation, the samples were weighed to obtain their precise mass and the cells were transferred to the analysis port of the gas adsorption instrument.

Thermogravimetric analyses (TGA), were performed using a TA Instrument TGA 5500. An amount of approximately 10 mg of activated Fe-**tbb**-MOF-x was placed inside a quartz cap and heated up to at least 700° C under N_2 flow with a heating rate of 5 ° C/min.

Organic synthesis and ^{1}H NMR spectra

The tetratopic carboxylate ligand H_4PBPTA (4,4',4'',4'''-(1,4-phenylenebis(pyridine-4,2-6-triyl))-tetrabenzoic acid) was synthesized according to previous literature reports with slight modifications.[1] The precise synthetic procedure is described below (Scheme 1).

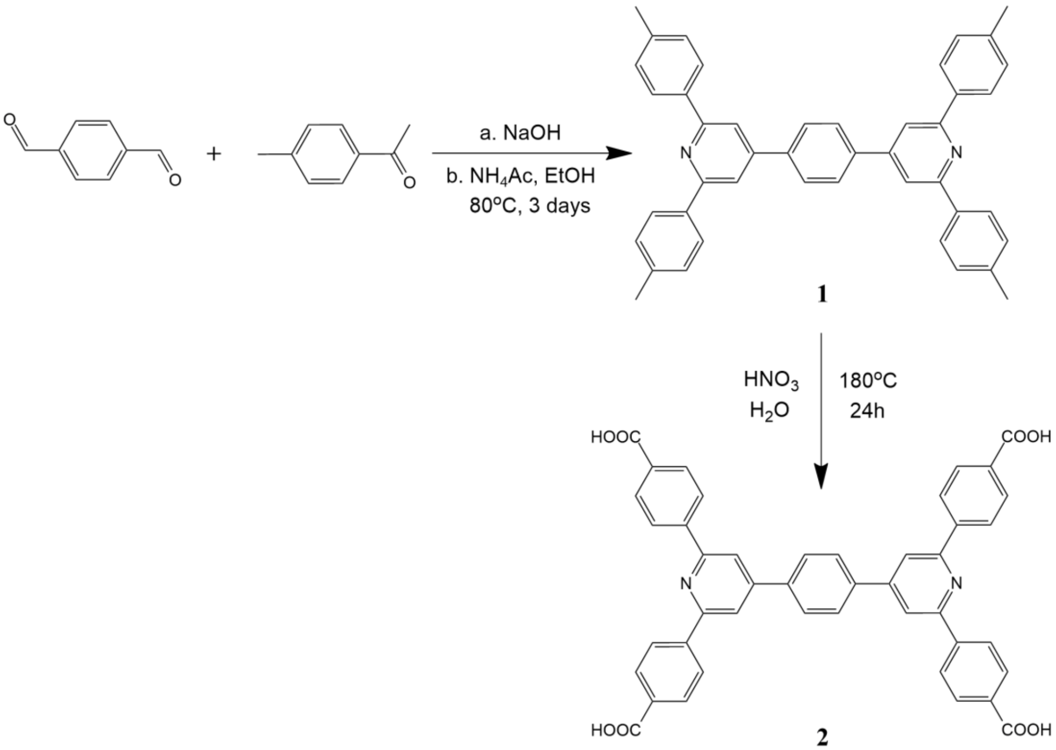

Scheme 1. Synthesis of 4,4',4'',4'''-(1,4-phenylenebis(pyridine-4,2-6-triyl))-tetrabenzoic acid (**H_4PBPTA**) ligand.

Compound 1. Using a mortar and a pestle, terephthalaldehyde (0.67 gr, 5.0 mmol), 4-methylacetophenone (3.36 gr, 25.0 mmol) and powder NaOH (1.23 gr, 30.8 mmol) were ground together for 60 minutes. The resulting yellow tacky solid was transferred to a 250-ml round bottom flask, containing a solution of ammonium acetate (7.58 gr, 98.3 mmol) in ethanol (150 ml). The reaction mixture was heated to reflux and stirred for 3 days. After that period, the mixture was cooled down to room temperature and the yellow precipitate was filtered, washed several times with water and finally recrystallized from toluene to afford a white crystalline product. Yield: 40%. ^{1}H NMR (500 MHz, $CDCl_3$): 8.13 (d, 8H), 7.90 (s, 8H), 7.34 (d, 8H), 2.45 (s, 12H).

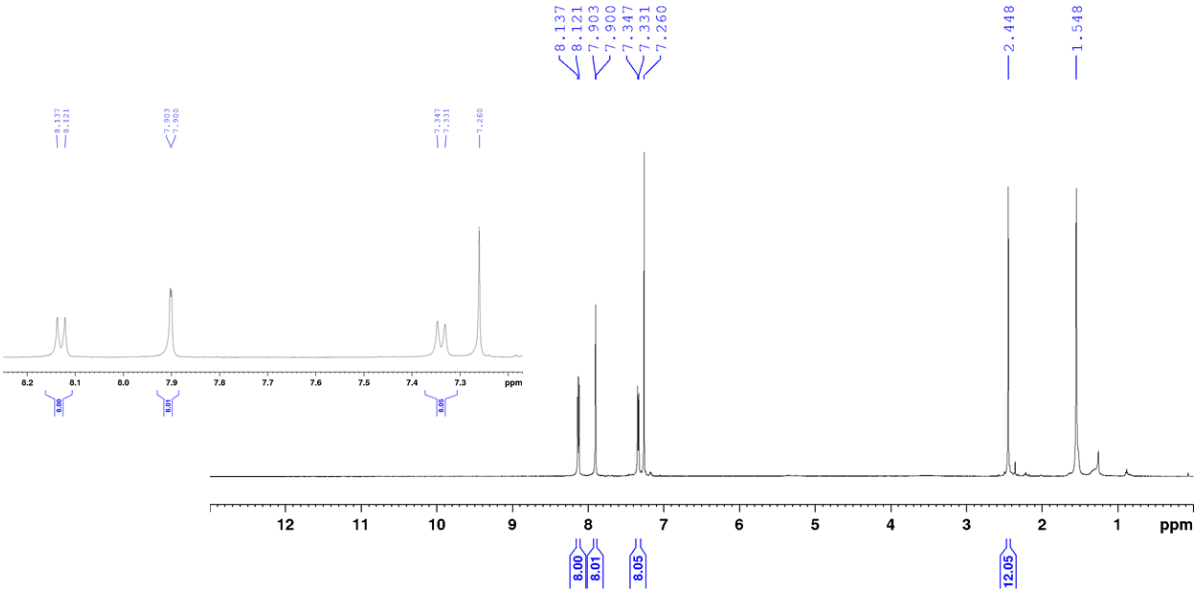

Figure S1. ^{1}H NMR spectrum of compound **1** in $CDCl_3$ (500 MHz).

Compound 2. To a 20ml Teflon-lined stainless steel autoclave, compound **1** (0.20 gr, 0.34 mmol) was mixed with 65% HNO_3 (1 ml) and H_2O (6 ml). The autoclave was sealed and heated to 180°C for 24h. Then, the reaction mixture was cooled down to room temperature and the resulting orange powder was collected through filtration, washed thoroughly with water and dried under vacuum. Yield: 45%. ^{1}H NMR (500 MHz, DMSO): 8.52 (d, 8H), 8.47 (s, 4H), 8.32 (s, 4H), 8.14 (d, 8H).

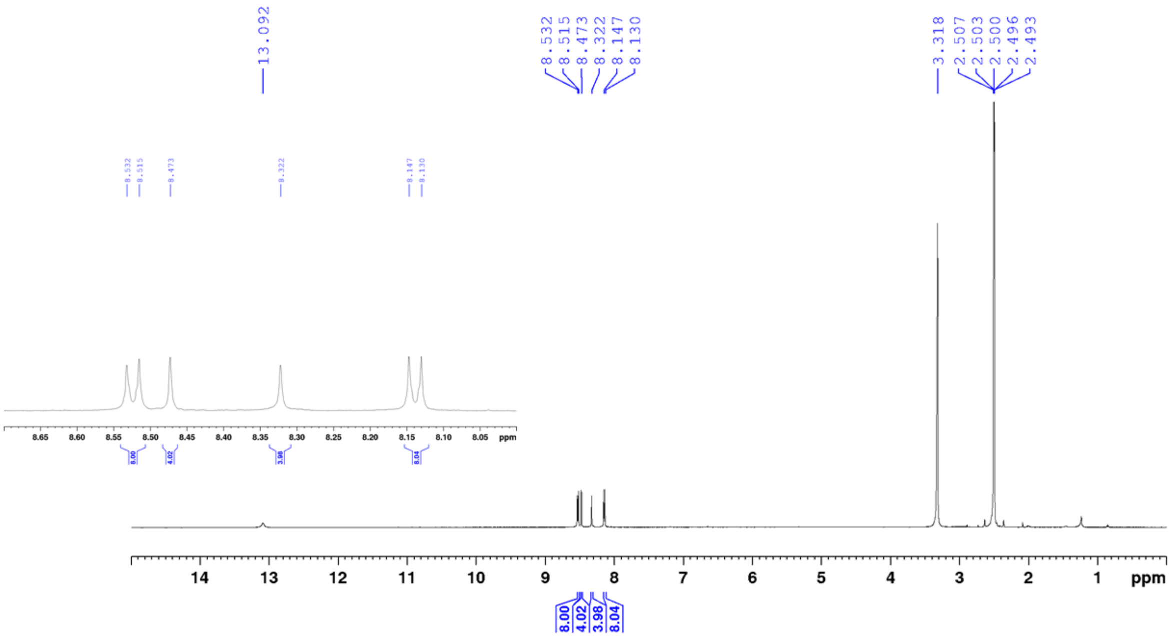

Figure S2. ^{1}H NMR spectrum of 4,4',4'',4'''-(1,4-phenylenebis(pyridine-4,2-6-triyl))-tetrabenzoic acid (compound **2**) in DMSO-d_6 (500 MHz).

The tritopic carboxylate ligand H_3TATB (4,4',4''-s-triazine-2,4,6-triyltribenzoic acid) was synthesized according to previous literature reports with slight modifications.[2] The precise synthetic procedure is described below (Scheme 2).

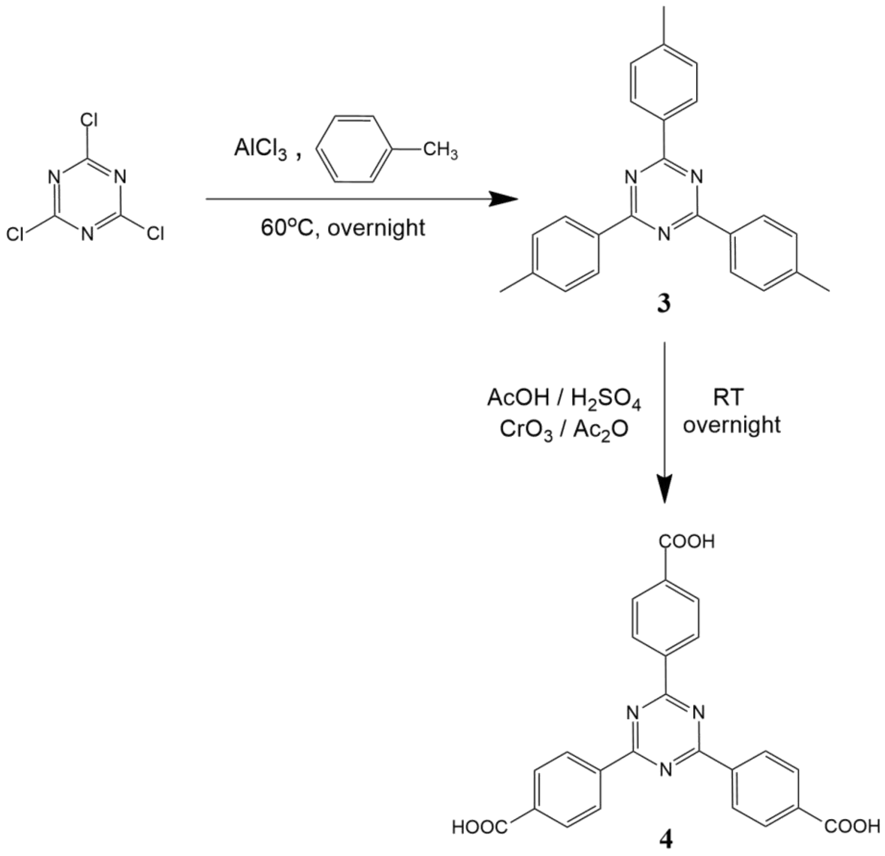

Scheme 2. Synthesis of 4,4',4''-s-triazine-2,4,6-triyltribenzoic acid (**H_3TATB**) ligand.

Compound 3. To a dried under Ar double neck round bottom flask, $AlCl_3$ (10.0 gr, 75.0 mmol) and dry toluene (25 ml) were added. The mixture was gently heated to 60°C and then cyanuric chloride (4.15 gr, 22.5 mmol) was added in 12 portions over the course of an hour. The resulting mixture was stirred overnight. After that period, the reaction was quenched with ice and stirred for an additional 30 minutes. The resulting mixture was extracted with $CHCl_3$ (2 x 25 ml). The combined organic layers were concentrated *in vacuo*. The crude product was recrystallized from toluene to afford a white needle-like crystalline solid. Yield: 58%. ^{1}H NMR (500 MHz, $CDCl_3$): 8.66 (d, 6H), 7.37 (d, 6H), 2.48 (s, 9H).

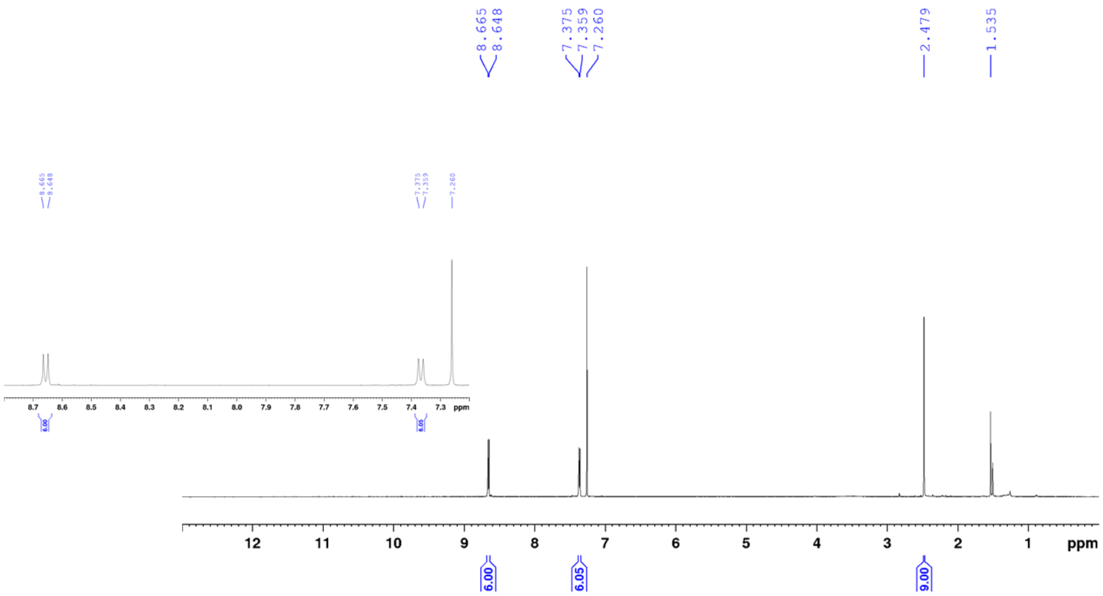

Figure S3. ^1H NMR spectrum of compound **3** in $CDCl_3$ (500 MHz).

Compound 4. To a dried under Ar double neck round bottom flask, compound **3** (0.20 gr, 0.57 mmol) was dissolved in acetic acid (12 ml) and H_2SO_4 (0.5 ml). The solution was cooled using an ice bath and chromium oxide (0.45 gr) and acetic anhydride (0.7 ml) were slowly added to the flask. The resulting dark-green reaction mixture was warmed to room temperature and stirred overnight. After that period, the reaction was quenched with ice, stirred for an additional 60 minutes and finally filtered. The off-white solid was washed thoroughly with water and then dissolved in an aqueous solution of NaOH (~10 ml, 2 M). Residues from unreacted starting material were removed by filtration and the resulting solution was acidified with an aqueous solution of HCl (until pH<2) to produce a white precipitate which was filtered, washed with water and dried under vacuum. Yield: 84%. ^1H NMR (500 MHz, DMSO): 8.85 (d, 6H), 8.21 (d, 6H).

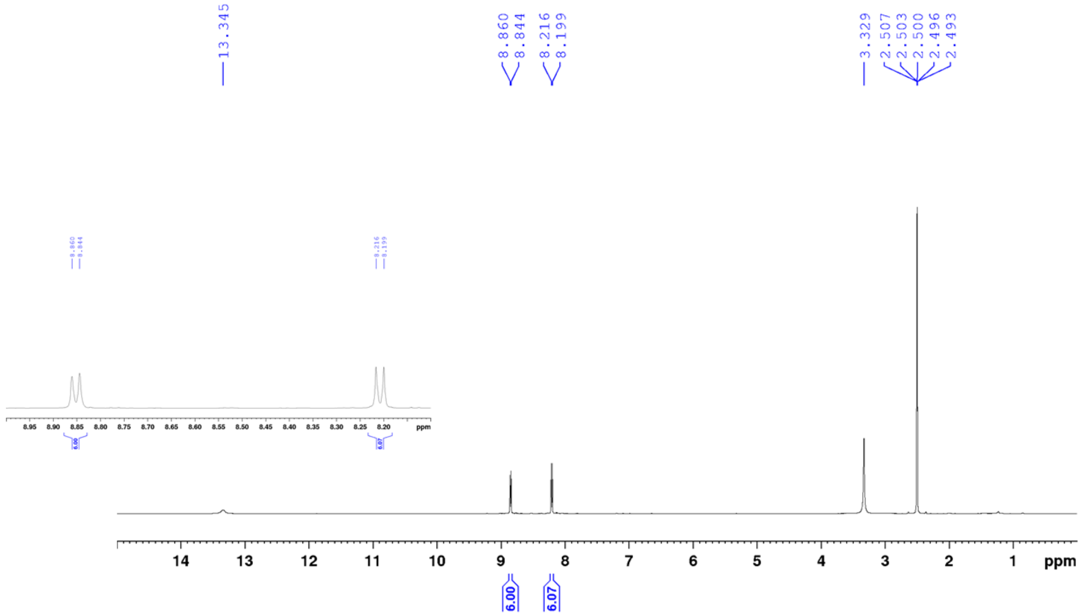

Figure S4. ^1H NMR spectrum of 4,4',4''-s-triazine-2,4,6-triyltribenzoic acid (compound **4**) in DMSO-d_6 (500 MHz).

The tritopic carboxylate ligand H_3PTB (4,4',4''-(Pyridine-2,4,6-triyl)tribenzoic acid) was synthesized according to previous literature reports with slight modifications.[3] The precise synthetic procedure is described below (Scheme 3).

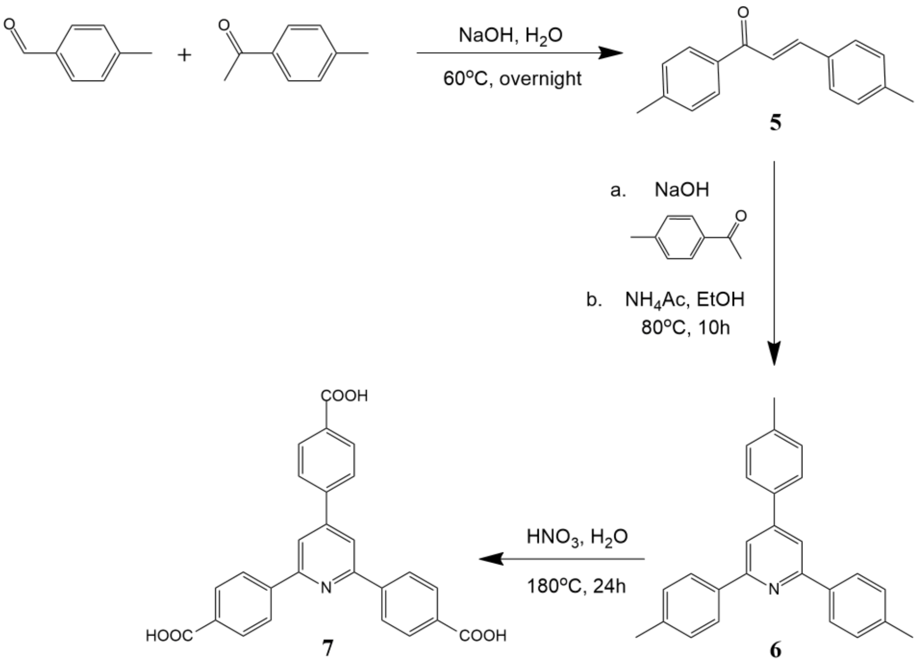

Scheme 3. Synthesis of 4,4',4''-(Pyridine-2,4,6-triyl)tribenzoic acid (**H₃PTB**) ligand.

Compound 5. To a 50 ml round bottom flask, 4-methylacetophenone (0.57 gr, 4.25 mmol), p-tolualdehyde (0.50 gr, 4.16 mmol) and an aqueous solution of NaOH 0.5 N (15 ml) were added. The mixture was vigorously stirred at room temperature for 60 minutes and then heated to 60°C overnight. After that period, the mixture was cooled down to room temperature and the yellow precipitate was filtered, washed several times with water and dried under vacuum. Yield: 87%. ^{1}H NMR (500 MHz, $CDCl_3$): 7.93 (d, 2H), 7.78 (d, 1H), 7.54 (d, 2H), 7.49 (d, 1H), 7.30 (d, 2H), 7.23 (d, 2H), 2.44 (s, 3H), 2.40 (s, 3H).

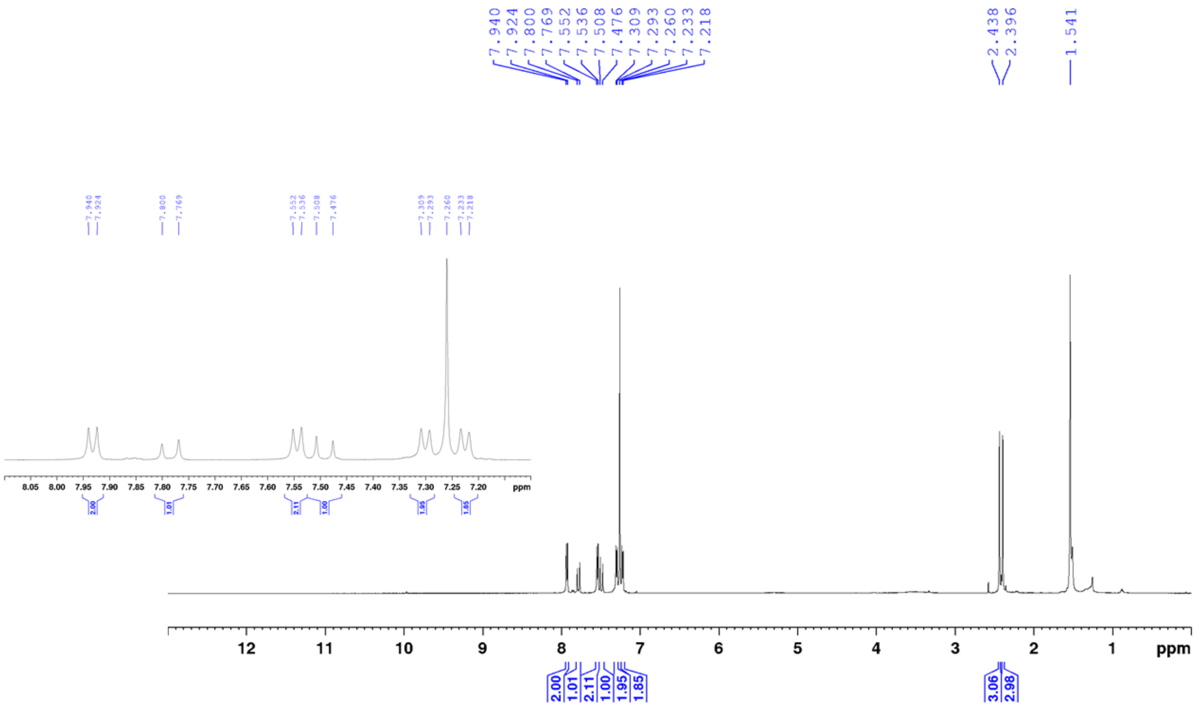

Figure S5. ^{1}H NMR spectrum of compound **5** in $CDCl_3$ (500 MHz).

Compound 6. Using a mortar and a pestle, compound **5** (1.45 gr, 6.14 mmol), 4-methylacetophenone (0.830 gr, 6.19 mmol) and powder NaOH (0.970 gr, 24.3 mmol) were ground together for 2 hours. The resulting yellow tacky solid was transferred to a 250-ml round bottom flask, containing a solution of ammonium acetate (7.00 gr, 90.8 mmol) in ethanol (80 ml). The reaction mixture was heated to reflux and stirred overnight. After that period, the mixture was cooled down to room temperature, filtered and washed several times with EtOH and the filtrate was concentrated *in vacuo*. The crude residue was purified by flash column chromatography (silica gel, petroleum ether : EtOAc = 90:1). Yield: 35%. ^{1}H NMR (500 MHz, $CDCl_3$): 8.10 (d, 4H), 7.83 (s, 2H), 7.65 (d, 2H), 7.32 (m, 6H), 2.44 (s, 3H), 2.43 (s, 6H).

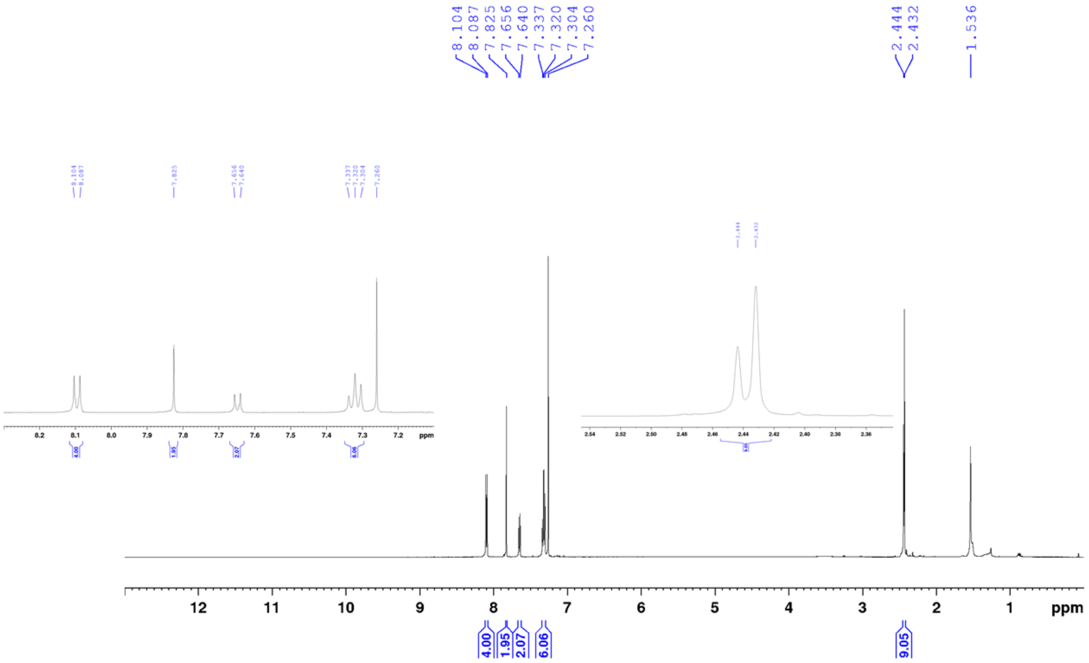

Figure S6. ^{1}H NMR spectrum of compound **6** in $CDCl_3$ (500 MHz).

Compound 7. To a 20ml Teflon-lined stainless steel autoclave, compound **6** (0.10 gr, 0.29 mmol) was mixed with 65% HNO_3 (0.5 ml) and H_2O (3 ml). The autoclave was sealed and heated to 180°C for 24h. Then, the reaction mixture was cooled down to room temperature and the resulting orange powder was collected through filtration, washed thoroughly with water and dried under vacuum. Yield: 65%. ^{1}H NMR (500 MHz, DMSO): 8.50 (d, 4H), 8.42 (s, 2H), 8.23 (d, 2H), 8.12 (d, 6H).

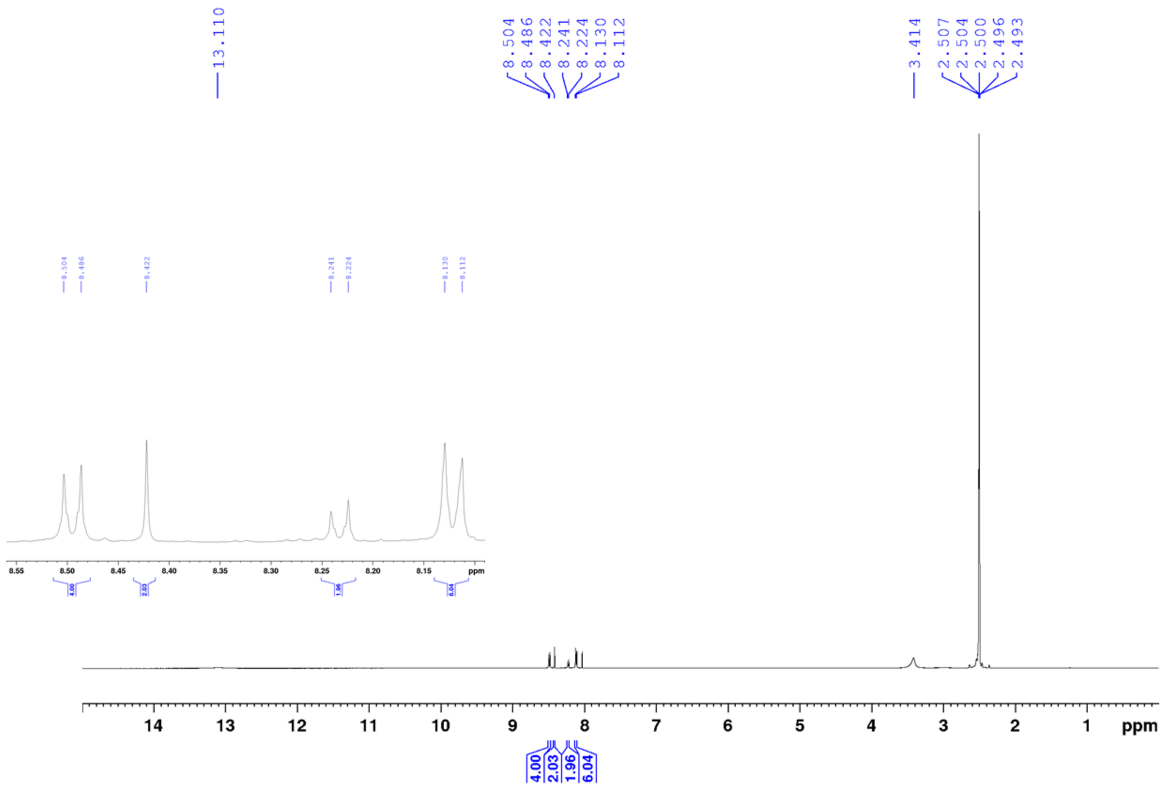

Figure S7. ^{1}H NMR spectrum of 4,4',4''-(Pyridine-2,4,6-triyl)tribenzoic acid (compound **7**) in DMSO-d_6 (500 MHz).

The tritopic carboxylate ligand H_3BTB (4,4',4''-benzene-1,3,5-triyl-tribenzoate) was synthesized according to previous literature reports with slight modifications.[4] The precise synthetic procedure is described below (Scheme 4).

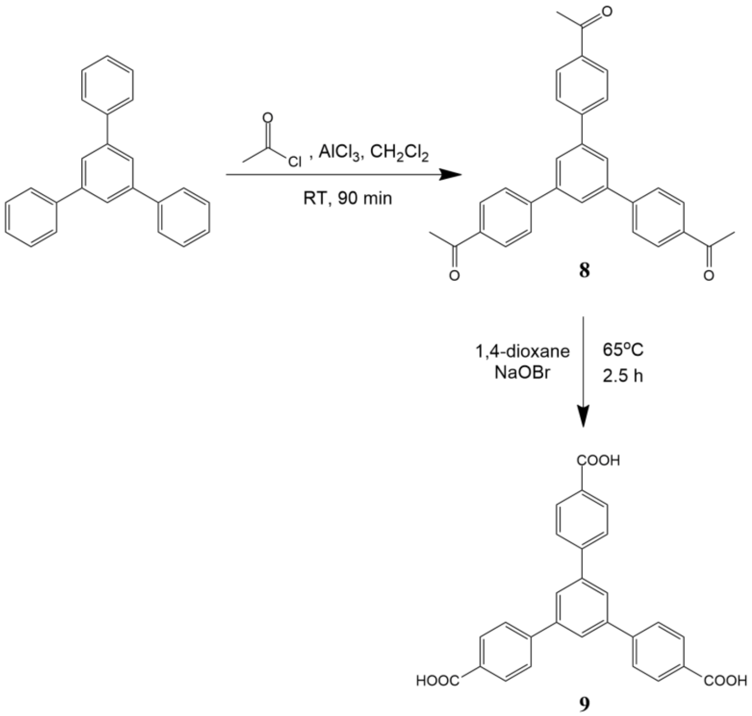

Scheme 4. Synthesis of 4,4',4''-benzene-1,3,5-triyl-tribenzoate (**H_3BTB**) ligand.

Compound 8. To a dried under Ar double neck round bottom flask, acetyl chloride (25 ml, 0.35 mmol) and $AlCl_3$ (5 gr, 37.5 mmol) were added. The content of the flask was cooled and retained at 0°C for 15min. After that period, a solution of 1,3,5 triphenyl benzene (1.5 gr, 4.9 mmol) dissolved in 20 ml CH_2Cl_2 was added to the flask. The mixture was stirred under argon at 0°C for 15min and then at 25°C for 90min. During that time, the mixture's color turned into deep red. The mixture was transferred in a flask which contained ~ 100ml of ice. The solution, which became yellow, was extracted with CH_2Cl_2 (x3). The organic layers were combined and washed with saturated aqueous solution of $NaHCO_3$ and then dried over $MgSO_4$, filtered and concentrated under reduced pressure. Yield: 87%. ^1H NMR (500 MHz, $CDCl_3$): 8.09 (d, 6H), 7.87 (s, 3H), 7.80 (d, 6H), 2.67 (s, 9H).

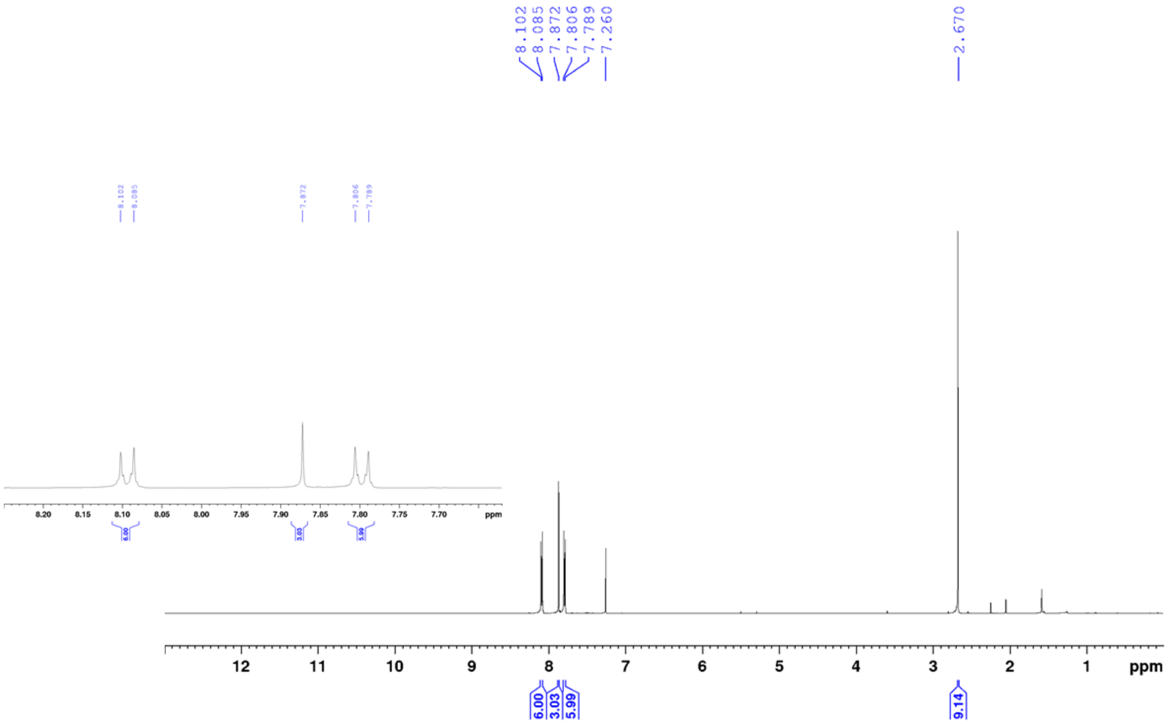

Figure S8. ^1H NMR spectrum of compound **8** in $CDCl_3$ (500 MHz).

Compound 9. Compound **8** (0.5 gr, 1.16 mmol) was dissolved in 1,4-dioxane (25 ml). The solution was placed in a 100ml round bottom flask. At the same time, NaOH (1.8 gr, 45 mmol) was dissolved in H_2O (12 ml) in a 50 ml beaker. The solution in the beaker was cooled at 0°C using an ice bath and then Br_2 (0.9 ml, 17.6 mmol) was added. The new solution was stirred at 0°C for 15min. Then, the solution was added gently to the round bottom flask and the mixture was heated at 65°C for 2.5h. After completion of the reaction, the mixture was cooled at room temperature and $Na_2S_2O_3 \cdot 5H_2O$ (0.3 gr) and c. HCl (4 ml) were added. The resulting solid was filtered, washed with H_2O and dried under vacuum. Yield: 75%.
^1H NMR (500 MHz, DMSO): 8.10 (s, 3H), 8.07 (s, 12H).

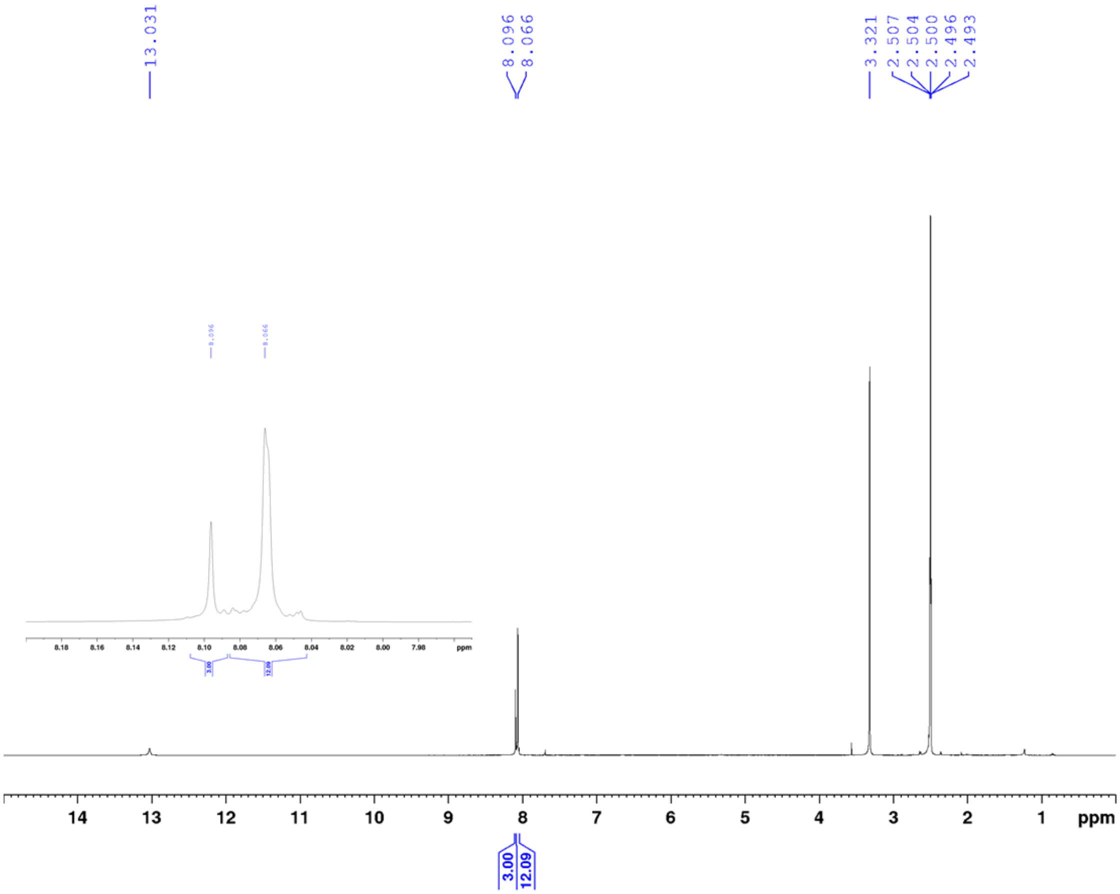

Figure S9. ^{1}H NMR spectrum of 4,4',4''-benzene-1,3,5-triyl-tribenzoate (compound **9**) in DMSO-d_6 (500 MHz).

MOFs syntheses

Synthesis of Fe-**tbb**-MOF-1 microcrystalline. $Fe(NO_3)_3\cdot 9H_2O$ (10.1 mg ,0.025 mmol), H_3PTB (1.3 mg, 0.003 mmol), H_4PBPTA (6.4 mg, 0.009 mmol), acetic acid (0.25 ml) and DMF (2.5 ml) were combined in a 20 ml scintillation vial. The vial was sealed and placed in an isothermal oven at 135°C for 3 days, after which orange microcrystalline material was formed. (26% yield based on H_4PBPTA).

Synthesis of Fe-**tbb**-MOF-1 single crystals. $Fe(NO_3)_3\cdot 9H_2O$ (18.0 mg ,0.045 mmol), H_3PTB (1.0 mg, 0.0023 mmol), H_4PBPTA (9.3 mg, 0.013 mmol), acetic acid (0.25 ml) and DMF (1.2 ml) were combined in a 20 ml scintillation vial. The vial was sealed and placed in an isothermal oven at 135°C for 2 days, after which yellow, hexagonal rod crystals were formed.

Synthesis of Fe-**tbb**-MOF-2. $Fe(NO_3)_3\cdot 9H_2O$ (10.1 mg ,0.025 mmol), H_3TATB (1.3 mg, 0.003 mmol), H_4PBPTA (6.4 mg, 0.009 mmol), acetic acid (0.25 ml) and DMF (2 ml) were combined in a 20 ml scintillation vial. The vial was sealed and placed in an isothermal oven at 135°C for 4 days, after which orange microcrystalline material was formed. (20% yield based on H_4PBPTA).

Synthesis of Fe-**tbb**-MOF-3. $Fe(NO_3)_3\cdot 9H_2O$ (10.1 mg ,0.025 mmol), H_3BTB (2.6 mg, 0.006 mmol), H_4PBPTA (6.4 mg, 0.009 mmol), acetic acid (0.25 ml) and DMF (2 ml) were combined in a 20 ml scintillation vial. The vial was sealed and placed in an isothermal oven at 80°C for 60 minutes and then at 135°C for 5 days, after which orange microcrystalline material was formed. (23% yield based on H_4PBPTA).

Synthesis of Fe-**tbb**-MOF-3 single crystals. $Fe(NO_3)_3$ $9H_2O$ (10.1 mg ,0.025 mmol), H_3BTB (2.6 mg, 0.006 mmol), H_4PBPTA (6.4 mg, 0.009 mmol), acetic acid (0.25 ml) and DMF (2.5 ml) were combined in a 20 ml scintillation vial. The vial was sealed and placed in an isothermal oven at 80°C for 3 hours and then at 135°C for 2 days, after which orange, hexagonal crystals were formed.

Scanning electron microscopy (SEM) images

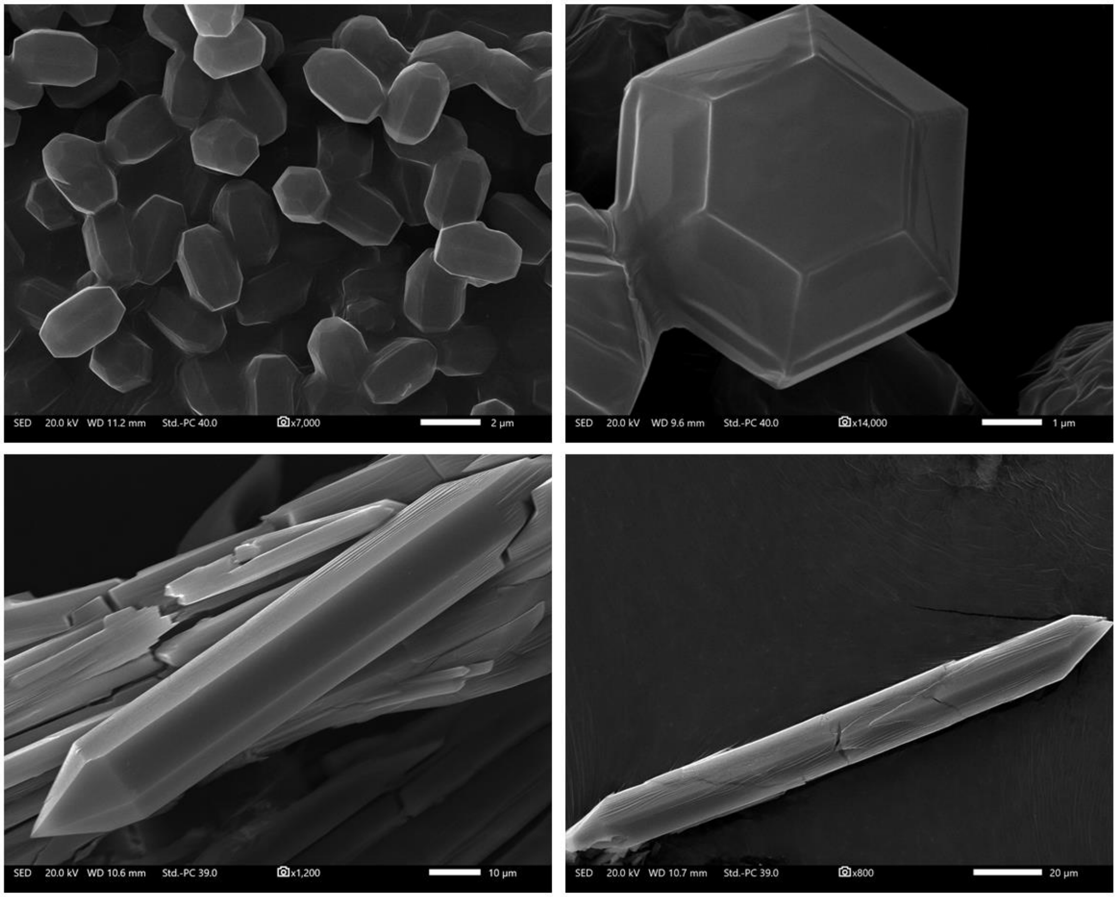

Figure S10. Representative scanning electron microscopy (SEM) images of microcrystals (top) and large single crystals (bottom) of Fe-**tbb**-MOF-1.

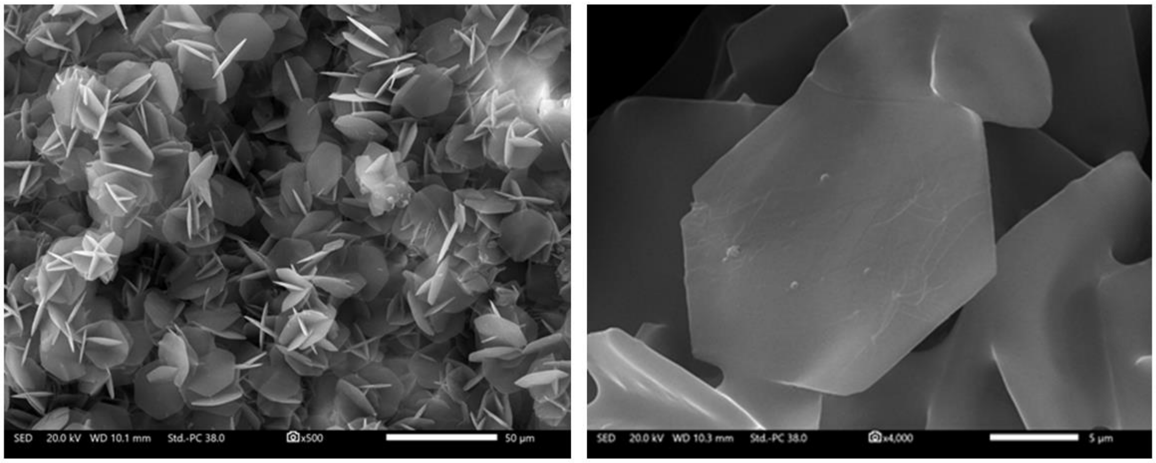

Figure S11. Representative SEM images of as-made Fe-**tbb**-MOF-2.

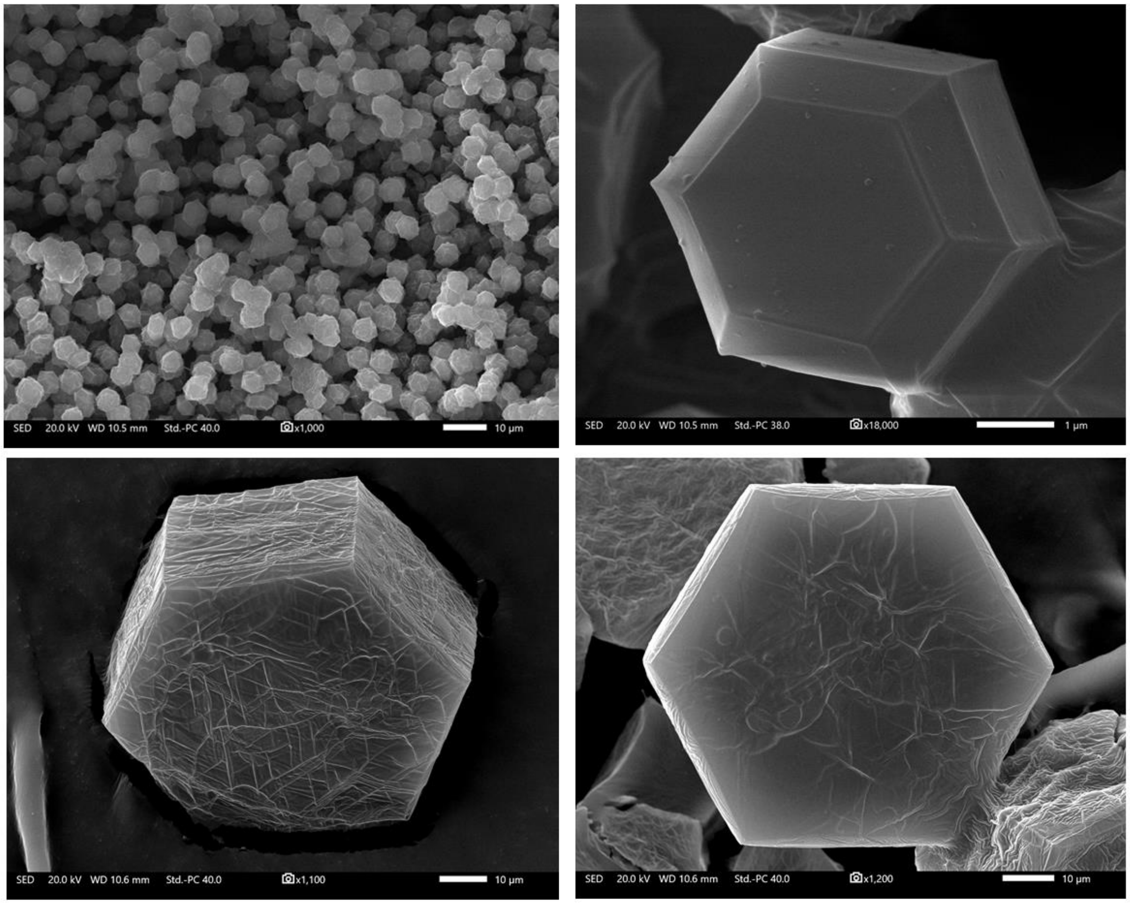

Figure S12. Representative SEM images of microcrystals (top) and large single crystals (bottom) of as-made Fe-**tbb**-MOF-3.

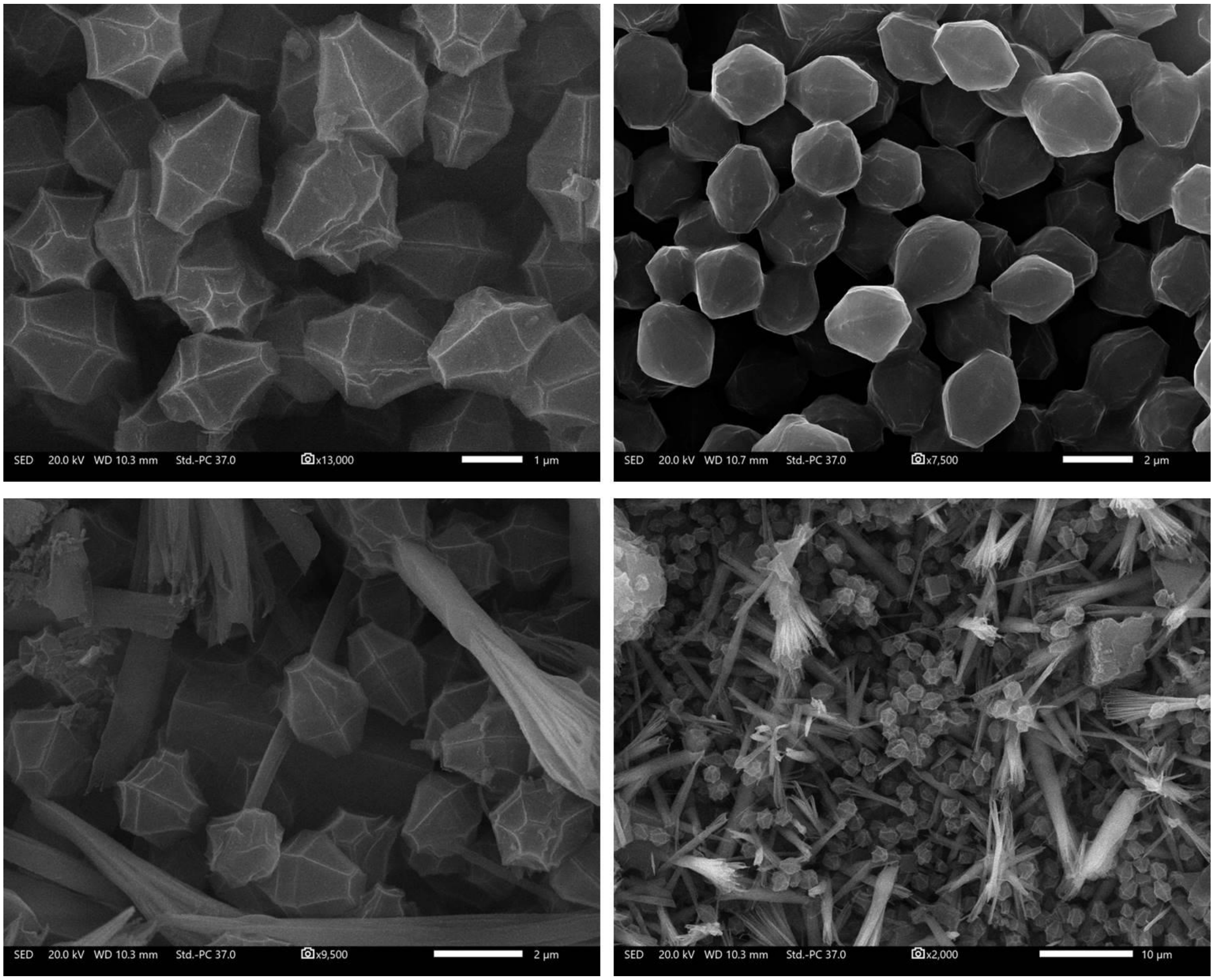

Figure S13. Representative SEM images of the hexagonal crystals of Al-**tbb**-MOF-1 obtained as a mixed phase with Al-**soc**-MOF as revealed by PXRD.

Single crystal X-ray crystallography and additional structural figures

In-house SCXRD measurement

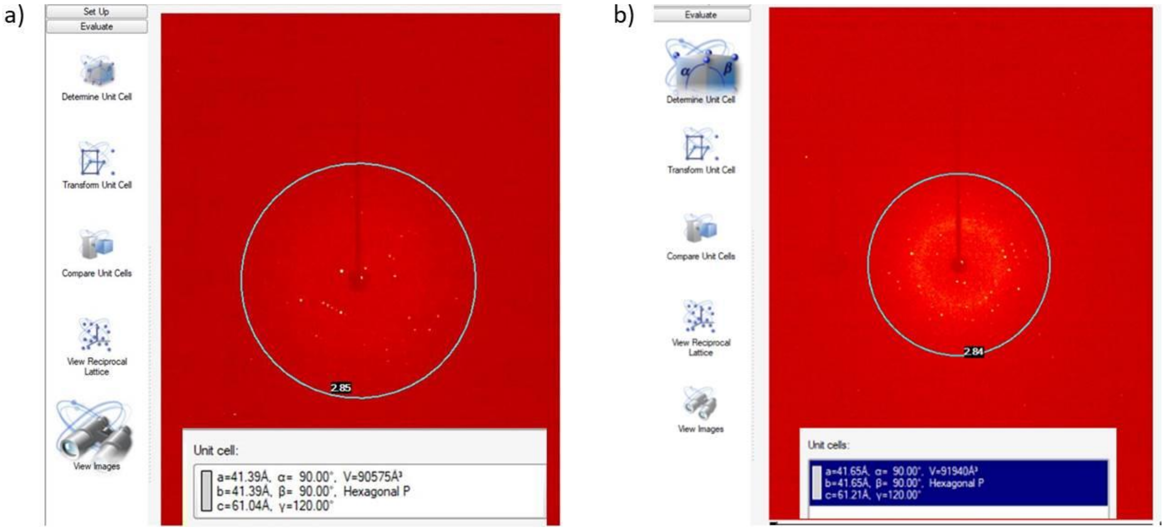

Figure S14. Representative X-ray diffraction frames from a) Fe-**tbb**-MOF-1 and b) Fe-**tbb**-MOF-3 single crystals using an in-house machine (Bruker, D8 Venture, CuKα, microfocus source IμS 3.0 – incoatec), showing the limited diffraction resolution due to the large unit cell and low skeletal density. The obtained unit cells are shown in inset at the bottom.

Synchrotron SCXRD measurement of Fe-**tbb**-MOF-1

A specimen of $C_{444}H_{240}Fe_{21}N_{24}O_{112}$ was used for the X-ray crystallographic analysis. The X-ray intensity data were measured (λ = 0.72932 Å). The integration of the data using a hexagonal unit cell yielded a total of 213464 reflections to a maximum θ angle of 27.51° (0.79 Å resolution), of which 53899 were independent (average redundancy 4.025, completeness = 87%, R_{int}= 7.66%, R_{sig} = 9.43%) and 24938 (46.29%) were greater than 2σ(F^2). The final cell constants of a = 42.09810(10) Å, b = 42.09810(10) Å, c = 61.5509(3) Å, volume = 94469.1(6) Å^3, are based upon the refinement of the XYZ-centroids of reflections above 20 σ(I). The final anisotropic full-matrix least-squares refinement on F^2 with 822 variables converged at R1 = 3.36%, for the observed data and wR2 = 7.12% for all data. The goodness-of-fit was 0.656. The largest peak in the final difference electron density synthesis was 0.10 e^-/Å^3 and the largest hole was -0.16 e^-/Å^3 with an RMS deviation of 0.013 e^-/Å^3. On the basis of the final model, the calculated density was 0.312 g/cm^3 and F(000), 9028 e^-. The crystallographic data of Fe-**tbb**-MOF-1 have been deposited at the Cambridge Crystallographic Data Centre as supplementary publication no. CCDC 2307369.

Table S1 Crystal data and structure refinement for Fe-tbb-MOF-1.

Identification code	Fe-**tbb**-MOF-1
Empirical formula	$C_{444}H_{240}Fe_{21}N_{24}O_{112}$
Formula weight	8875.44
Temperature/K	200.15
Crystal system	hexagonal
Space group	P-62c
a/Å	42.09810(10)
b/Å	42.09810(10)
c/Å	61.5509(3)
α/°	90
β/°	90
γ/°	120
Volume/Å^3	94469.1(6)
Z	2
ρ_{calc}g/cm^3	0.312
μ/mm^{-1}	0.186
F(000)	9028.0
Crystal size/mm^3	0.09 × 0.025 × 0.015
Radiation	synchrotron (λ = 0.72932)
2Θ range for data collection/°	1.776 to 55.022
Index ranges	-23 ≤ h ≤ 44, -48 ≤ k ≤ 43, -70 ≤ l ≤ 67
Reflections collected	213464
Independent reflections	53899 [R_{int} = 0.0766, R_{sigma} = 0.0943]
Data/restraints/parameters	53899/777/822
Goodness-of-fit on F^2	0.656
Final R indexes [I>=2σ (I)]	R_1 = 0.0336, wR_2 = 0.0672
Final R indexes [all data]	R_1 = 0.0640, wR_2 = 0.0712
Largest diff. peak/hole / e Å^{-3}	0.10/-0.16
Flack parameter	0.053(7)

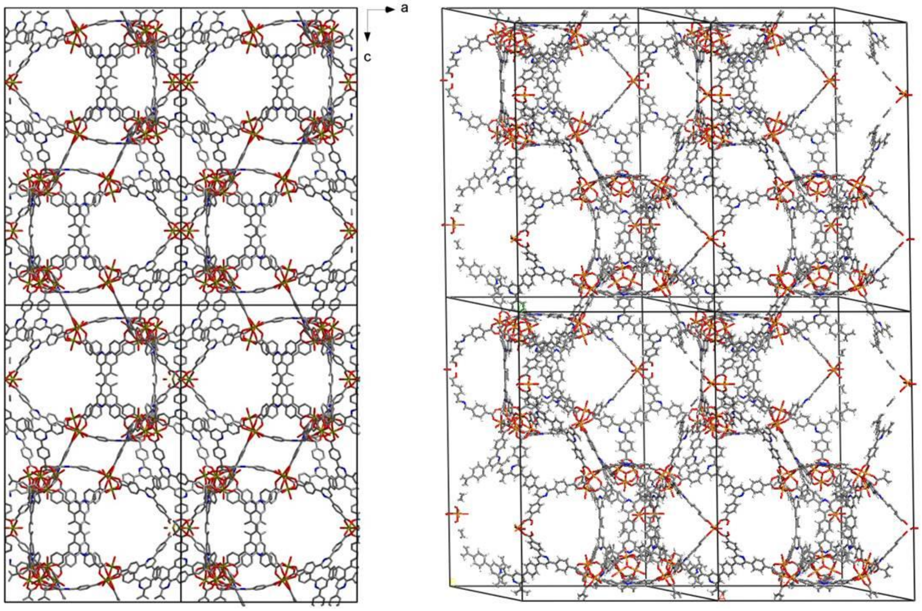

Figure S15. The structure of Fe-**ttb**-MOF-1 looking down the b-axis (left) and slightly off the ac-plane (right).

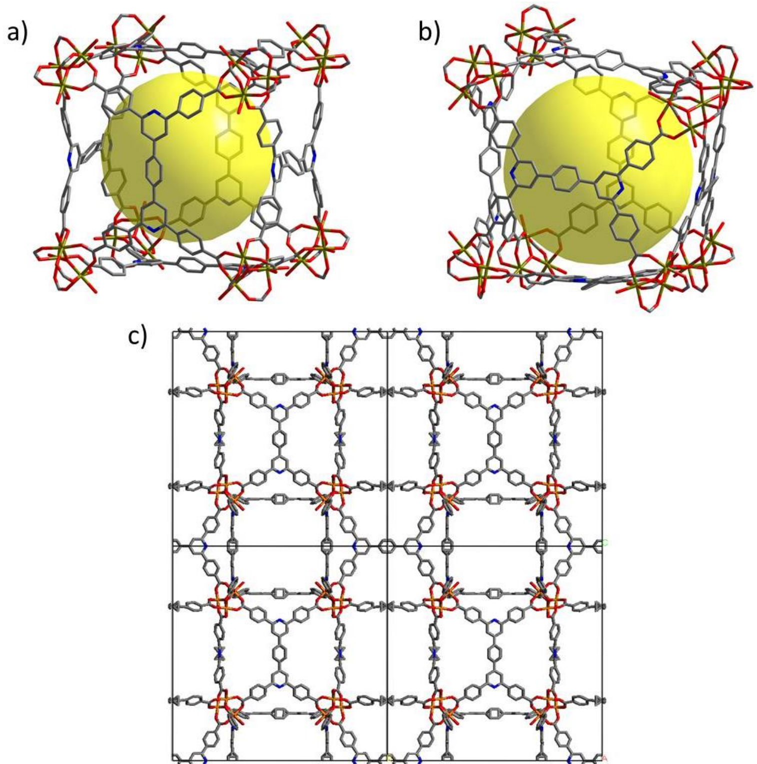

Figure S16. a) Regular **soc**-type cuboidal cages observed in Fe-pbpta (see ref. #36 in the manuscript) and b) distorted cuboidal cages in Fe-**tbb**-MOF-1. Both cages are made from eight $Fe_3(\mu_3\text{-}O)(\text{-}COO)_6$ clusters occupying the corners and six $PBPTA^{4-}$ ligands. As described in the manuscript, the origin of the distortion in the case of Fe-**tbb**-MOF-1, originates from the relative arrangement of PBPTA ligands in neighboring cuboidal faces. Yellow spheres denote the empty space. c) The structure of Fe-pbpta made of edge-shared regular **soc**-type, cuboidal cages.

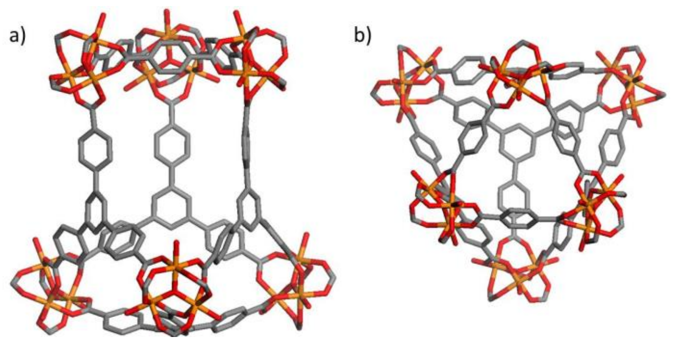

Figure S17. The mixed linker cage observed in MIL_142A made of six $Fe_3(\mu_3\text{-}O)(\text{-}COO)_6$ clusters, four 3-c BTB and three 2-c BDC ligands, in side (a) and top (b) view. The six $Fe_3(\mu_3\text{-}O)(\text{-}COO)_6$ clusters are arranged in staggered form, resulting in a super-octahedron (ref #46 in the manuscript).

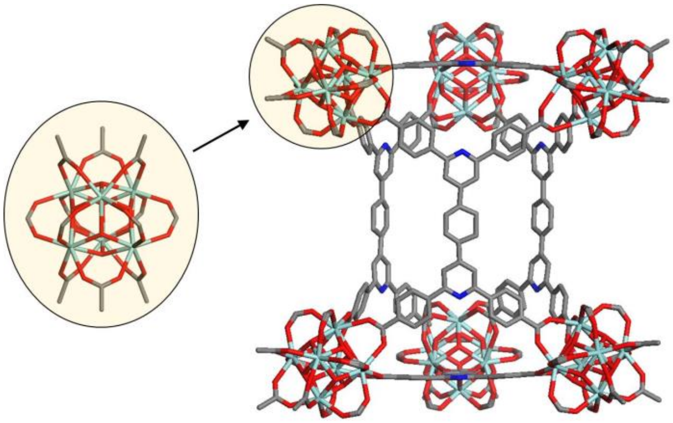

Figure S18. Hypothetical trigonal prismatic **tbb** made of six trigonal anti-prismatic $Zr_6O_4(OH)_4(\text{-}COO)_6$ clusters, in combination with two PTB (top and bottom) and three PBPTA (side) organic ligands.

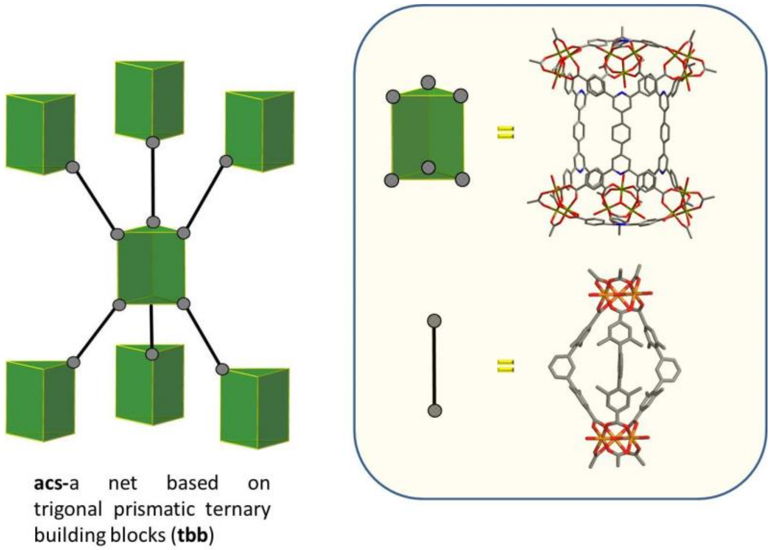

Figure S19. Hypothetical **acs** type MOF constructed from corner-shared **tbb's** using suitable angular dicarboxylate bridges.

Topological analysis considering the <u>4-c organic linker as a 4-connected building unit</u>

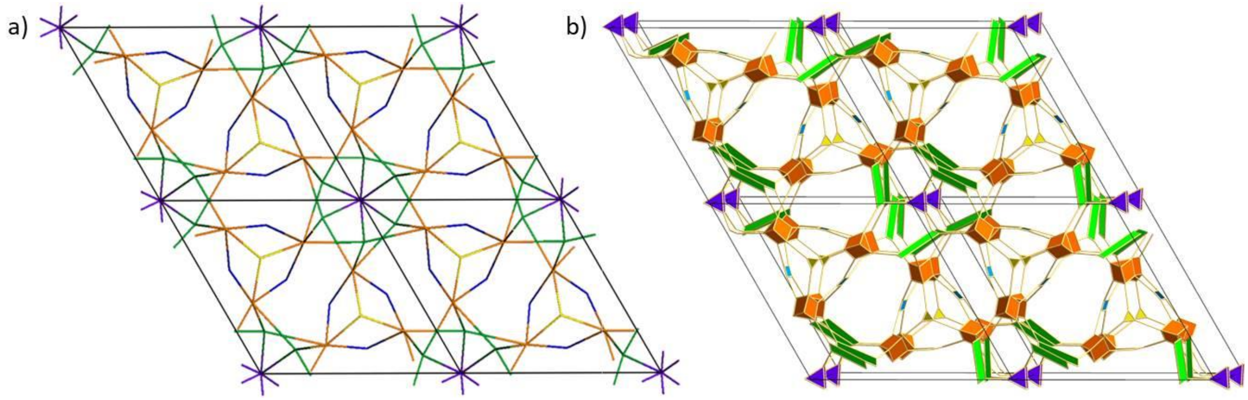

Figure S20. Topological analysis of Fe-**tbb**-MOF-1. a) The non-augmented **tbb** net is obtained by reducing the 6-c $Fe_3O(CO_2)_6$-**a** and $Fe_3O(CO_2)_6$-**b** clusters to 6-c orange and purple nodes respectively, the 4-c PBPTA-**a** and 4-c PBPTA-**b** linkers to 4-c **blue** and **green** nodes and the 3-c PTB ligand to a 3-c yellow node. (b) The augmented **tbb-a** net.

The topological analysis results are summarized below:
TD10=1634

Vertex symbols for selected sublattice

3-c yellow node Point symbol:{4^3}
Extended point symbol:[4.4.4]

4-c blue node Point symbol:{4^4.6^2}
Extended point symbol:[4.4.4.4.6(6).6(6)]

4-c green node Point symbol:{4^4.6^2}
Extended point symbol:[4.4.4.4.6(4).6(4)]

6-c orange node Point symbol:{4^6.6^4.8^5}
Extended point symbol:[4.4.4.4.4.4.6(2).6(2).6(2).6(2).8(8).8(8).8(8).8(8).8(8)]

6-c purple node Point symbol:{4^6.6^9}
Extended point symbol:[4.4.4.4.4.4.6(2).6(2).6(2).6(2).6(2).6(2).6(4).6(4).6(4)]

Point symbol for net: {4^3}2{4^4.6^2}9{4^6.6^4.8^5}6{4^6.6^9}
3,4,4,6,6-c net with stoichiometry (3-c)2(4-c)6(4-c)3(6-c)6(6-c); 5-nodal net

Topological analysis considering the 4-c organic linker as two interconnected 3-c building units

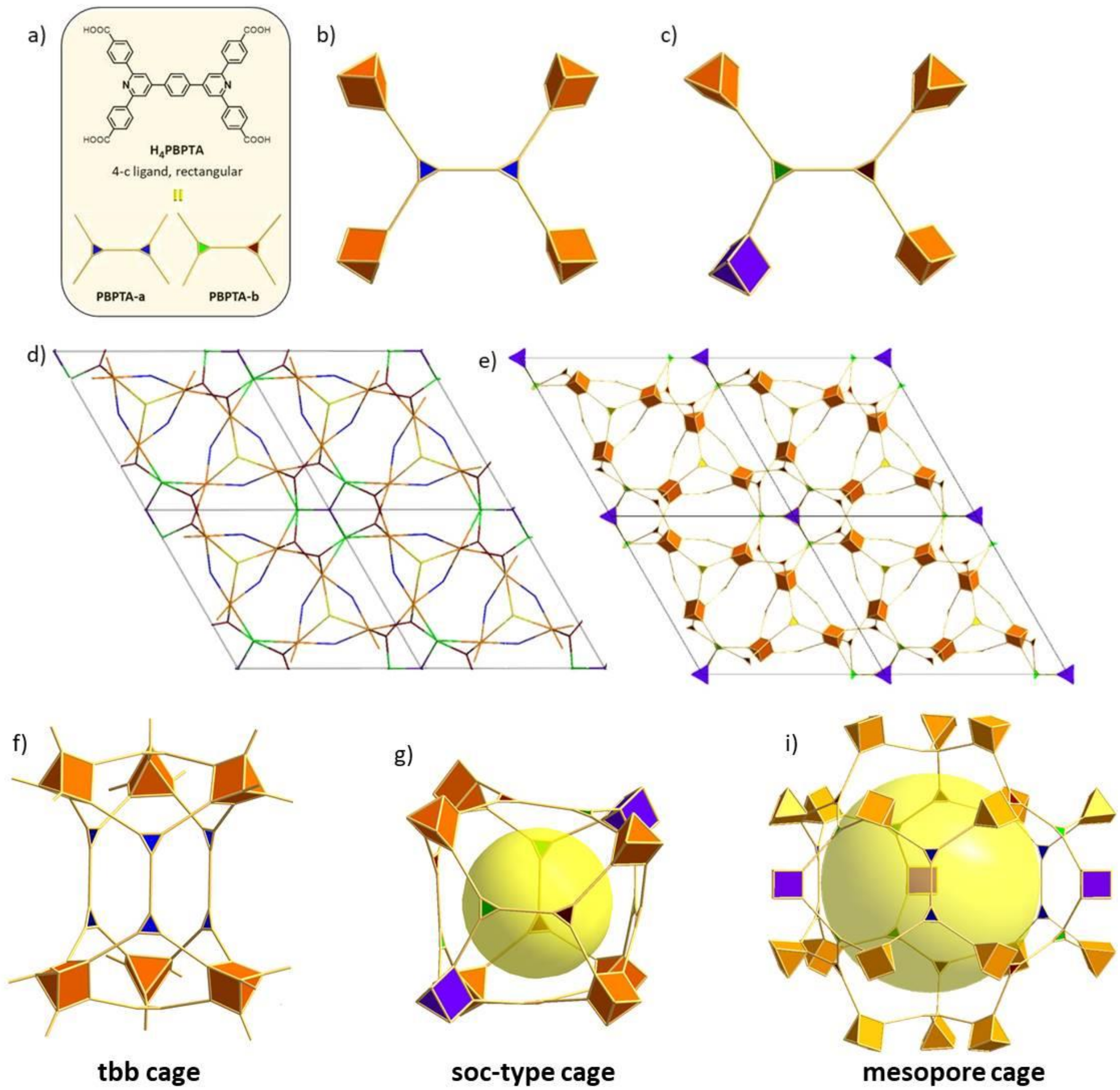

Figure S21. Topological analysis of Fe-**tbb**-MOF-1 where the 4-connected PBPTA linker is considered as two interconnected 3-c building units (a-c). d) The derived non-augmented **tft** and e) the augmented **tft-a** (3,3,3,3,6,6)-c 6-nodal net. The augmented **tbb**, **soc**-type and mesopore cages are shown in (f), (g) and (i), respectively. The simplified non-augmented **tft** net (d) is obtained by reducing the 6-c $Fe_3O(CO_2)_6$-**a** and $Fe_3O(CO_2)_6$-**b** clusters to 6-c orange and purple nodes respectively, the 3-c PTB ligand to a 3-c yellow node, the 4-c PBPTA-**a** to two blue interconnected 3-c triangular nodes (b), while the 4-c PBPTA-**b** to two interconnected distinct 3-c nodes, green and dark red, shown in (c). It is noted that the reduced 4-c PBPTA-**a** linker bridges four different orange nodes within the same **tbb** unit (b), while the reduced 4-c PBPTA-**b** linker bridges three orange 6-c nodes from three different **tbb** units and one purple 6-c node (c). For this reason, the topological analysis considering the 4-c PBPTA linkers as interconnected 3-c units, results in three different 3-c nodes, one blue, one green and one dark red. Adding to this the triangular 3-c yellow node from the reduction of the 3-c PTB ligand and the two 6-c orange and purle nodes from the reduction of the 6-c $Fe_3O(CO_2)_6$ clusters, the resulting 6-nodal **tft** net has a connectivity of (3,3,3,3,6,6)-c.

The topological analysis results are summarized below:
TD10=1045
Vertex symbols for selected sublattice

3-c yellow node Point symbol:{4^3}
Extended point symbol:[4.4.4]

3-c blue node Point symbol:{4.6^2}
Extended point symbol:[4.6.6]

3-c green node Point symbol:{5^2.6}
Extended point symbol:[5.5.6]

3-c dark red node Point symbol:{4.5.6}
Extended point symbol:[4.5.6]

6-c orange node Point symbol:{4^3.5.6^2.7^2.8^2.9^5}
Extended point symbol:[4.4.4.5.6.6(3).7.7.8.8.9(4).9(4).9(4).9(6).9(8)]

6-c purple node Point symbol:{5^6.7^3.8^6}
Extended point symbol:[5.5.5.5.5.5.7(2).7(2).7(2).8(2).8(2).8(2).8(2).8(2).8(2)]

Point symbol for net: {4.5.6}6{4.6^2}6{4^3.5.6^2.7^2.8^2.9^5}6{4^3}2{5^2.6}6{5^6.7^3.8^6}
3,3,3,3,6,6-c net with stoichiometry (3-c)2(3-c)6(3-c)6(3-c)6(6-c)6(6-c); 6-nodal net

Powder X-ray diffraction measurements

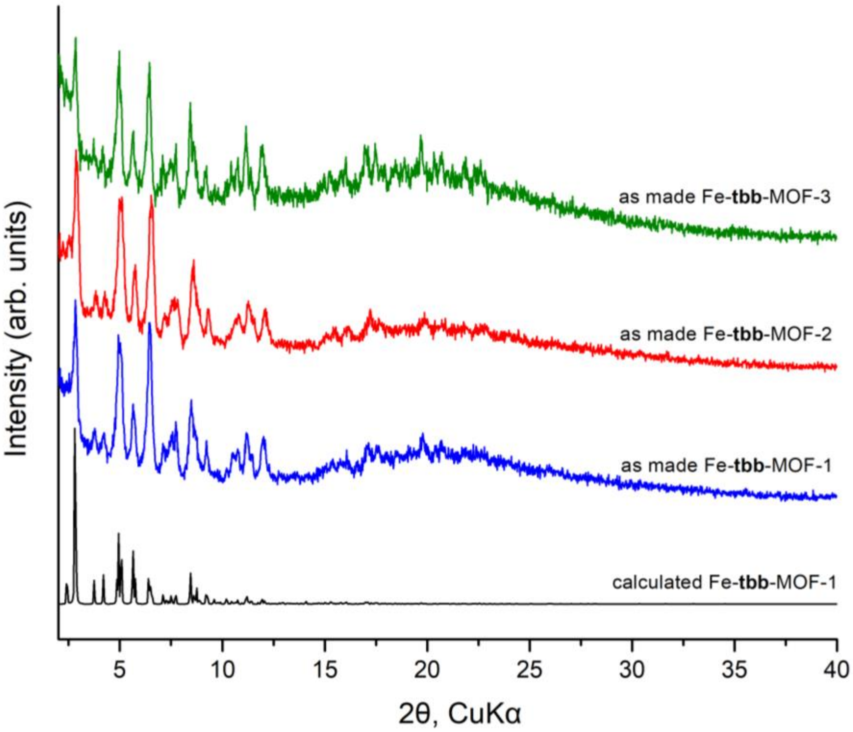

Figure S22. Experimental PXRD pattern of as-made Fe-**tbb**-MOF-1, Fe-**tbb**-MOF-2 and Fe-**tbb**-MOF-3 along with the calculated pattern obtained from the single crystal structure of Fe-**tbb**-MOF-1.

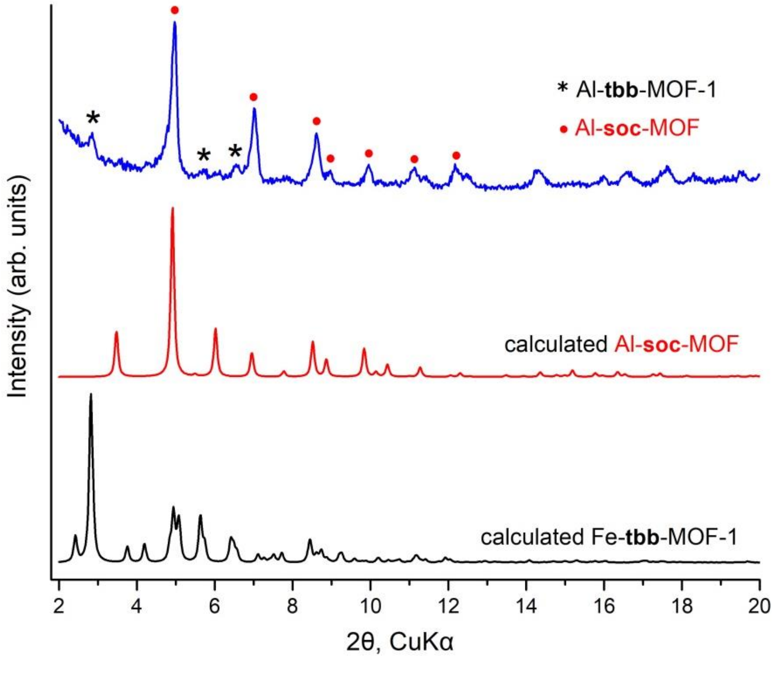

Figure S23. Experimental PXRD pattern (blue) of the material obtained from the reaction mixture $Al(NO_3)_3$/3-c/4-c, where mixed Al-**ttb**-MOF-1 and Al-**soc**-MOF phases were isolated. For comparison, the calculated PXRD pattern of Fe-**tbb**-MOF-1 and Al-**soc**-MOF are presented.

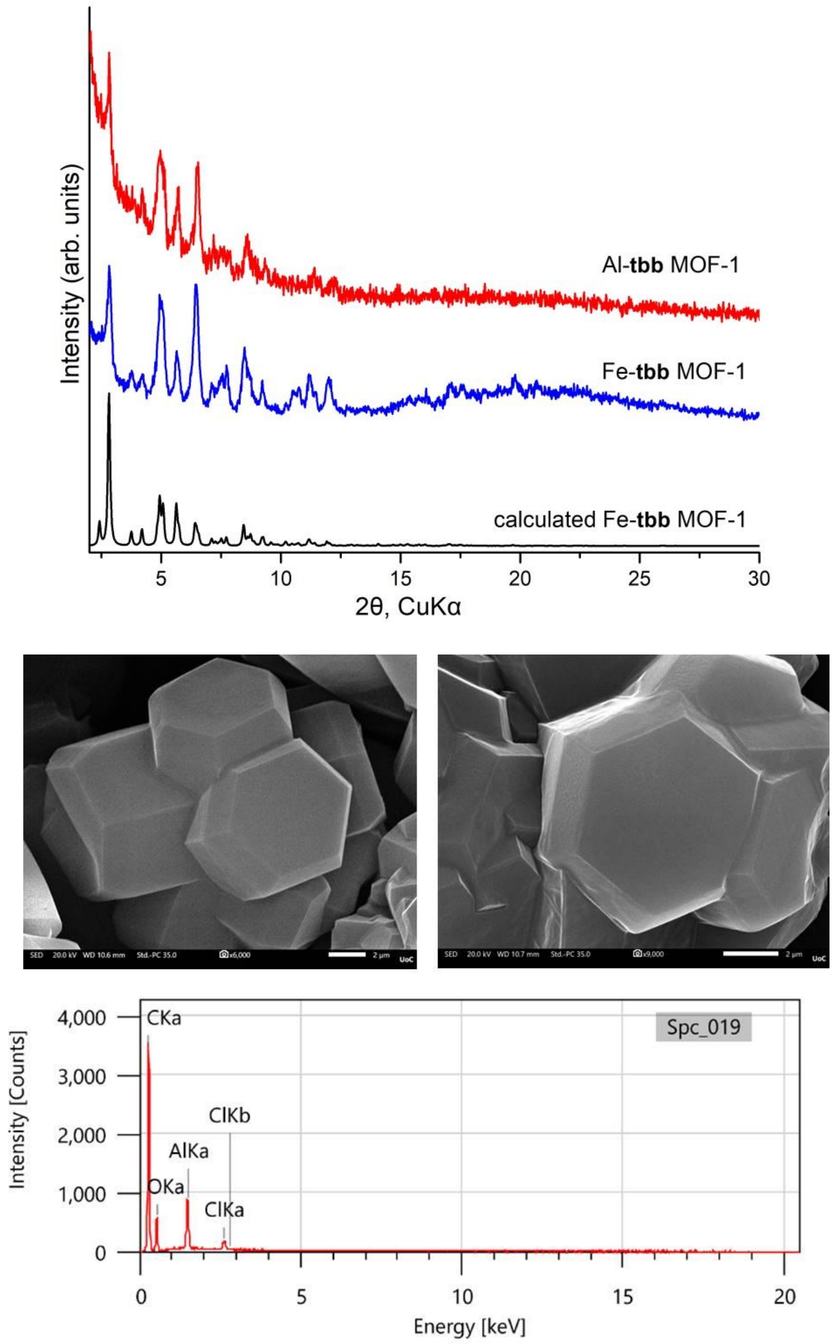

Figure S24. (Top) Experimental PXRD pattern of as-made Al-**tbb**-MOF-1 obtained by a post-synthetic metal exchange reaction from Fe-**tbb**-MOF-1, along with the calculated pattern from the single crystal structure of the latter. (Middle) Representative Al-**tbb**-MOF-1 crystals obtained after the post-synthetic metal exchange reaction. (Bottom) The complete metal exchange reaction was confirmed by SEM/EDS analysis, showing the absence of Fe. Details will be published elsewhere.

^{1}H NMR of acid digested samples

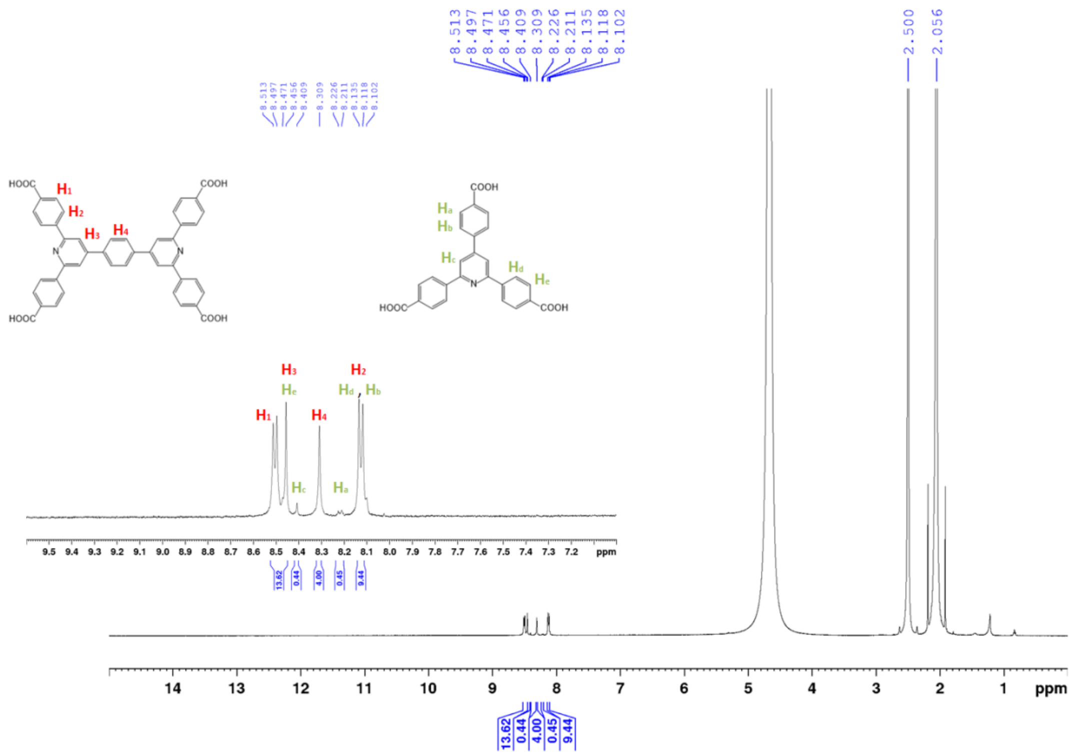

Figure S25. ^{1}H NMR spectrum of MeCN exchanged Fe-**tbb**-MOF-1 after digesting the sample in HCl/DMSO-d_6 solution.

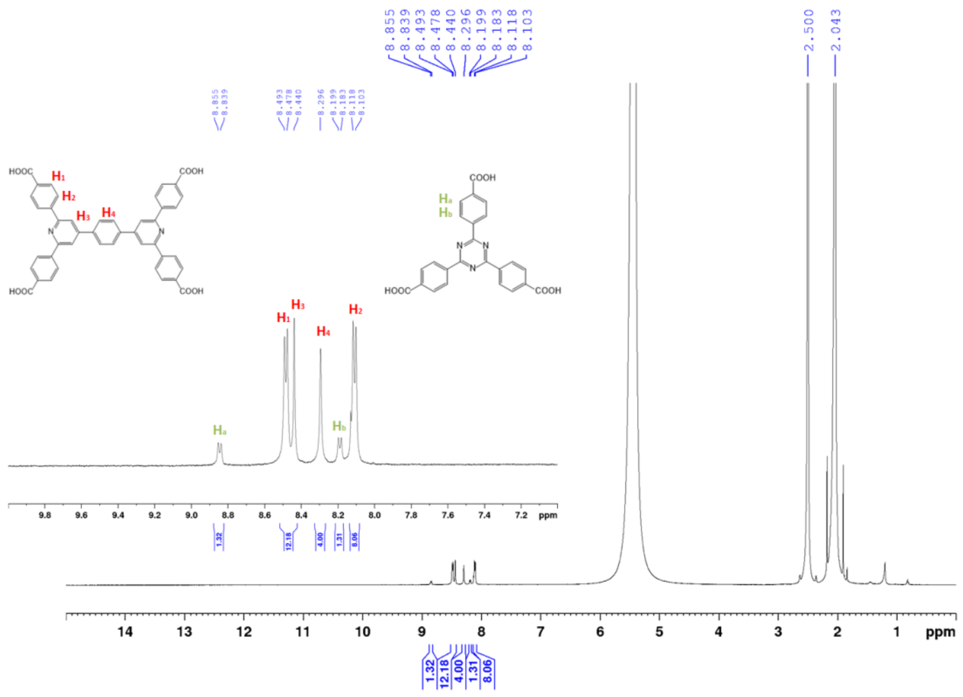

Figure S26. ^{1}H NMR spectrum of MeCN exchanged Fe-**tbb**-MOF-2 after digesting the sample in HCl/DMSO-d_6 solution.

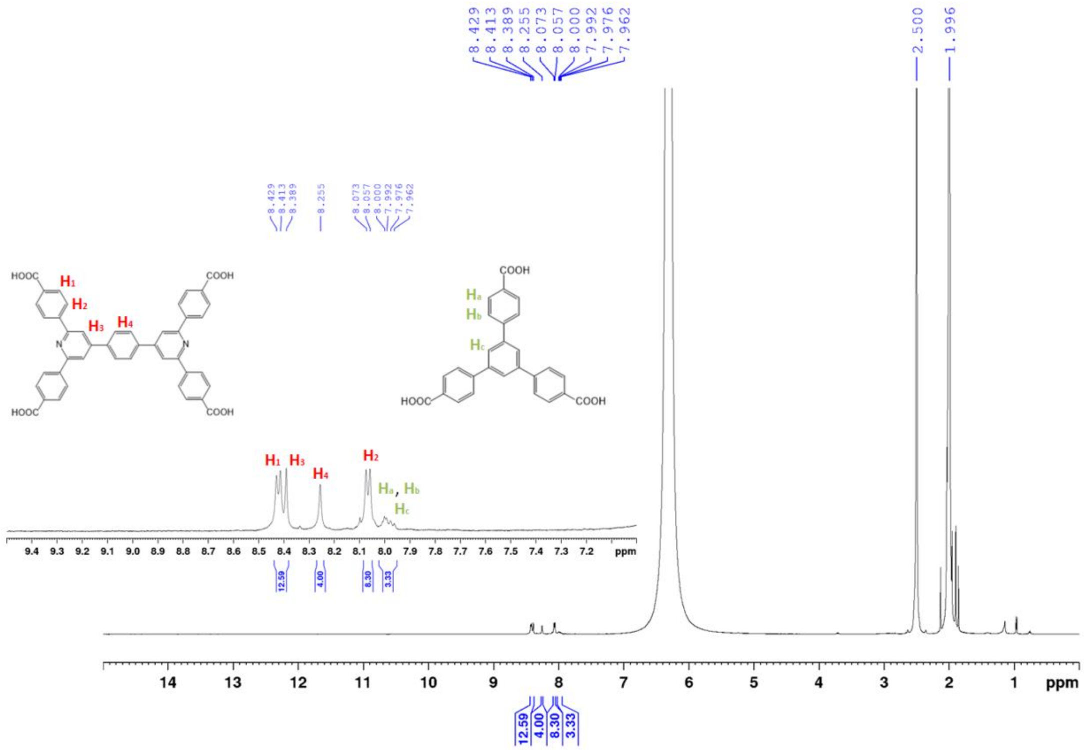

Figure S27. ^1H NMR spectrum of MeCN exchanged Fe-**tbb**-MOF-3 after digesting the sample in HCl/DMSO-d_6 solution.

BET area calculations using BETSI algorithm

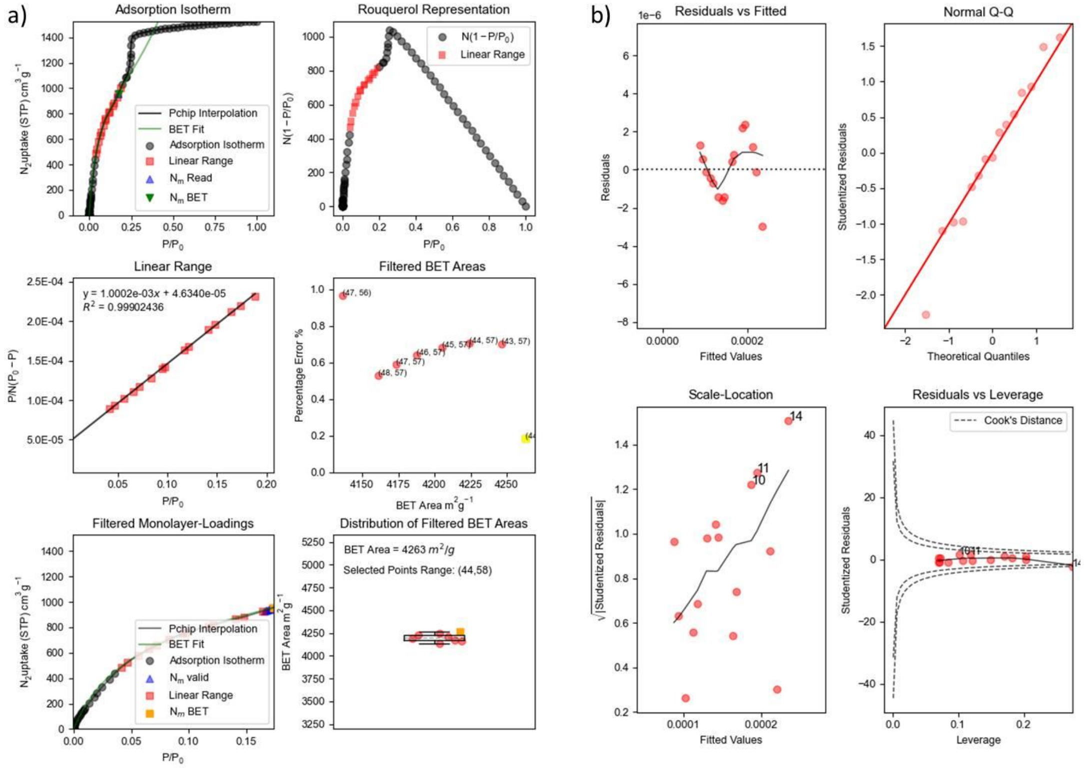

Figure S28. a) BETSI analysis for Fe-**tbb**-MOF-1 and b) the corresponding regression diagnostics.

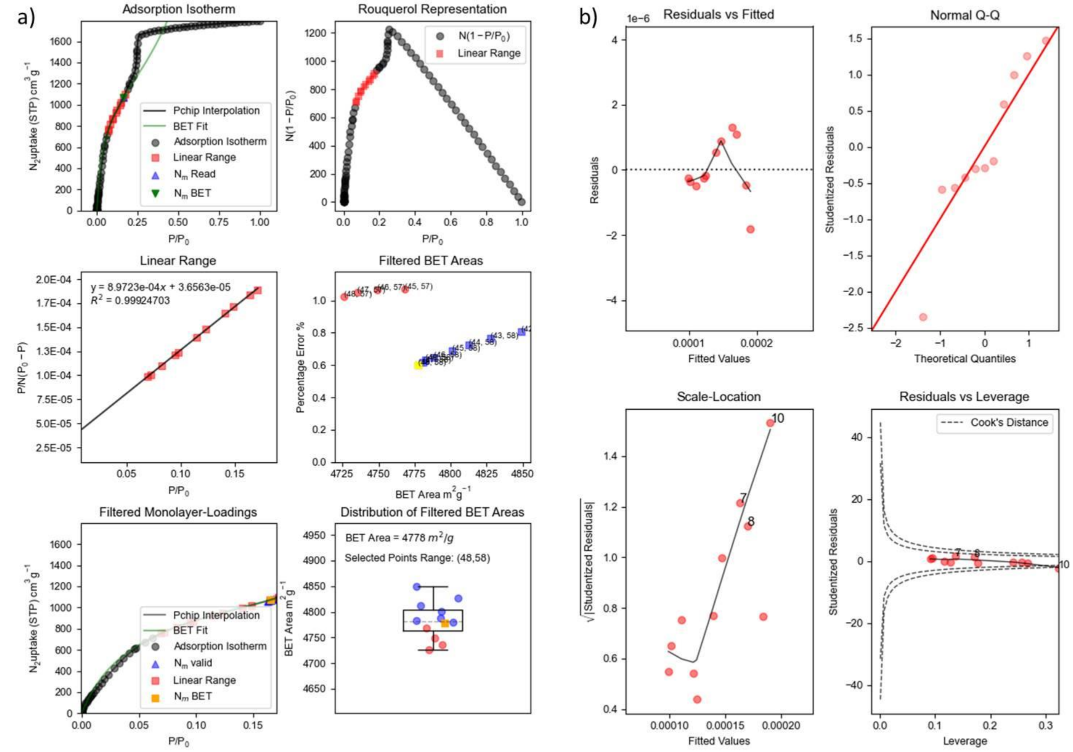

Figure S29. a) BETSI analysis for Fe-**tbb**-MOF-2 and b) the corresponding regression diagnostics.

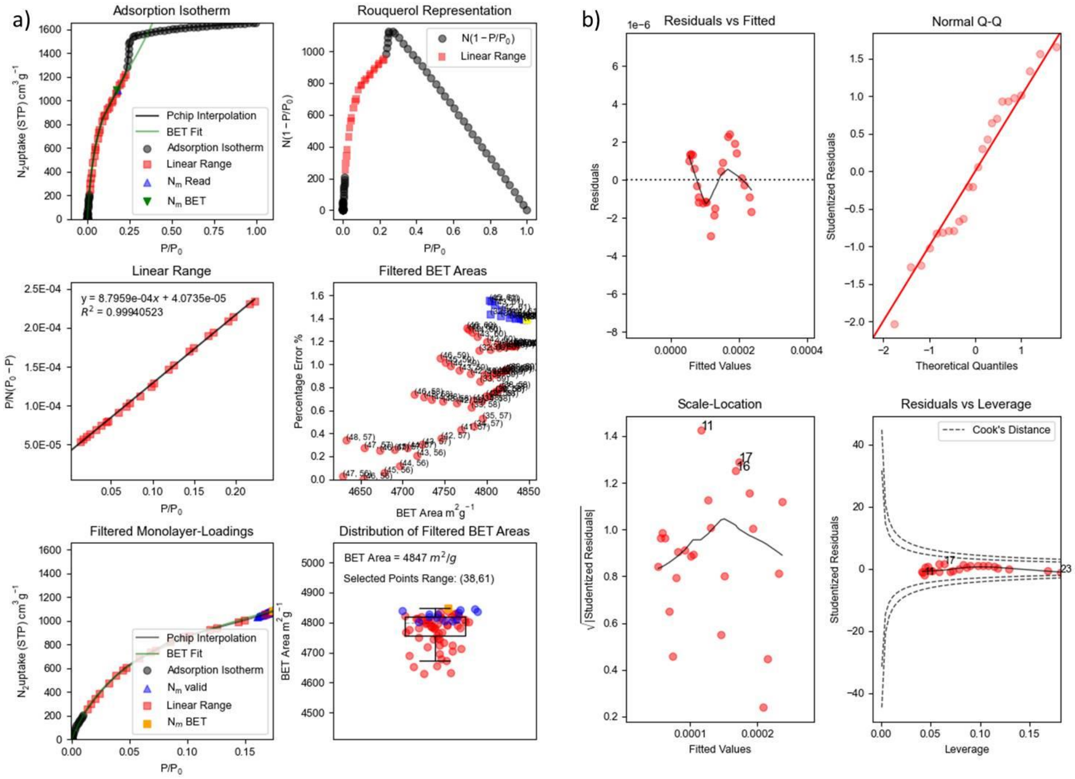

Figure S30. a) BETSI analysis for Fe-**tbb**-MOF-3 and b) the corresponding regression diagnostics.

Additional sorption isotherms

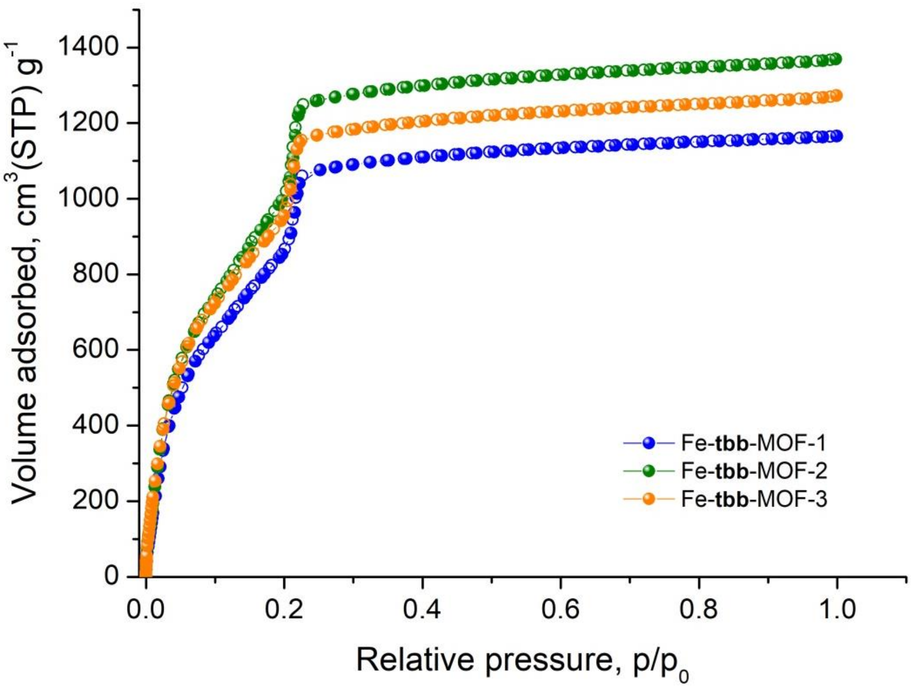

Figure S31. CH_4 sorption isotherms of Fe-**tbb**-MOF-x (x: 1, 2, 3) recorded at 112 K.

Table S2. Total pore volume of Fe-**tbb**-MOF-x (x: 1, 2, 3) at 0.99 p/p_0 obtained from the corresponding gas adsorption isotherm recorded at the respective boiling point.

Material	Ar at 87 K<br>$cm^3\ g^{-1}$	CH_4 at 112 K<br>$cm^3\ g^{-1}$	CO_2 at 195 K<br>$cm^3\ g^{-1}$
Fe-**tbb**-MOF-1	1.95	1.98	1.82
Fe-**tbb**-MOF-2	2.29	2.32	2.13
Fe-**tbb**-MOF-3	2.11	2.16	1.98

Thermogravimetric analysis (TGA)

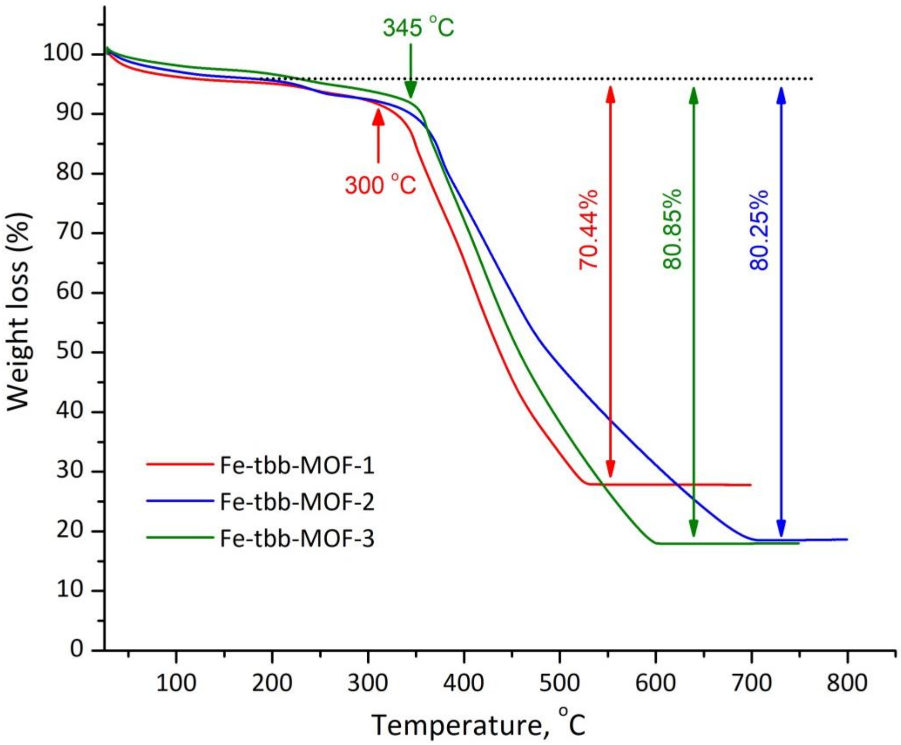

Figure S32. Thermogravimetric (TGA) curves of Fe-**tbb**-MOF-x (x: 1, 2, 3) recorded under nitrogen atmosphere with a heating rate of 5 °C/min.

High pressure H_2 and CH_4 sorption isotherms and determination of isosteric heat of adsorption

High pressure (0-100 bar) excess H_2 (at 77, 120 and 160K) and CH_4 (at 273, 288 and 298K) adsorption experiments were performed for Fe-**tbb**-MOF-1 and Fe-**tbb**-MOF-2 on an automated high-pressure volumetric apparatus (Hy Energy PCTPro 2000 by SETARAM) by using high purity gases (99.999%). The as prepared samples in DMF were first meticulously exchanged with acetonitrile (ACN) and finally left to settle. The supernatant ACN was removed and the remaining slurry was transferred with a pipette in a specially designed high pressure VCR® plug sample holder. The holder was attached with the aid of 10 µm fritted silver plated Ni-VCR® flange to an in-house constructed, valve-sealed, low dead volume reactor set-up. The reactor was then connected to a turbo vacuum pump station, equipped with liquid N_2/zeolite trap, and ACN was fully outgassed at room temperature. The reactor was isolated, detached from the pumping station and attached to the PCTPro system, were additional "in-situ" outgassing was carried out by heating to 80 °C with a rate of 1.5 °C/min under high vacuum (< 10^{-6} mbar) for at least 12 hours.

Sample holder void (dead) volume calibrations were conducted using low pressure (P< 3.5 bar) helium (99.999%) expansions. For each experiment two "apparent" dead volume calibrations (each average of 10 independent measurements) were performed: a) V_{res} at the gas dosing reservoir temperature (T_{res}=30°C) by ensuring isothermal conditions (30 °C) in all parts of the instrument including the sample and b) V_{exp}, at the experimental conditions (i.e. the sample and part of the reactor kept at the measurement temperature, T_{exp}, while all the other volumes of the system at T_{res}). The above calibrations allow the determination of V_h and V_c, i.e. the "hot" and "cold" dead volumes during the experiment. In more detail, $V_c=T_{exp} \cdot(V_{exp}-V_{res})/(Tres-T_{exp})$ and $V_h=V_{res}-V_c$. Nevertheless, at cryogenic temperatures (77, 120 and 160K), in order to avoid helium adsorption volume calibration errors (which were found to be significant), V_{exp} apparent volumes were calculated by means of previously constructed reference curves. More specifically, the curves, (V_{exp} at 77, 120 and 160 K as a function of V_{res}) were obtained by inserting various amounts (and therefore volumes) of non-adsorbing material (Pyrex glass) and performing series of V_{res} and V_{exp} measurements.

For near ambient CH_4 measurements a circulating bath was employed, while for H_2 measurements liquid N_2 was used as cryogen. For 77K measurements, the sample cell was immersed in liquid nitrogen (LN2); the LN2 level was kept constant by means of a custom made auto-refill controller. For the 120 and 160K experiments, the sample cell was inserted in an in-house built aluminium "sleeve" holder which was equipped with heating elements and immersed in LN2 (again the LN2 level was constantly controlled as explained). For all the measurements, real gas corrections were carried out (at both T_{res} and T_{exp}) by using NIST compressibility factors[5]. It should also be mentioned that blank measurements were also carried on the empty sample holder under identical experimental conditions to the actual sample experiments; in all cases the "blank" amounts were found to be insignificant.

The excess and total amounts of H_2 adsorbed are presented on a gravimetric basis as wt% [mass of H_2/(mass of H_2+mass of MOF)] and volumetrically (g L^{-1}). For the case of CH_4 the gravimetric uptakes are reported as mg g^{-1} while the volumetric as cm^3 (STP) cm^{-3}. Total amounts have been calculated as $n_T=n_{ex}+\rho_g V_p$, where n_T: total moles in the pores, n_{ex}: excess moles measured, ρ_g: density of the gas phase, V_p: pore volume (as deduced from Argon isotherms at 87K).

Isosteric heats of adsorption (Q_{st}) were calculated for Fe-**tbb**-MOF-1 and Fe-**tbb**-MOF-2 using the virial coefficient method. Total adsorption isotherms measured at three different temperatures were fitted by the virial type equation which is given below:

$$lnP = lnN + \left(\frac{1}{T}\right)\sum_{i=0}^{m} a_i\, N^i + \sum_{i=0}^{n} b_i\, N^i \qquad (1)$$

where P is the equilibrium pressure, N is the total adsorbed amount, T is the temperature, α_i and b_i are the virial coefficients and m, n are the numbers of coefficients used to adequately describe the isotherms.

Subsequently the derived virial coefficients α_i and b_i were used in order to determine heat of adsorption for zero coverage (Q_{st0}) as well as the heat of adsorption as a function of the total adsorbed amount according to the following two equations:

$$Q_{st0} = -Ra_0 \qquad (2)$$

$$Q_{st}(N) = -R\sum_{i=0}^{m} a_i N^i \qquad (3)$$

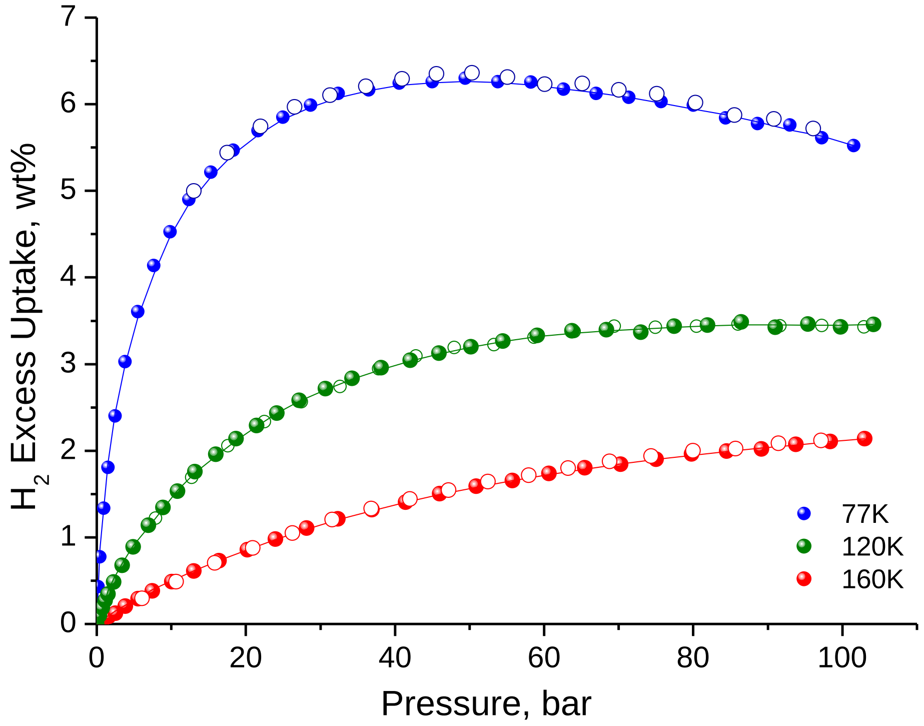

Figure S33: H_2 excess gravimetric adsorption isotherms for Fe-**tbb**-MOF-1.

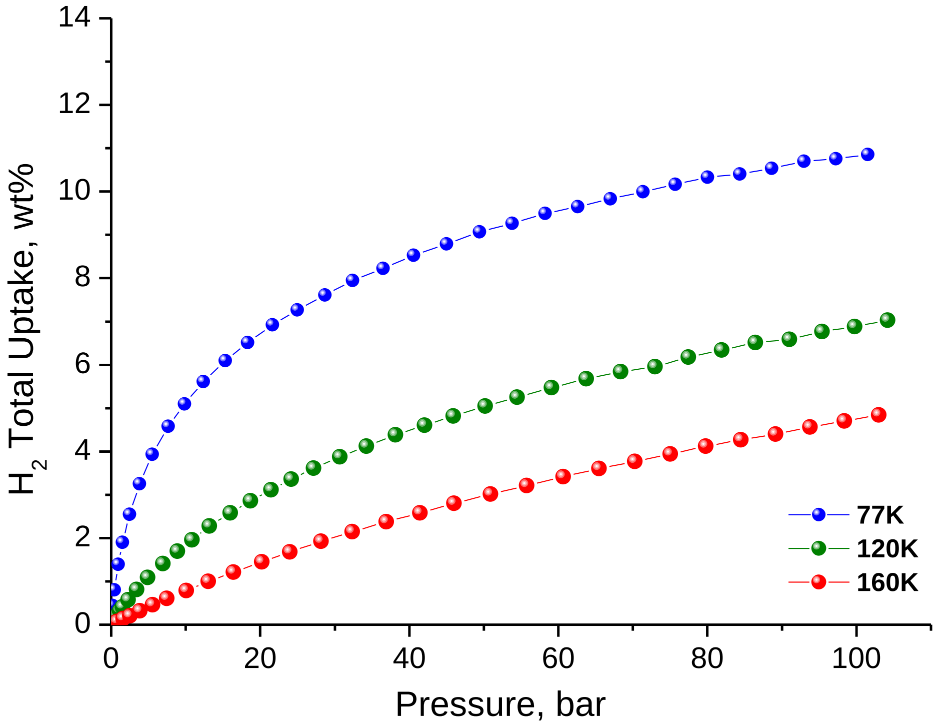

Figure S34: H_2 total gravimetric adsorption isotherms for Fe-**tbb**-MOF-1.

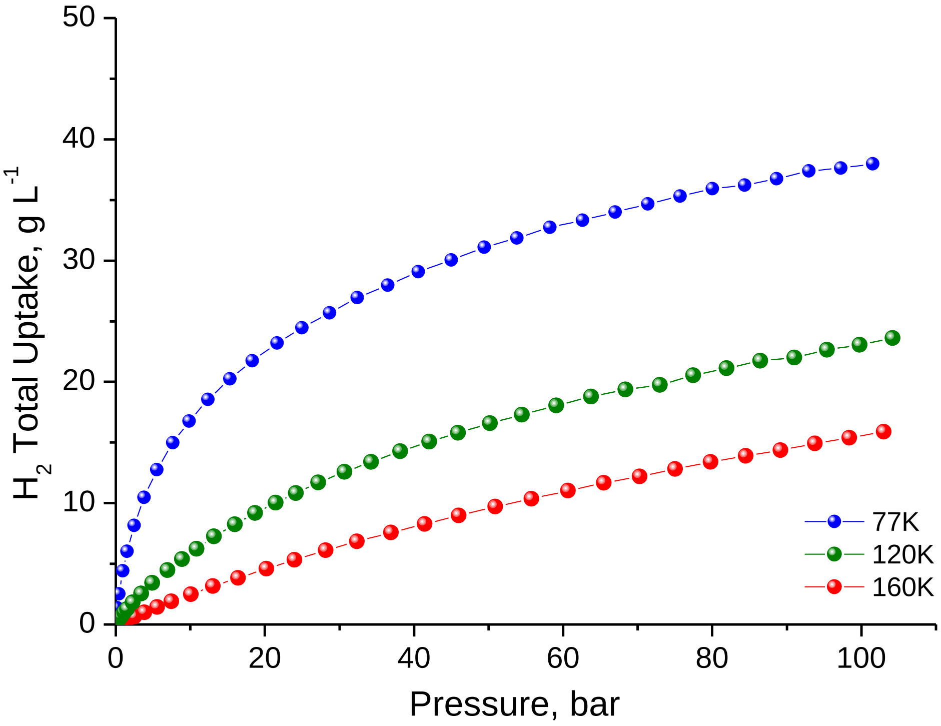

Figure S35: H_2 total volumetric adsorption isotherms for Fe-**tbb**-MOF-1.

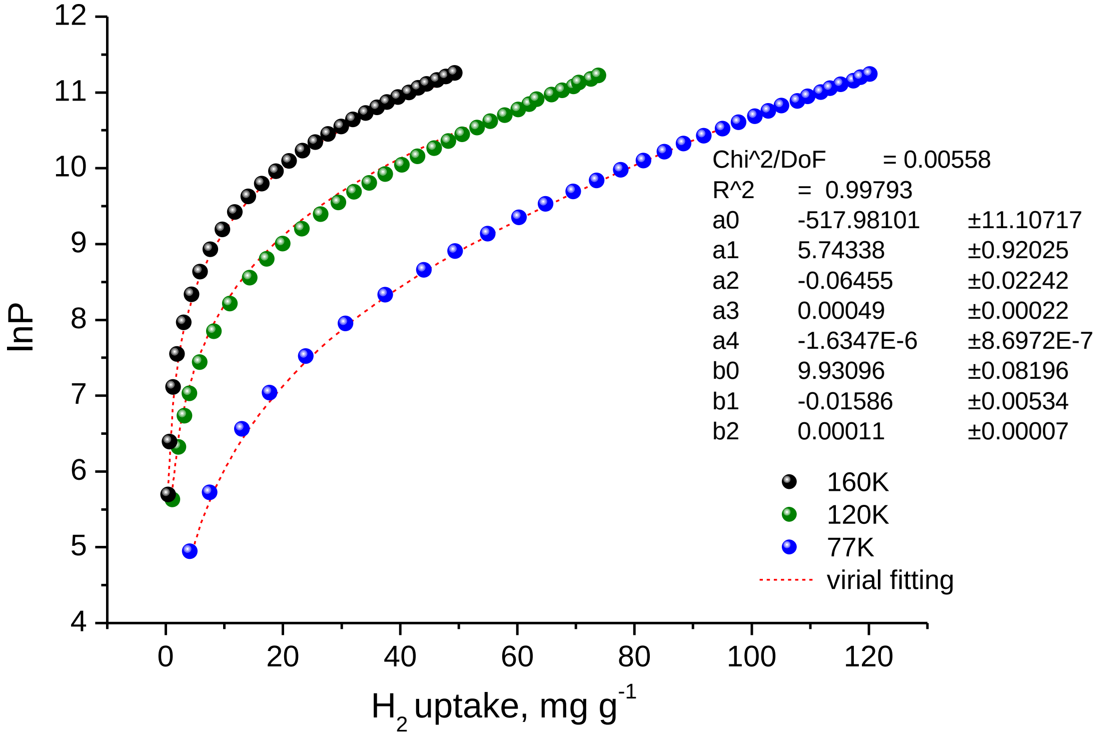

Figure S36: Virial fitting of H_2 adsorption isotherms of Fe-**tbb**-MOF-1.

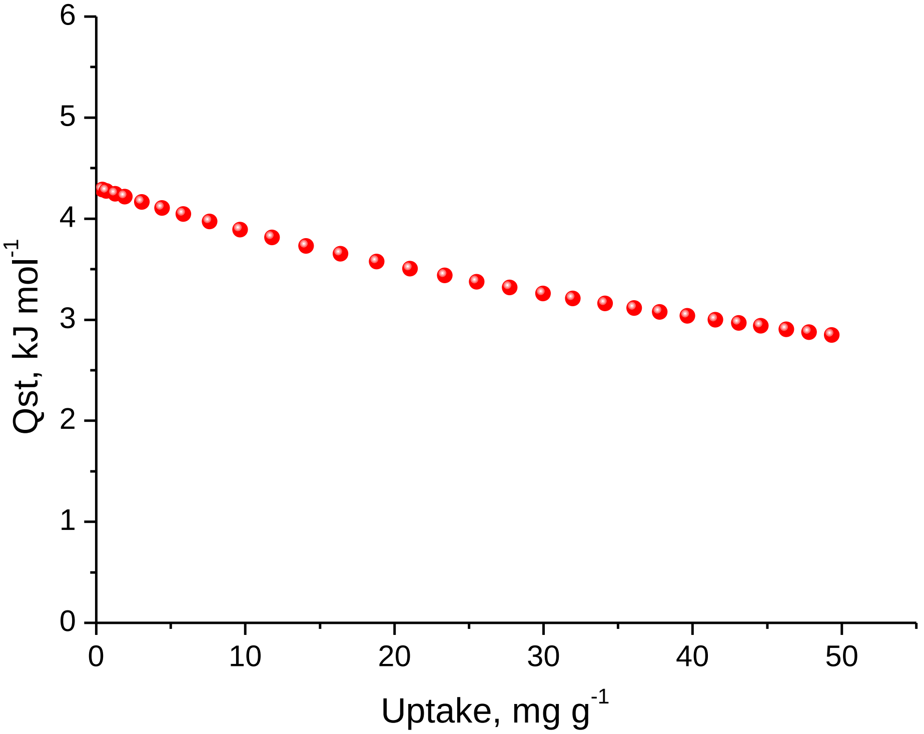

Figure S37: H_2 isosteric heat of adsorption in Fe-**tbb**-MOF-1 as a function of coverage

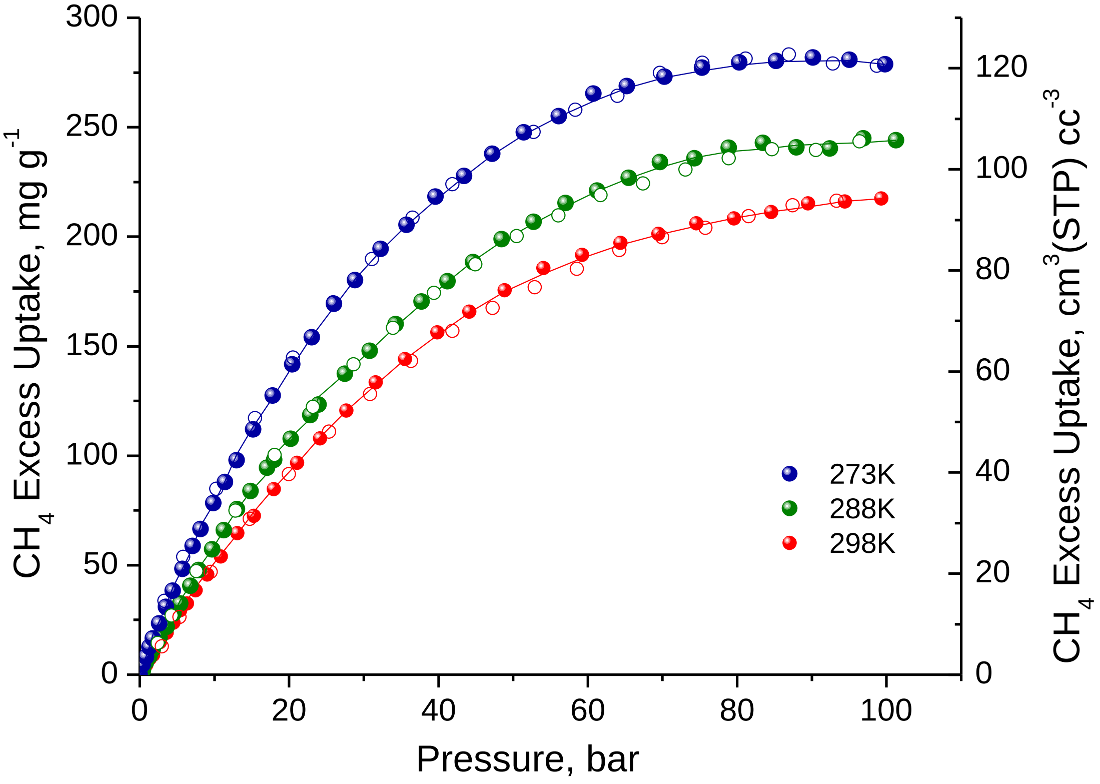

Figure S38: CH_4 excess gravimetric (left y-axis) and volumetric (right y-axis) adsorption isotherms for Fe-**tbb**-MOF-1.

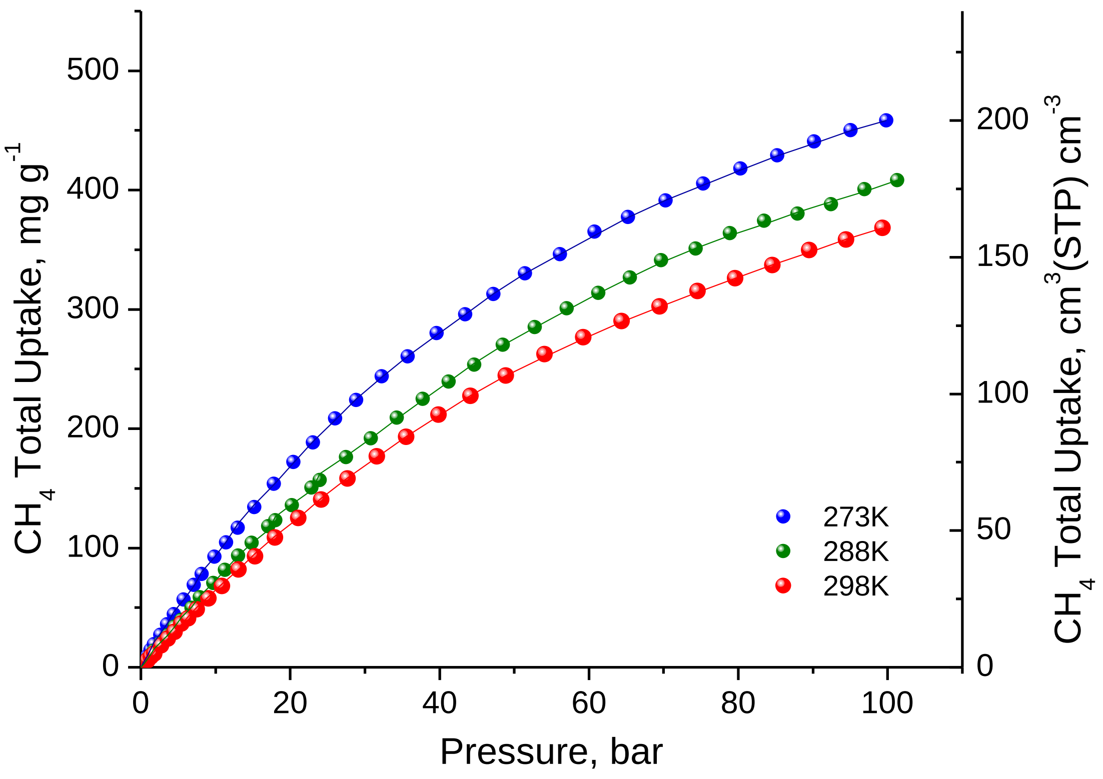

Figure S39: CH_4 total gravimetric (left y-axis) and volumetric (right y-axis) adsorption isotherms for Fe-**tbb**-MOF-1.

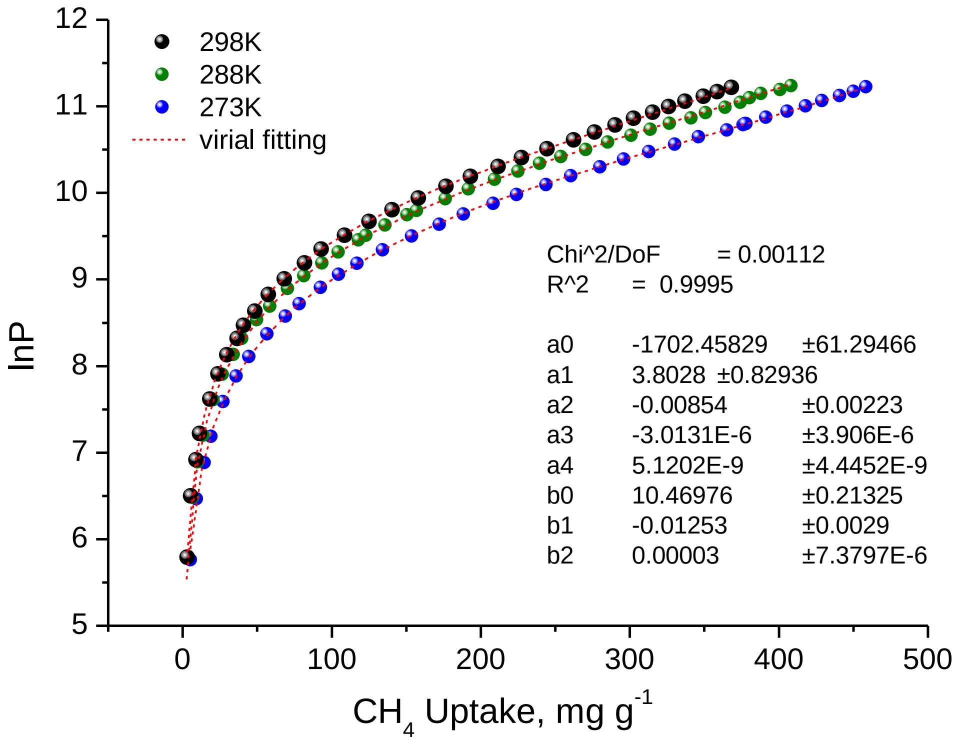

Figure S40: Virial type fitting of CH_4 adsorption isotherms of Fe-**tbb**-MOF-1.

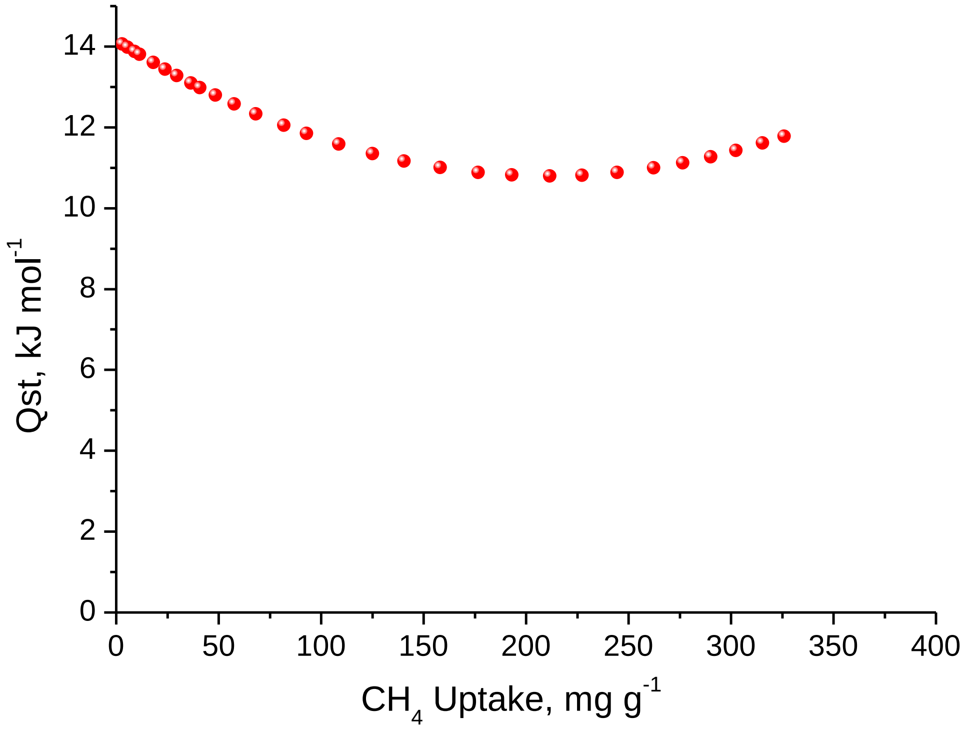

Figure S41: CH_4 isosteric heat of adsorption in Fe-**tbb**-MOF-1 as a function of coverage.

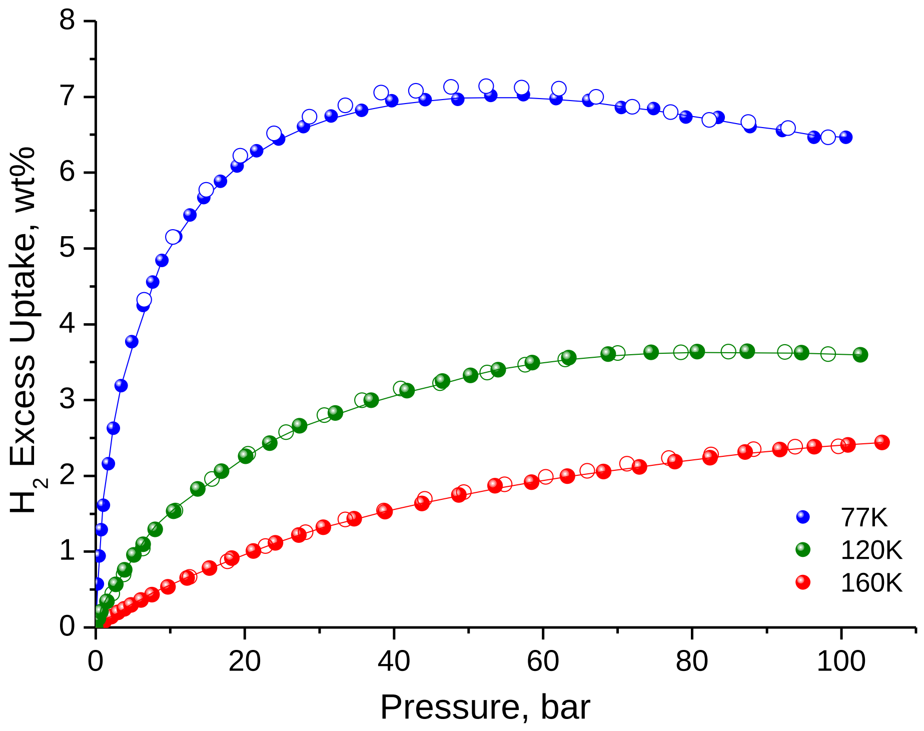

Figure S42: H_2 excess gravimetric adsorption isotherms for Fe-**tbb**-MOF-2.

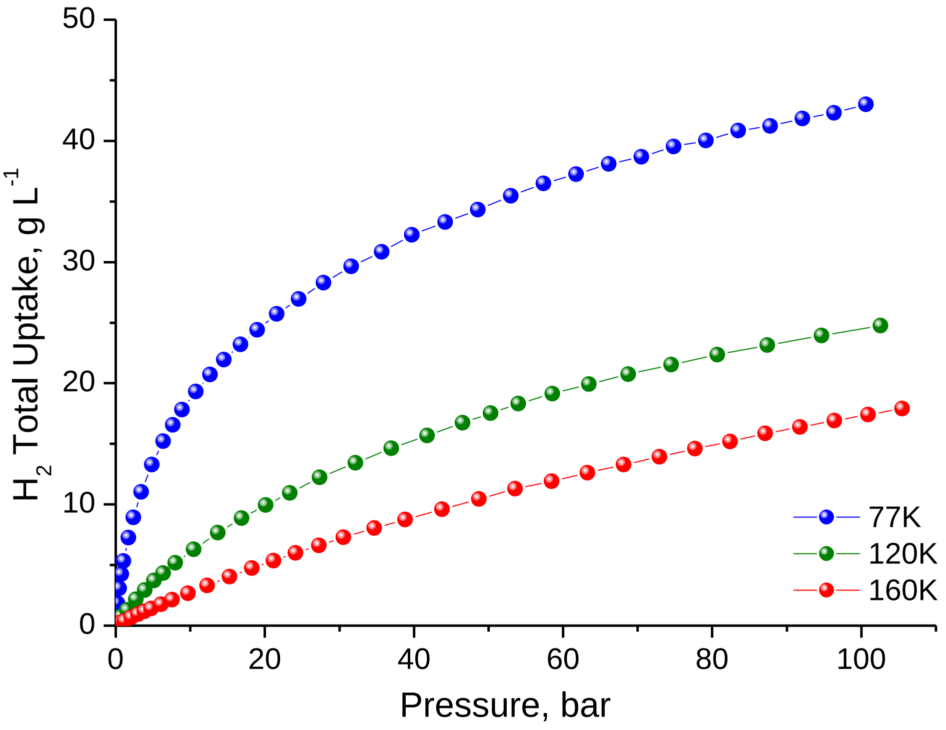

Figure S43: H_2 total volumetric adsorption isotherms for Fe-**tbb**-MOF-2.

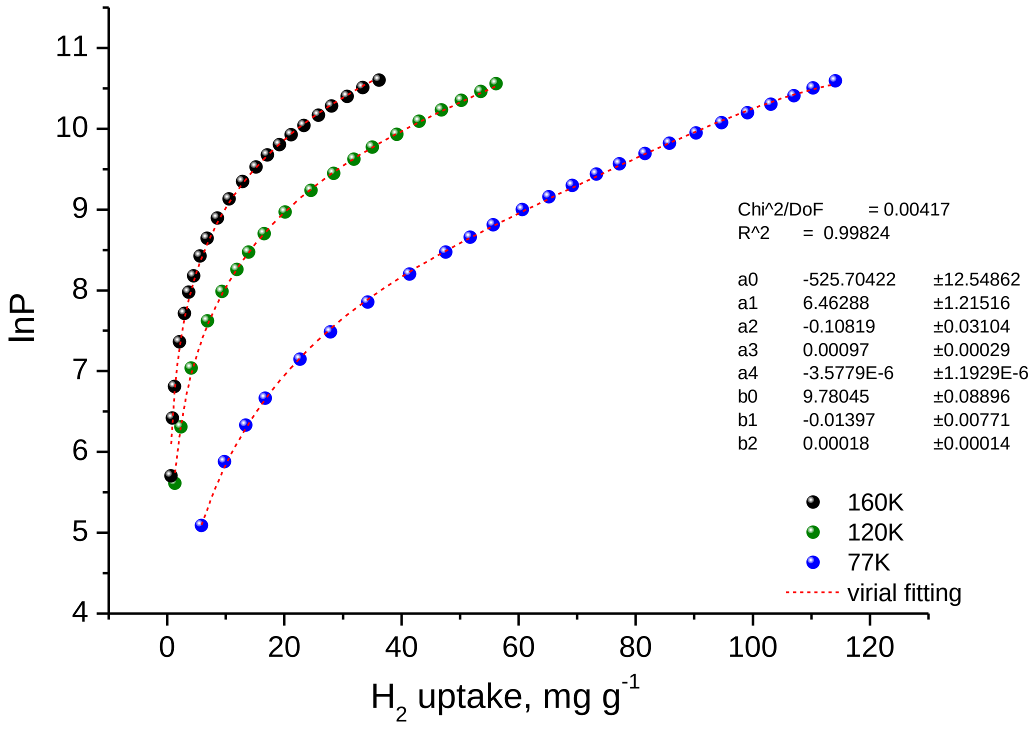

Figure S44: Virial type fitting of H_2 adsorption isotherms of Fe-**tbb**-MOF-2.

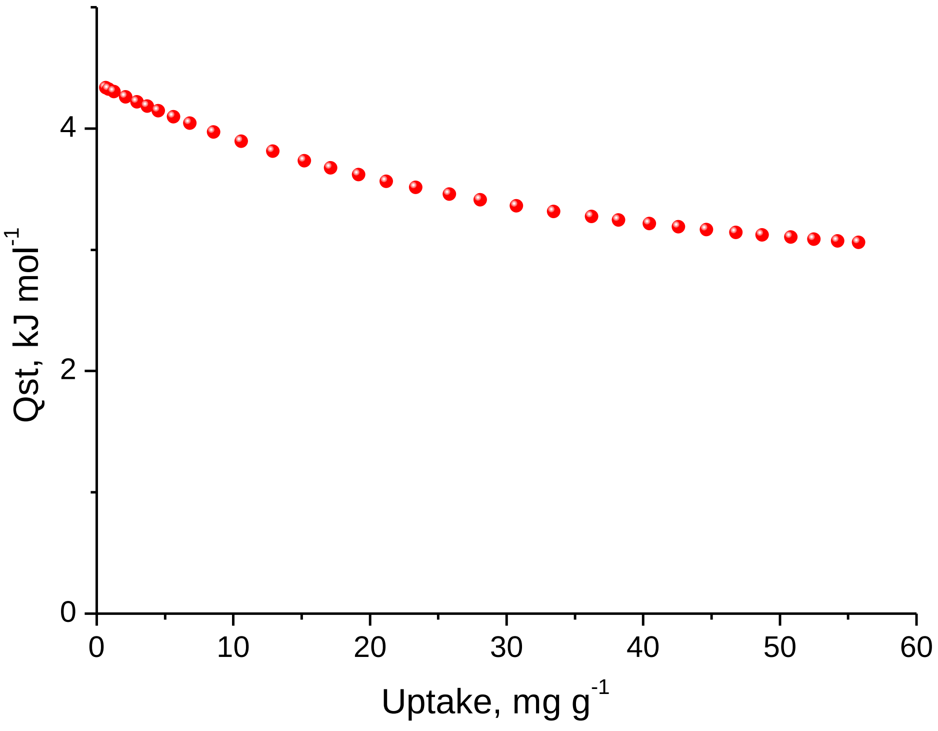

Figure S45: H_2 isosteric heat of adsorption in Fe-**tbb**-MOF-2 as a function of coverage

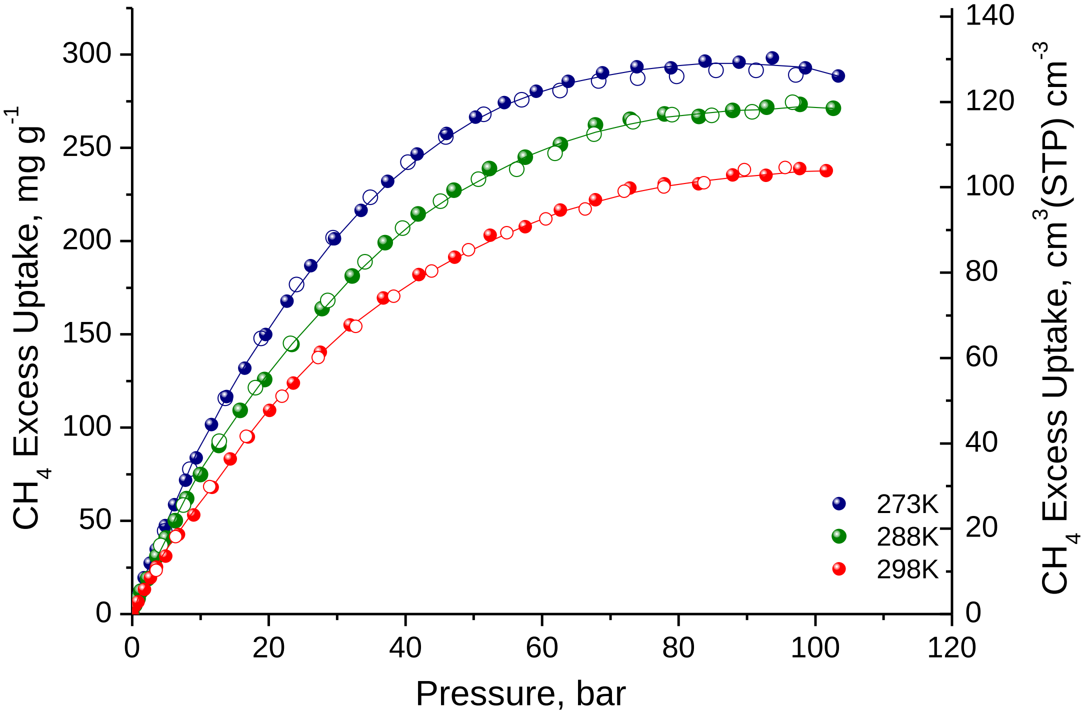

Figure S46: CH_4 excess gravimetric (left y-axis) and volumetric (right y-axis) adsorption isotherms for Fe-**tbb**-MOF-2.

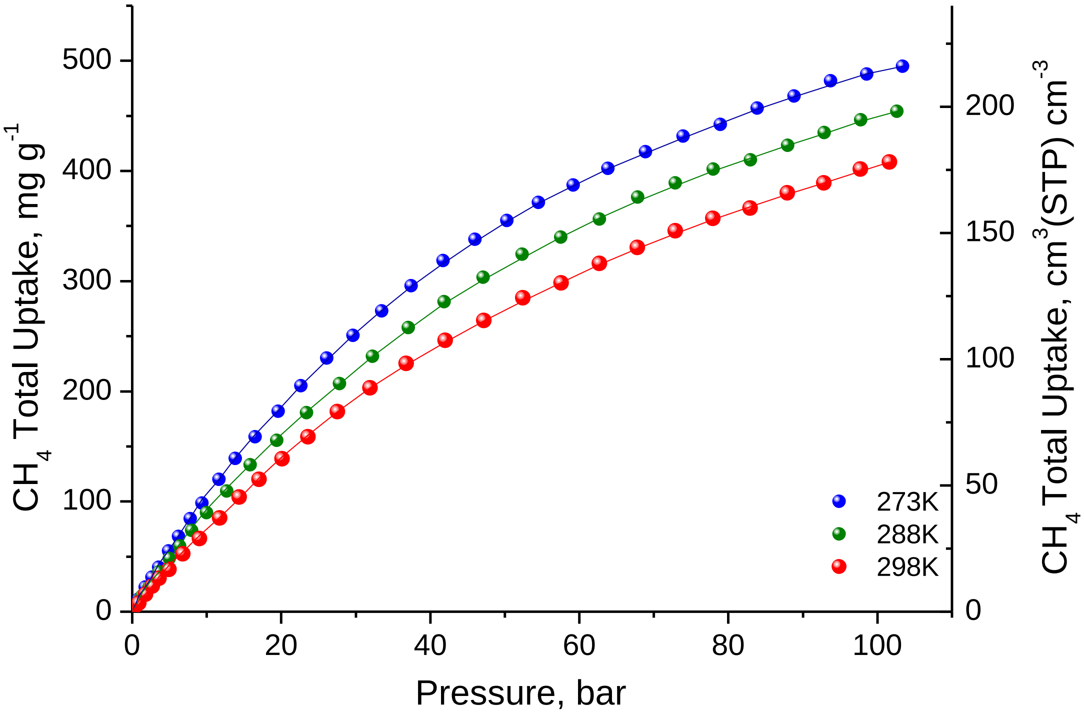

Figure S47: CH_4 total gravimetric (left y-axis) and volumetric (right y-axis) adsorption isotherms for Fe-**tbb**-MOF-2.

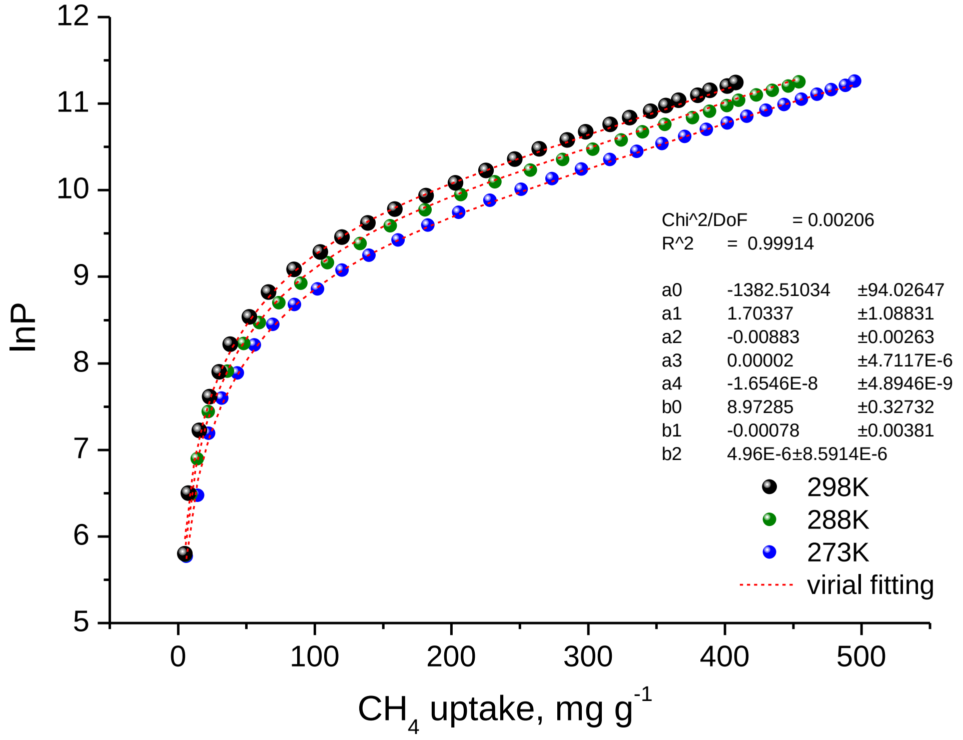

Figure S48: Virial fitting of CH_4 adsorption isotherms of Fe-**tbb**-MOF-2.

Table S3. Gravimetric and volumetric H_2 deliverable capacity under combined temperature and pressure swing conditions (77 K/100 bar →160 K/5 bar) for Fe-**tbb**-MOF-2 and representative best performing MOFs.

Material	BET surface area (m^2 g^{-1})	Total pore volume (cm^3 g^{-1})	Crystallographic density (g cm^{-3})	H_2 working gravimetric capacity (wt%)	H_2 working volumetric capacity (g L^{-1})	Reference
Fe-**tbb**-MOF-2	4777	2.29	0.312	11.6	41.4	This work
NU-1500-Al	3560	1.46	0.498	8.2	44.6	11
NU-1501-Al	7310	2.91	0.283	14.0	46.2	11
NU-1501-Fe	7140	2.90	0.299	13.2	45.4	11
NU-100/PCN-610	6050	3.17	0.29	13.9	47.6	6
SNU-70	4944	2.14	0.411	10.6	47.9	6
UMCM-9	5039	2.31	0.37	11.3	47.4	6
MOF-5	3512	1.36	0.59	7.8	51.9	7
IRMOF-20	4073	1.65	0.51	9.1	51.0	7
NU-1101	4340	1.72	0.459	9.1	46.6	8
NU-1102	3720	1.65	0.403	9.6	43.7	8
NU-1103	6245	2.72	0.298	12.6	43.2	8
NU-125	3230	1.33	0.578	8.5	49	9
NOTT-112	3440	1.44	0.446	9.1	41	9
NU-1000	2200	1.48	0.571	8.3	48	9
PCN-250	1780	0.71	0.896	5.2	47	9
MFU-4l-Li	4070	1.66	0.479	9.4	50.2	10

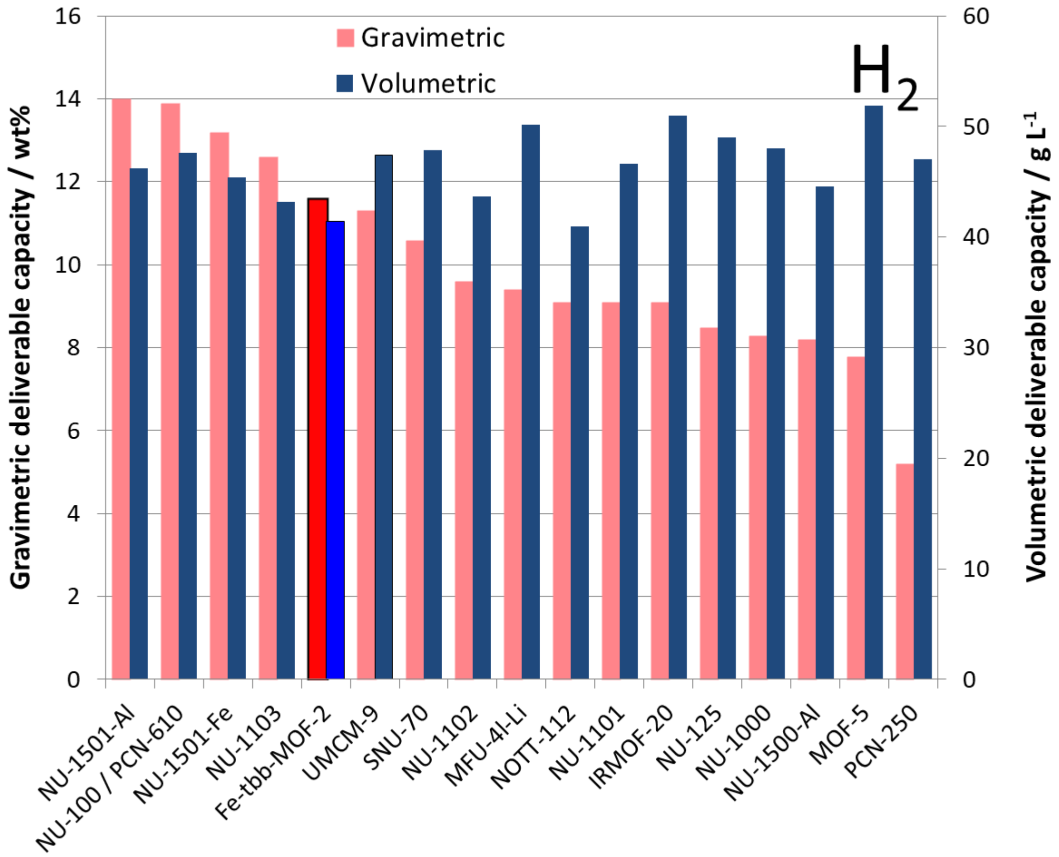

Figure S49. Gravimetric and volumetric H_2 deliverable capacity under combined temperature and pressure swing conditions (77 K/100 bar →160 K/5 bar) for Fe-**tbb**-MOF-2 and representative best performing MOFs.

Table S4. Gravimetric and volumetric CH_4 deliverable capacity under pressure swing conditions for Fe-**tbb**-MOF-2 and representative best performing MOFs.

Material	BET surface area (m^2 g^{-1})	PV (cm^3 g^{-1})	Crystal density (g cm^{-3})	T (K)	Pressure swing conditions (bar)	CH_4 working gravimetric capacity (g g^{-1})	CH_4 working volumetric capacity (cm^3 cm^{-3})	Ref.
Fe-**tbb**-MOF-2	4777	2.29	0.312	298	5-100	0.367	160	This work
NU-1500-Al	3560	1.46	0.498	296	5-100	0.29	202	11
NU-1501-Al	7310	2.91	0.283	296	5-100	0.50	198	11
NU-1501-Fe	7140	2.90	0.299	296	5-100	0.48	201	11
Al-**soc**-MOF-1	5585	2.30	0.34	298	5-80	0.42	201	12
UTSA-76a	2820	1.09	0.699	298	5-65	0.201	197	13
HKUST-1	2203[a]	0.77	0.881	298	5-100	0.17	207	14
Ni-MOF-74	1593[a]	0.56	1.195	298	5-100	0.10	162	14
NU-111	4930	2.09	0.409	298	5-65	0.31	177	15
MOF-210	6240	3.60	0.25	298	5-80	0.45	157	16
MOF-905	3490	1.34	0.549	298	5-80	0.26	203	17
ST-2	5172	2.44	0.366	298	5-80	0.36	185	18
MOF-519	2400	0.94	0.953	298	5-100	0.19	251	19
NPF-200	5463	2.17	0.389	298	5-100	0.38	207	20
MFU-4l-Li	4070	1.66	0.479	296	5-100	0.33	220	10

[a] Langmuir surface area

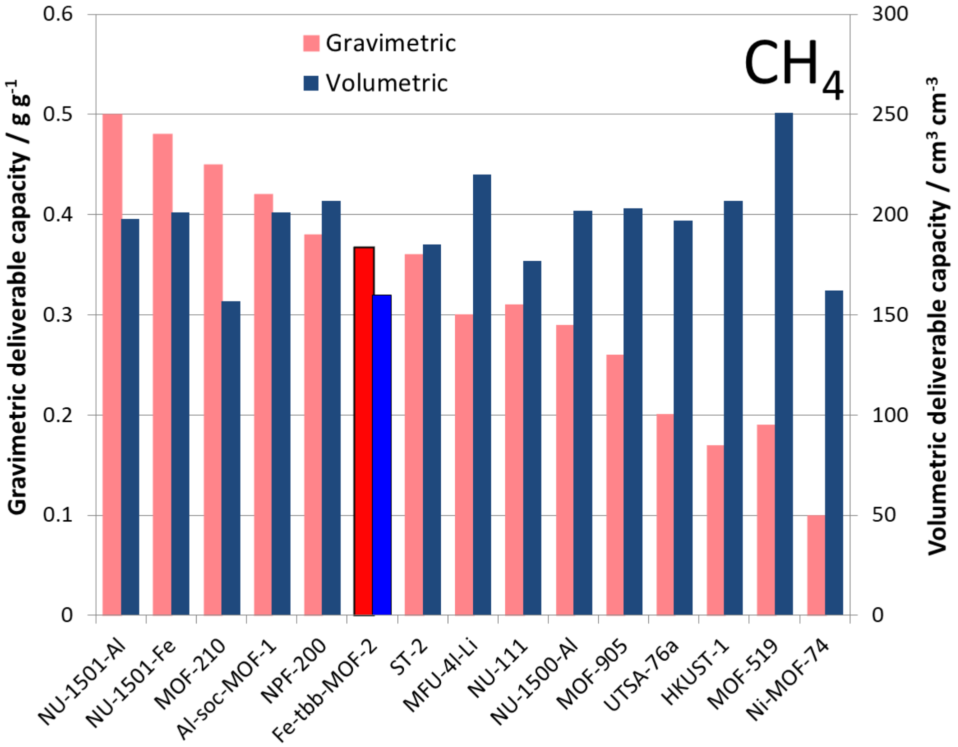

Figure S50. Gravimetric and volumetric CH_4 deliverable capacity under pressure swing conditions for Fe-**tbb**-MOF-2 and representative best performing MOFs.

References

[1] Yang, J. X.; Tao, X. T.; Chun, X. Y.; Yun, X. Y.; Wang, L.; Liu, Z.; Ren, Y.; Min, H. J., A facile synthesis and properties of multicarbazole molecules containing multiple vinylene bridges. *J. Am. Chem. Soc.* 2005, **127**, 3278–3279.

[2] Feng, D.; Wang, K.; Su, J.; Liu, T. F.; Park, J.; Wei, Z.; Bosch, M.; Yakovenko, A.; Zou, X.; Zhou, H. C., A highly stable zeotype mesoporous zirconium metal-organic framework with ultralarge pores. *Angew. Chem., Int. Ed.* 2015, **54**(1), 149–154.

[3] Zhang, Y.; Yang, X.; Zhou, H. C., Direct synthesis of functionalized PCN-333 via linker design for Fe3+ detection in aqueous media. Dalton Trans. 2018, **47**(34), 11806–11811.

[4] Tsangarakis, C.; Azmy, A.; Tampaxis, C.; Zibouche, N.; Klontzas, E.; Tylianakis, E.; Froudakis, G. E.; Steriotis, T.; Spanopoulos, I.; Trikalitis, P. N., Water-Stable etb-MOFs for Methane and Carbon Dioxide Storage. *Inorg. Chem.* **2023,** *62* (14), 5496-5504.

[5] EoS for H_2: Younglove, B.A., J. Phys. Chem. Ref. Data, Vol. 11, Suppl. 1, pp. 1-11, 1982 and for CH_4: Setzmann, U. and Wagner, W., J. Phys. Chem. Ref. Data, 20(6):1061-1151, 1991.

[6] Ahmed, A.; Seth, S.; Purewal, J.; Wong-Foy, A. G.; Veenstra, M.; Matzger, A. J.; Siegel, D. J., Exceptional hydrogen storage achieved by screening nearly half a million metal-organic frameworks. *Nat. Commun.* **2019**, *10*(1), 1568.

[7] Ahmed, A.; Liu, Y.; Purewal, J.; Tran, L. D.; Wong-Foy, A. G.; Veenstra, M.; Matzger, A. J.; Siegel, D. J., Balancing gravimetric and volumetric hydrogen density in MOFs. *Energy Environ. Sci.* **2017**, *10*(11), 2459–2471.

[8] Gómez-Gualdrón, D. A.; Wang, T. C.; García-Holley, P.; Sawelewa, R. M.; Argueta, E.; Snurr, R. Q.; Hupp, J. T.; Yildirim, T.; Farha, O. K., Understanding volumetric and gravimetric hydrogen adsorption trade-off in metal-organic frameworks. *ACS Appl. Mater. Interfaces* **2017**, *9*(39), 33419–33428.

[9] García-Holley, P.; Schweitzer, B.; Islamoglu, T.; Liu, Y.; Lin, L.; Rodriguez, S.; Weston, M. H.; Hupp, J. T.; Gómez-Gualdrón, D. A.; Yildirim, T.; Farha, O. K., Benchmark Study of Hydrogen Storage in Metal-Organic Frameworks under Temperature and Pressure Swing Conditions. *ACS Energy Lett.* **2018**, *3*(3), 748-754.

[10] Chen, Z.; Mian, M. R.; Lee, S. J.; Chen, H.; Zhang, X.; Kirlikovali, K. O.; Shulda, S.; Melix, P.; Rosen, A. S.; Parilla, P. A.; Gennett, T.; Snurr, R. Q.; Islamoglu, T.; Farha, O. K., Fine-tuning a robust metal-organic framework toward enhanced clean energy gas storage. *J. Am. Chem. Soc.* **2021**, *143*(45), 18838–18843.
[11] Chen, Z.; Li, P.; Anderson, R.; Wang, X.; Zhang, X.; Robison, L.; Redfern, L. R.; Moribe, S.; Islamoglu, T.; Gómez-Gualdrón, D. A.; Yildirim, T.; Stoddart, J. F.; Farha, O. K., Balancing volumetric and gravimetric uptake in highly porous materials for clean energy. *Science* **2020**, *368*(6488), 297–303.
[12] Alezi, D.; Belmabkhout, Y.; Suyetin, M.; Bhatt, P. M.; Weseliński, Ł. J.; Solovyeva, V.; Adil, K.; Spanopoulos, I.; Trikalitis, P. N.; Emwas, A.-H.; Eddaoudi, M., MOF Crystal Chemistry Paving the Way to Gas Storage Needs: Aluminum-Based soc-MOF for CH4, O2, and CO2 Storage. *J. Am. Chem. Soc.* **2015**, *137* (41), 13308-13318.
[13] Li, B.; Wen, H. M.; Wang, H.; Wu, H.; Tyagi, M.; Yildirim, T.; Zhou, W.; Chen, B., A porous metal-organic framework with dynamic pyrimidine groups exhibiting record high methane storage working capacity. *J. Am. Chem. Soc*. **2014**, *136*(17), 6207–6210.
[14] Mason, J. A.; Veenstra, M.; Long, J. R., Evaluating metal-organic frameworks for natural gas storage. *Chem. Sci.* **2014**, *5*(1), 32-51.
[15] Peng, Y.; Srinivas, G.; Wilmer, C. E.; Eryazici, I.; Snurr, R. Q.; Hupp, J. T.; Yildirim, T.; Farha, O. K., Simultaneously high gravimetric and volumetric methane uptake characteristics of the metal-organic framework NU-111. *Chem. Commun.* **2013**, *49*(29), 2992–2994.
[16] Furukawa, H.; Ko, N.; Go, Y. B.; Aratani, N.; Choi, S. B.; Choi, E.; Yazaydin, A. Ö.; Snurr, R. Q.; O'Keeffe, M.; Kim, J.; Yaghi, O. M., Ultrahigh porosity in metal-organic frameworks. *Science* **2010**, *329*(5990), 424–428.
[17] Jiang, J.; Furukawa, H.; Zhang, Y. B.; Yaghi, O. M., High Methane Storage Working Capacity in Metal-Organic Frameworks with Acrylate Links. *J. Am. Chem. Soc*. **2016**, *138*(32), 10244–10251.
[18] Liang, C. C.; Shi, Z. L.; He, C. T.; Tan, J.; Zhou, H. D.; Zhou, H. L.; Lee, Y.; Zhang, Y. B., Engineering of Pore Geometry for Ultrahigh Capacity Methane Storage in Mesoporous Metal-Organic Frameworks. *J. Am. Chem. Soc*. **2017**, *139*(38), 13300–13303.
[19] Gándara, F.; Furukawa, H.; Lee, S.; Yaghi, O. M., High Methane Storage Capacity in Aluminum Metal–Organic Frameworks. *J. Am. Chem. Soc.* **2014**, *136* (14), 5271-5274.
[20] Zhang, X.; Lin, R. B.; Alothman, Z. A.; Alduhaish, O.; Yildirim, T.; Zhou, W.; Li, J. R.; Chen, B., Promotion of methane storage capacity with metal-organic frameworks of high porosity. *Inorg. Chem. Front.* **2023**, *10*(2), 454–459.
